# Enantio‐ and Diastereoselective Synthesis and Spiral‐Stair‐Like Single Helix Assembly of Figure‐Eight Cyclophenylenes

**DOI:** 10.1002/anie.202502764

**Published:** 2025-04-07

**Authors:** Kohei Adachi, Juntaro Nogami, Daisuke Hashizume, Daiki Tauchi, Masashi Hasegawa, Ken Tanaka

**Affiliations:** ^1^ Department of Chemical Science and Engineering Institute of Science Tokyo O‐okayama, Meguro‐ku Tokyo 152–8550 Japan; ^2^ Department of Molecular and Macromolecular Chemistry Graduate School of Engineering Nagoya University Furo‐cho, Chikusa‐ku Nagoya Aichi 464–8603 Japan; ^3^ RIKEN Center for Emergent Matter Science (CEMS) 2‐1 Hirosawa Wako Saitama 351‐0198 Japan; ^4^ Graduate School of Science Kitasato University Sagamihara Kanagawa 252–0373 Japan

**Keywords:** [2 + 2 + 2] Cycloaddition, Asymmetric synthesis;Figure‐eight cyclophenylenes, Rhodium, Spiral‐stair‐like single helix assembly

## Abstract

Helix assemblies of chiral molecules can transfer microscopic unimolecular chirality to macroscopic supramolecular chirality, enhancing various chiral properties. In addition to the commonly observed spiral‐column‐like helix assembly, a small number of spiral‐stair‐like helix assemblies have also been reported in aromatic nanocarbons with multiple chirality‐related irregularities. However, they require separation of diastereomers and/or enantiomers or do not have stable chirality. Here, we report the enantio‐ and diastereoselective synthesis of figure‐eight [10]cyclophenylenes with stable helical chirality by the rhodium‐catalyzed four consecutive intramolecular [2 + 2 + 2] cycloadditions of dodecaynes with two flexible biphenyl units. The chiral figure‐eight [10]cyclophenylene with ethyl and methyl side chains exhibits the spiral‐stair‐like single helix assembly in the crystal due to CH–π and CH–O interactions and good CPL properties in solution. Experimental verification of the enantio‐ and diastereodetermining steps of four consecutive [2 + 2 + 2] cycloadditions is also reported.

## Graphical abstract

We present here the enantio‐ and diastereoselective synthesis of chiral figure‐eight [10]cyclophenylenes via the rhodium‐catalyzed four consecutive intramolecular [2 + 2 + 2] cycloadditions of dodecaynes with two flexible biphenyl units, resulting in increased rotational freedom of the reaction intermediates. The figure‐eight [10]cyclophenylene with ethyl and methyl side chains exhibits the spiral‐stair‐like single helix assembly in the crystal due to CH–π and CH–O interactions and the marked CPL amplification in solution.

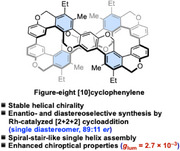

## Introduction

**Figure 1:**
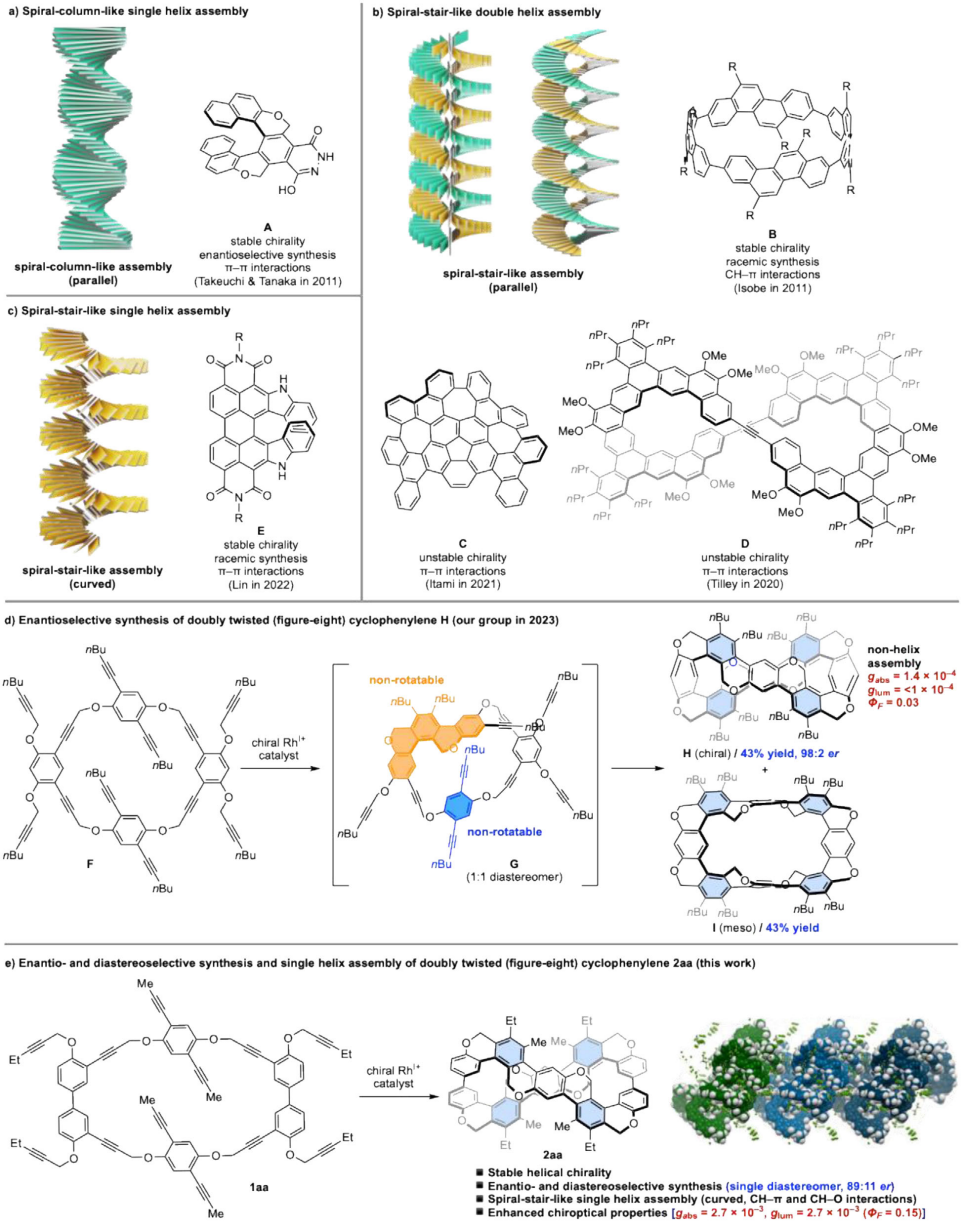
Double‐ and single‐helix assembly of chiral π‐conjugated molecules. The benzene ring formed by the [2 + 2 + 2] cycloaddition is depicted in light blue, while the rear part of the figure‐eight structure is shaded. *er* = enantiomeric ratio.

The helical assembly of chiral molecules enables the transfer of microscopic unimolecular chirality to macroscopic supramolecular chirality,^[^ ^1^, ^2^, ^3^, ^4^, ^5^, ^6^ ^]^ enhancing various chiral properties, such as chiral recognition,^[^ ^7^, ^8^, ^9^, ^10^, ^11^, ^12^ ^]^ asymmetric catalysis,^[^ ^13^, ^14^, ^15^, ^16^, ^17^, ^18^ ^]^ charge transport,^[^ ^19^, ^20^, ^21^ ^]^ and circular polarization.^[^ ^22^, ^23^, ^24^, ^25^, ^26^, ^27^, ^28^ ^]^ Strong noncovalent interactions, such as ionic and hydrogen bonding, are commonly used to precisely program the arrangement of the molecules to construct such assemblies. In chiral aromatic nanocarbons, the helix assembly has also been achieved by weak noncovalent interactions, such as π–π and CH–π interactions,^[^ ^29^, ^30^, ^31^, ^32^, ^33^, ^34^, ^35^, ^36^, ^37^, ^38^, ^39^, ^40^ ^]^ which have attracted much attention for their application in chiral materials. The most common mode is a spiral‐column‐like single helix assembly due to face‐to‐face parallel π–π interactions, of which there are numerous reports.^[^ ^29^, ^30^, ^31^, ^32^, ^33^, ^34^, ^35^, ^36^ ^]^ For example, our research group reported that trimerized helicene‐like molecule **A** with hydrogen bonding forms the spiral‐column‐like single helix assembly through π–π interactions (Figure 1a).^[^ ^33^ ^]^ In addition to this mode, several spiral‐stair‐like helix assemblies with parallel stacking of molecules have been reported in aromatic nanocarbons with multiple chirality‐related irregularities, but examples are few (Figure 1b).^[^ ^37^, ^38^, ^39^, ^40^ ^]^ This form of the helix assembly has internal and external voids, which would have applications in chiral molecular recognition and asymmetric catalysis. Sato et al. reported the helix assembly of planar‐chiral cyclo‐2,8‐chrysenylene **B**, in which an alkyl group protruding outside the ring fits into the inner ring vacancy of a neighboring molecule through CH–π interactions.^[^ ^37^ ^]^ As examples due to π–π interactions, Kato et al. and Kiel et al. reported the helix assemblies of partially deficient warped nanographene **C** ^[^ ^38^ ^]^ and figure‐eight expanded helicene dimer **D**,^[^ ^39^ ^]^ respectively. In these examples (compounds **B**–**D**), the helix pitch is long due to the small overlap between the molecules, forming the double helix to fill the gap between them. In contrast, Lin et al. reported the only example of the spiral‐stair‐like single helix assembly in helically chiral single azahelicene **E** by curving with dense π–π interactions (Figure 1c).^[^ ^40^ ^]^ However, **C** and **D** did not have stable chirality at room temperature, and **B** and **E** required separation of diastereomers and/or enantiomers.

Recently, our research group reported the asymmetric synthesis of figure‐eight^[^ ^41^, ^42^, ^43^, ^44^, ^45^, ^46^, ^47^, ^48^, ^49^, ^50^, ^51^, ^52^, ^53^, ^54^, ^55^, ^56^, ^57^, ^58^, ^59^, ^60^, ^61^, ^62^, ^63^, ^64^, ^65^ ^]^ [8]cyclophenylene **H** ^[^ ^66^ ^]^ by the rhodium‐catalyzed^[^ ^67^, ^68^, ^69^ ^]^ four consecutive intramolecular [2 + 2 + 2] cycloaddition^[^ ^70^, ^71^, ^72^, ^73^, ^74^, ^75^, ^76^, ^77^, ^78^ ^]^ of dodecayne **F** (Figure 1d).^[^ ^79^, ^80^, ^81^, ^82^, ^83^, ^84^, ^85^ ^]^ Although this synthesis achieved high enantioselectivity (*er* = 98:2), diastereoselectivity could not be controlled to form a 1:1 mixture of chiral *P,P* isomer **H** and achiral *P,M* (*meso*) isomer **I**. Once‐cyclized‐intermediate **G** would have two nonrotatable planar chirality (orange and blue units). However, only one planar chirality (orange unit) is controlled by the chiral Rh^I+^ catalyst, which results in a mixture of 1:1 diastereomers. Chiral [8]cyclophenylene **H** is an aromatic nanocarbon with a distinct 3D concavity and convexity due to two stable chirality. Therefore, we expected that **H** exhibits the spiral‐stair‐like helix assembly and good chiroptical properties. However, we failed to grow its chiral crystals and observed poor chiroptical properties [*g*_abs _= 1.4 × 10^−4^, *g*_lum_ = <1 × 10^−4^ (*Φ*_F_ = 0.036)].

Here, we have succeeded in the enantio‐ and diastereoselective synthesis of figure‐eight [10]cyclophenylene **2aa** with stable helical chirality by the rhodium‐catalyzed four consecutive intramolecular [2 + 2 + 2] cycloadditions of dodecayne **1aa**, in which two benzene units are replaced with two biphenyl units, resulting in increased rotational freedom of the reaction intermediates (Figure 1e). Pleasingly, **2aa** shows the spiral‐stair‐like single helix assembly in the crystal due to CH–π and CH–O interactions and a marked improvement in circularly polarized luminescence (CPL) properties in solution compared to the previously reported figure‐eight [8]cyclophenylene **H**. We also report on experimental validation of enantio‐ and diastereo‐determining steps of four consecutive [2 + 2 + 2] cycloadditions.

## Results and Discussion

**Figure 2:**
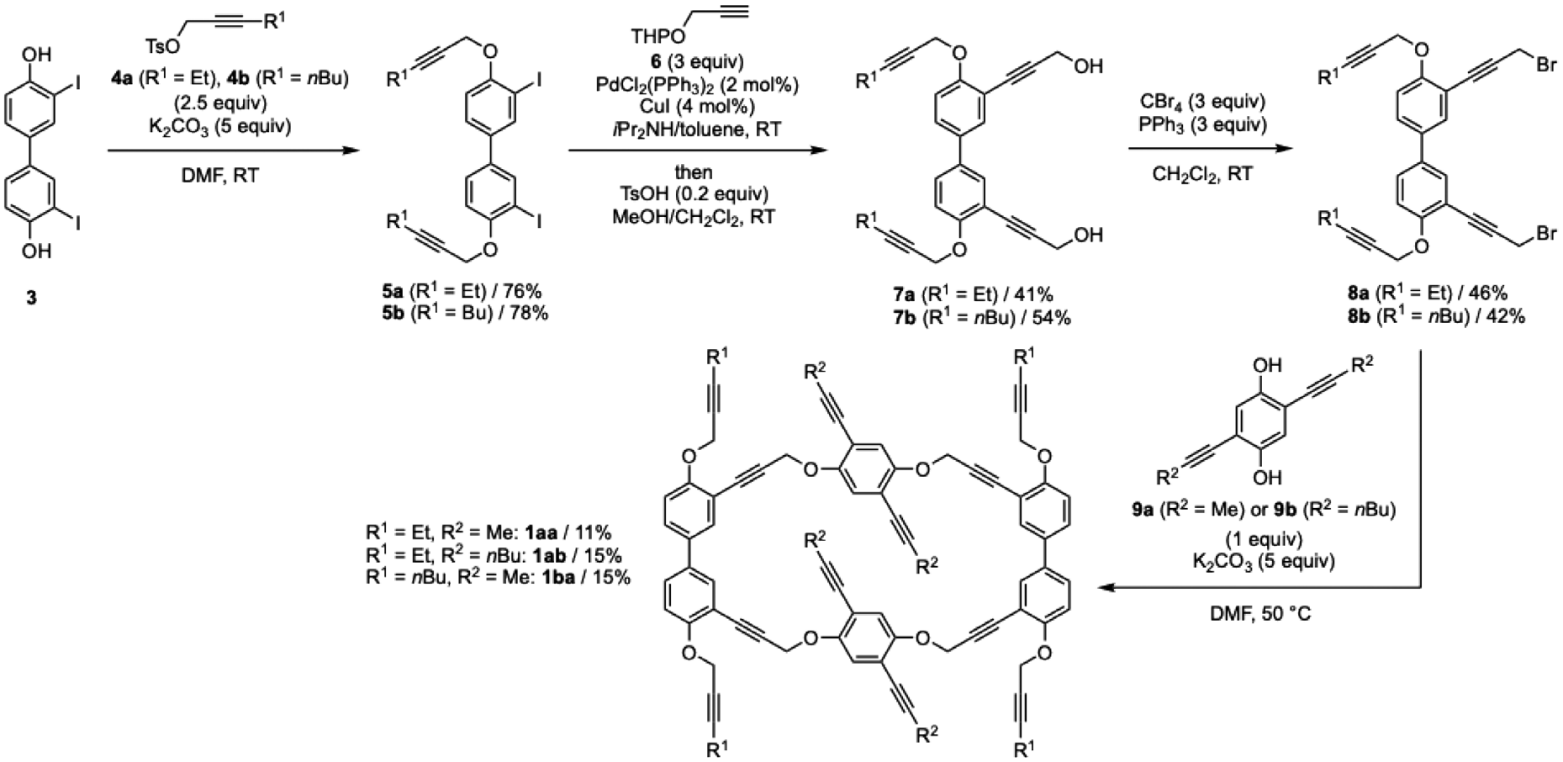
Synthesis of cyclic dodecaynes **1**. Ts = *p*‐toluenesulfonyl. THP = 2‐tetrahydropyranyl.

The synthesis of dodecaynes **1**, the precursors of figure‐eight [10]cyclophenylenes **2**, is shown in Figure 2. Etherification of biphenyldiol **3** ^[^ ^86^ ^]^ with tosylates **4a** ^[^ ^87^ ^]^ and **4b** ^[^ ^88^ ^]^ gave bis‐propargyl ethers **5a** and **5b** in high yields. Sonogashira coupling of terminal alkyne **6** with diiodides **5a** and **5b** followed by removal of the THP protecting group gave tetrayne‐diols **7a** and **7b** in moderate yields. Subsequently, bromination of **7a** and **7b** with CBr_4_ and PPh_3_ gave dibromides **8a** and **8b** in moderate yields. Finally, the macrocyclization reactions of diyne **9a** ^[^ ^89^ ^]^ with tetrayne **8a**, diyne **9a** with tetrayne **8b**, and diyne **9b** ^[^ ^80^ ^]^ with tetrayne **8a** in DMF using potassium carbonate as a base gave cyclic dodecaynes **1aa**, **1ab**, and **1ba** in 11%, 15%, and 15% yields, respectively.

When the synthesized dodecayne **1aa** was subjected to a Rh^I+^/(*S*)‐H_8_‐binap catalyst (80 mol%), four consecutive intramolecular [2 + 2 + 2] cycloadditions proceeded at room temperature to afford figure‐eight [10]cyclophenylene **2aa** with stable helical chirality in moderate yield and enantioselectivity (51% yield and *er* = 76:24) (Table 1, entry 1). Notably, the *meso* form [(*P,M*)‐**2aa**], whose formation was inevitable in our previous report,^[^ ^66^ ^]^ was not observed at all, forming only the chiral form [(*M,M*)‐**2aa**] as a single diastereomer. Screening of axially chiral biarylbisphosphine ligands (entries 1–3) showed that the enantioselectivity tends to improve with decreasing the dihedral angle of the ligand (dihedral angle: H_8_‐binap^[^ ^90^ ^]^ > binap^[^ ^91^ ^]^ > segphos^[^ ^91^ ^]^ enantioselectivity: H_8_‐binap < binap < segphos). The reaction was not completed with segphos, but increasing the temperature to 40 °C completed the reaction, and the yield was increased to 69% while maintaining the enantioselectivity (entry 4). The bulkiness of the aryl group on the phosphorus of the segphos ligand was investigated (entries 4–6), revealing that both yield and enantioselectivity were improved using (*S*)‐tol‐segphos (entry 5). We performed similar ligand screenings for dodecaynes **1ab** (entries 7–10) and **1ba** (entries 11–14) with different substituents. For **1ab**, the corresponding figure‐eight [10]cyclophenylene **2ab** was obtained with the highest *er* using segphos (entry 9), although the yield using binap was the highest (entry 8). For **1ba**, the corresponding figure‐eight [10]cyclophenylenes **2ba** was obtained with the highest enantioselectivity using tol‐segphos (entry 14), while the yield was the lowest. Using segphos afforded **2ba** in the highest yield with good enantioselectivity (entry 13).

**Table 1:** Enantio‐ and diastereoselective synthesis of figure‐eight [10]cyclophenylenes **2**.

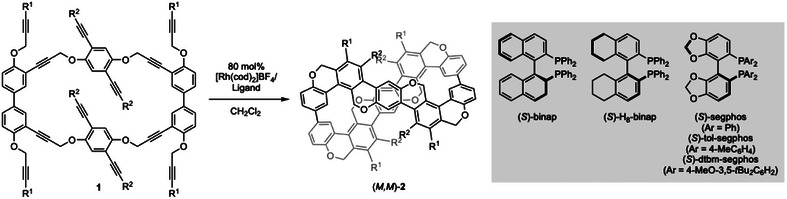					
Entry	1	Ligand	Temp	Time [h]	2 / % Yield^a)^ (*er*)
1	**1aa**	(*S*)‐H_8_‐binap (80°)^b)^	RT	16	(*M,M*)‐(–)‐**2aa** / 51 (76:24)
2	**1aa**	(*S*)‐binap (72°)^c)^	RT	16	(*M,M*)‐(–)‐**2aa** / 47 (80:20)
3	**1aa**	(*S*)‐segphos (67°)^c)^	RT	16	(*M,M*)‐(–)‐**2aa** / 44 (85:15)
4	**1aa**	(*S*)‐segphos	40 °C	16	(*M,M*)‐(–)‐**2aa** / 69 (84:16)
5	**1aa**	(*S*)‐tol‐segphos	40 °C	24	(*M,M*)‐(–)‐**2aa** / 87 (89:11)
6	**1aa**	(*S*)‐dtbm‐segphos	40 °C	16	(*P,P*)‐(+)‐**2aa** / 55 (53:47)
7	**1ab**	(*S*)‐H_8_‐binap	RT	16	(–)‐**2ab** / 76 (52:48)
8	**1ab**	(*S*)‐binap	RT	16	(–)‐**2ab** / 94 (68:32)
9	**1ab**	(*S*)‐segphos	RT	16	(–)‐**2ab** / 89 (79:21)
10	**1ab**	(*S*)‐tol‐segphos	RT	16	(–)‐**2ab** / 60 (64:36)
11	**1ba**	(*S*)‐H_8_‐binap	RT	16	(*M,M*)‐(–)‐**2ba** / 69 (75:25)
12	**1ba**	(*S*)‐binap	RT	16	(*M,M*)‐(–)‐**2ba** / 59 (79:21)
13	**1ba**	(*S*)‐segphos	RT	16	(*M,M*)‐(–)‐**2ba** / 94 (85:15)
14	**1ba**	(*S*)‐tol‐segphos	RT	16	(*M,M*)‐(–)‐**2ba** / 49 (90:10)

^a)^ Isolated yield;

^b)^ A dihedral angle of a Rh^I^ complex, shown in Ref. [90];

^c)^ Dihedral angles of Rh^I^ complexes, shown in Ref. [91]. cod = 1,5‐cyclooctadiene.

**Figure 3:**
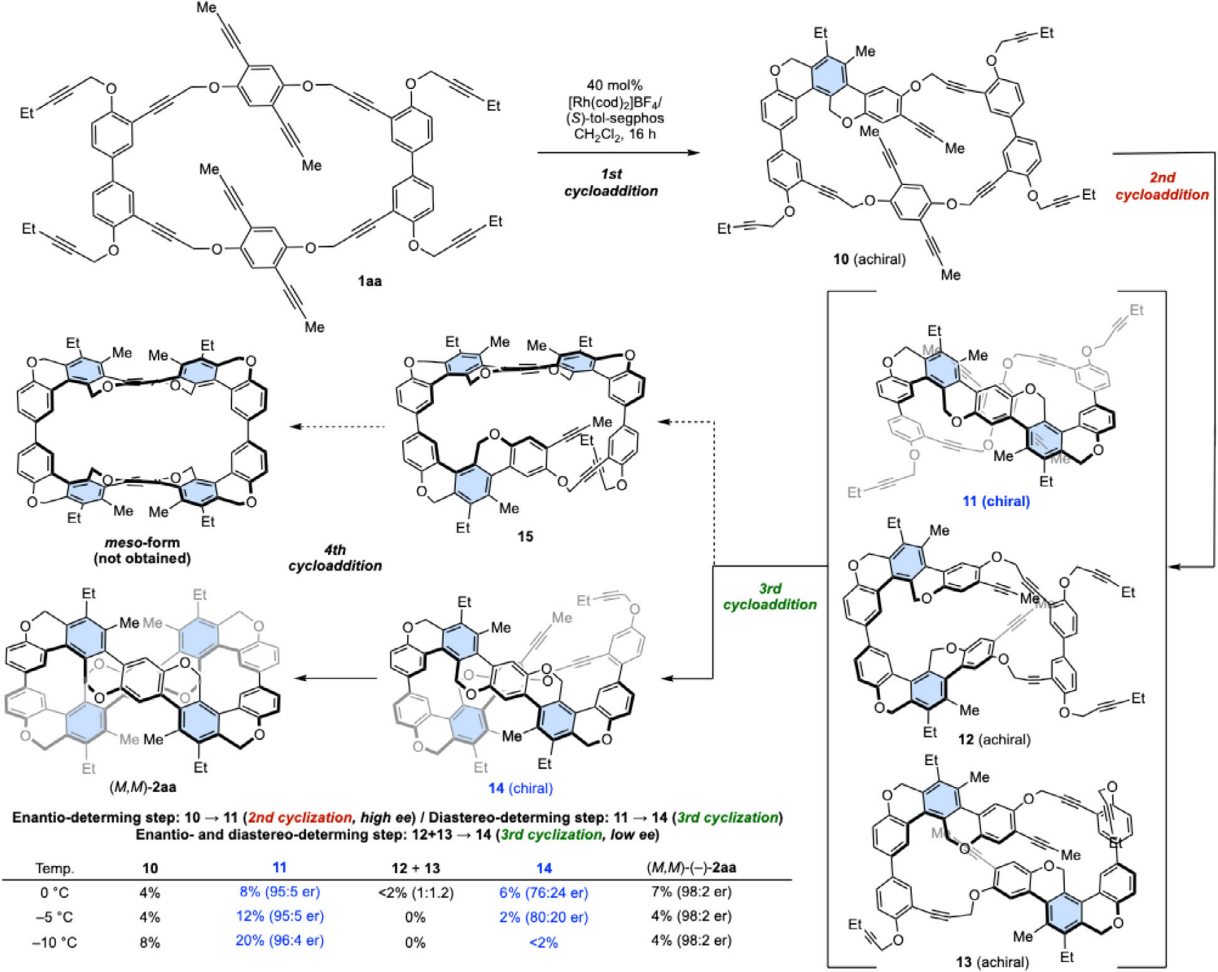
Mechanistic studies on stereoselectivity. The benzene ring formed by the [2 + 2 + 2] cycloaddition is depicted in light blue, while the rear part of the figure‐eight structure is shaded.

To identify reaction intermediates, we examine the reaction of **1aa** at low temperatures (−10 to 0 °C) with reduced amounts of the Rh^I+^/(*S*)‐tol‐segphos catalyst (40 mol%), revealing that once‐cyclized‐intermediate **10**, twice‐cyclized‐intermediates **11**–**13**, and three times‐cyclized‐intermediate **14** were isolated along with the target compound (*M,M*)‐(–)‐**2aa** by repeated silica gel preparative thin layer chromatography (Figure 3). As expected, intermediate **15**, which could give the *meso* compound, was undetectable. To explore the steps that determine the enantioselectivity of the final product (*M,M*)‐(–)‐**2aa**, we examined optical resolution using chiral HPLC and optical rotation measurements for **10**–**14**, revealing that **11** and **14** are chiral compounds, while **10**, **12**, and **13** are achiral compounds. Among *C*_2_‐symmetric twice‐cyclized‐intermediates, we determined the chiral form with the highest rotational barrier to be intermediate **11**, and the others were determined to be **12** and **13**, isolated as an inseparable mixture. The structure of **11** was also confirmed by 2D NMR (NOESY and HMBC). Thus, the 2nd cycloaddition from **10** to **11** is the enantio‐determining step, and the subsequent 3rd cycloaddition from **11** to **14** is the diastereo‐determining step. In addition, the 3rd cycloaddition from **12** and **13** to **14** is the enantio‐ and diastereo‐determining step.

The reaction of **1aa** at 0 °C for 16 h afforded intermediates **10**–**14** along with the fully cyclized product **2aa** (Figure 3). Although intermediate **11** and the fully cyclized product **2aa** were obtained with excellent enantiomeric ratios, the enantiomeric ratio of intermediate **14** was moderate. The reaction of **1aa** at –10 °C for 16 h afforded increased amounts of intermediates **10** and **11** and a decreased amount of **2aa** with excellent enantiomeric ratios for **11** and **2aa** (Figure 3). Importantly, little or no intermediates **12**–**14** were detected. At the intermediate temperature of –5 °C, **11** and **14** were obtained in intermediate yields at –10 and 0 °C. The enantiomeric ratio of **14** was higher than the value at 0 °C. On the basis of these results, the formation of **11** from **10** would be kinetically favorable and proceed with high enantioselectivity, while that of **12** and **13** from **10** would be kinetically unfavorable and proceed with low enantioselectivity. The remaining **14** in a lower enantiomeric ratio than that of **2aa** suggests that the reaction of one enantiomer may be faster than the other in forming **2aa** from **14** at low temperatures (−10 to 0 °C).

**Figure 4:**
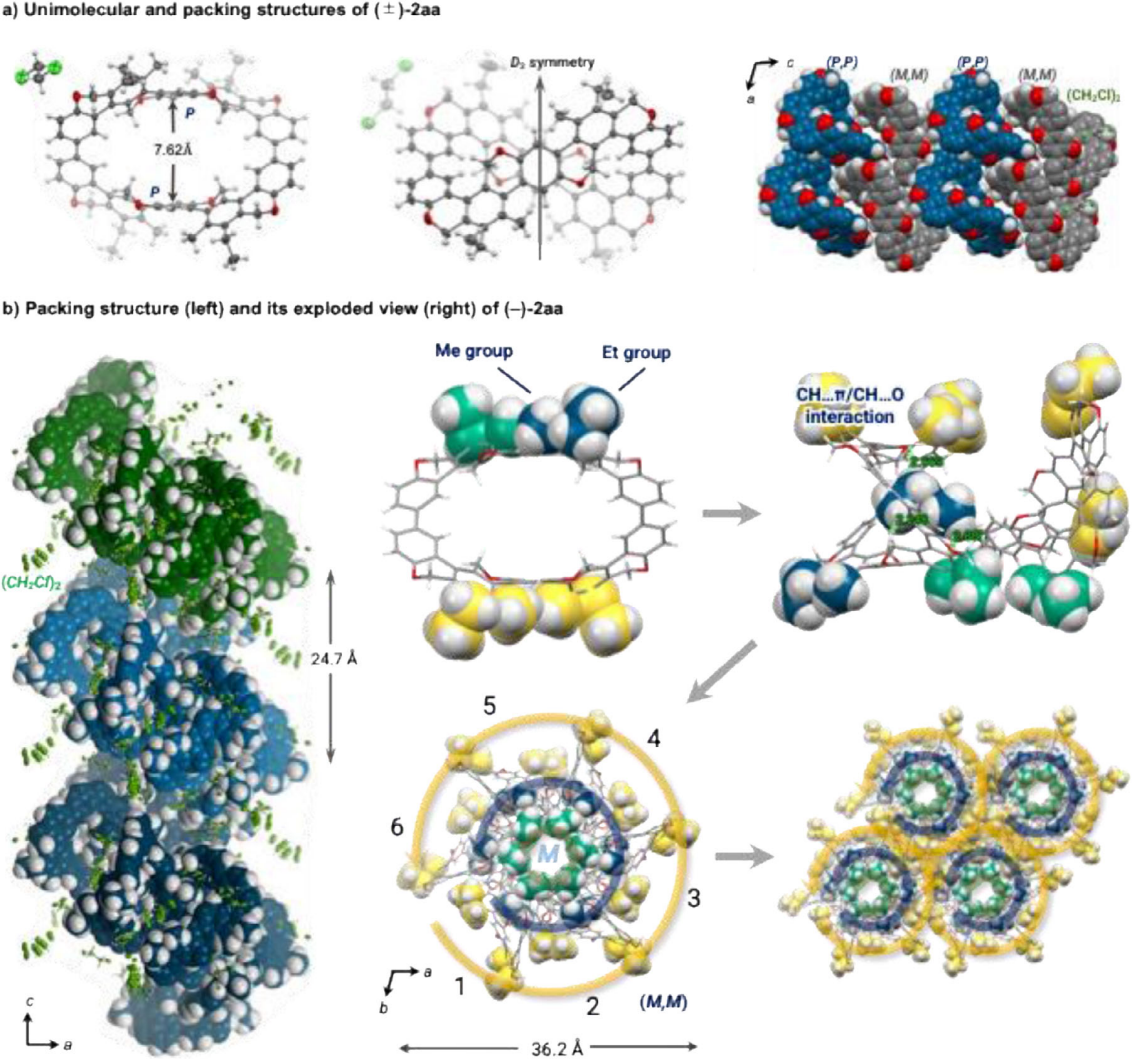
X‐Ray crystal structures of (±)‐**2aa** and (*M,M*)‐(–)‐**2aa**.

We successfully obtained racemic and chiral crystals by vapor diffusion of hexane into a 1,2‐dichloroethane solution of **2aa**. Thus, we performed their single‐crystal X‐ray diffraction analyses and clarified the structures of figure‐eight [10]cyclophenylenes **2** in the solid state (Figure 4).^[^ ^92^ ^]^ The racemic crystal confirmed that **2aa** has an elliptical *D*_2_ symmetric structure (Figure 4a). The distance between the benzene rings in the closest writhe moiety is 7.62 Å. The vacancies inside the ring are slightly wider than those in the figure‐eight [8]cyclophenylene **H**. The crystal lattice contains both *M,M*‐ and *P,P*‐isomers in a 1:1 ratio, and the stacking structure consists of alternating columns of *P*‐ and *M*‐isomers. On the other hand, the chiral crystal of (*M,M*)‐(–)‐**2aa** has a hexagonal space group *P*6_5_ with a spiral‐stair‐like single helix assembly (Figure 4b, left). The helix consists of six molecules in a single winding with a diameter of 36.2 Å (Figure 4b, right). This single unit is stacked in the *c*‐axis direction to form the single helix, and dichloroethane is incorporated into the 24.7 Å pitch between the layers (Figure 4b, left). Notably, the *M,M* isomer forms a counterclockwise chiral single helix, reflecting the twist chirality of the single molecule into the higher‐order helical chirality.

To elucidate the mechanism of helix formation, we investigated the contribution of the 3D unevenness of the molecule and the alkyl (ethyl and methyl) side chains (Figure 4b, right; See the Supporting Information for the movie on helix formation). Each side chain (blue, green, and yellow) of (*M,M*)‐(–)‐**2aa** is in a different region in the helix, and each plays a distinct role in the helix formation. The blue ethyl side chain, which plays the decisive role, fits into the expanding flexible vacancy of the neighboring molecule and engages like a tenon and mortise joint. Numerous CH…π and CH…O interactions are observed between the π‐skeleton and between the π‐skeleton and side chains, resulting in a rigid molecular stack. Repeating such stacks with the green side chain inside and the yellow side chain on the outside constructs a single helical structure. The yellow side chains extending outward allow interactions between the helices, forming many CH…π interactions between the adjacent columns. Of the multiple side chains, only the blue ethyl side chain always fits into the vacancy of the neighboring molecule, which may be essential in building the chiral helical structure. Indeed, **2ab**, where the methyl group of **2aa** is replaced by a butyl group, showed poor crystallinity. In (*M,M*)‐(–)‐**2ba**, where the ethyl group of (*M,M*)‐(–)‐**2aa** is replaced by a butyl group, the butyl side chain could not fit into the vacancy of the neighboring molecule. Thus, the (*M,M*)‐**2ba** shifted clockwise (opposite to molecular chirality) and stacked in a tube shape (space group *P*2_1_2_1_2, Figure S22).

We further compared the strain energies of figure‐eight [10]cyclophenylene **2** and figure‐eight [8]cyclophenylene **H** by DFT calculations. The strain energies of simplified **2‐Me_8_**, replacing all alkyl groups by the methyl groups, and **H**, based on the homodesmotic reaction method,^[^ ^93^ ^]^ are 20.6 and 35.9 kcal mol^−1^, respectively, indicating that the strain is greatly reduced in **2‐Me_8_** than **H** (Figure S23).

**Figure 5:**
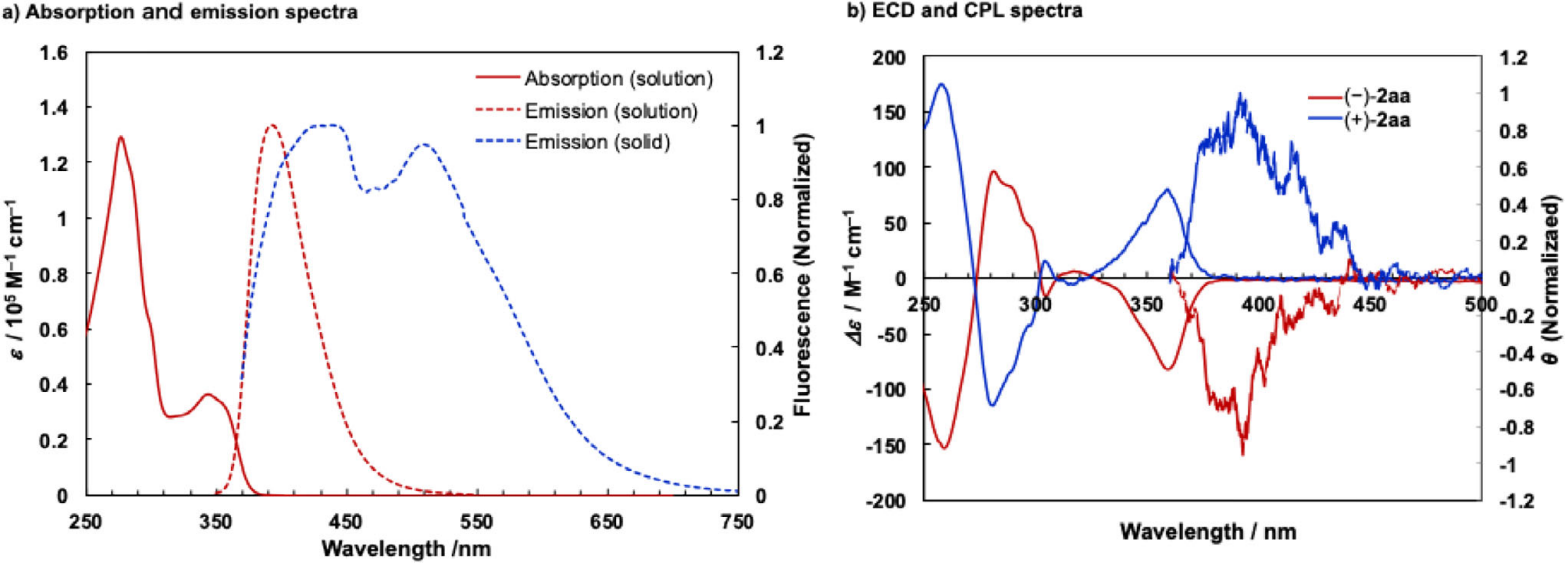
Photophysical and chiroptical properties of 2aa.

Photophysical and chiroptical properties of figure‐eight [10]cyclophenylene **2aa** in solution are shown in Figure 5, summarizing the data in Table 2. For absorption and emission spectra of **2aa** in dichloromethane solution (Figure 5a), absorption maxima were observed at 277 and 344 nm, and a single fluorescence maximum was observed at 393 nm, which are redshifted compared to those of **H**. The HOMO/LUMO energy gap of **2‐Me_8_** (3.93 eV) with a larger diameter is smaller than that of **H** (4.09 eV, Figure S24), consistent with this redshift of absorption maxima. The fluorescence quantum yield of [10]cyclophenylene **2aa** is 15%, showing a marked improvement over [8]cyclophenylene **H** (3%).^[^ ^66^ ^]^ **2aa** also exhibited fluorescence in the solid state (Figure 5a). Like the solid‐state absorption spectrum (Figure S11), a broad fluorescence spectrum was observed, featuring two prominent peaks (Figure S13), which may result from excimer formation due to helix assembly. Additionally, the fluorescence quantum yield in the solid state was similar to that observed in the solution state.

**Table 2:** Photophysical and chiroptical properties of figure‐eight cyclophenylenes **H** and **2aa**.^a)^

Compound	Absorption *λ*_max_ [nm]	Fluorescence *λ*_max_ [nm] (Excitation Wavelength, nm)	*Φ*_F_ (Excitation Wavelength, nm)	[α]^25^_D_	*g*_abs_ (Wavelength, nm)	*g*_lum_ (Wavelength, nm)
(–)‐**2aa**	344, 277 285^b)^	393 (370) 439, 510 (330)^b)^	0.153 (370) 0.133 (370)^b)^	−1698° (89:11 *er*)	2.7 x 10^−3^ (359) 7.9 x 10^−3^ (367)^b)^	2.7 x 10^−3^ (380) –^b)^
(–)‐**H** ^c)^	326, 280	382 (330)	0.036 (330)	−775° (98:2 *er*)	1.4 x 10^−4^ (345)	<1 x 10^−4^ ^d)^

^a)^ 1.0 × 10^−5^ M in CH_2_Cl_2_ at 25 °C;

^b)^ In the solid state;

^c)^ Data of ref 12;

^d)^ Below detection limit.

We then measured the electronic circular dichroism (ECD) spectra for **2aa** to evaluate the chiroptical properties (Figure 5b). An explicit cotton effect based on stable twisted chirality was observed, and the sign of the cotton effect in the measured spectrum of (–)‐**2aa** is consistent with that of (*M,M*)‐**2aa** simulated by the TDDFT calculation. This result suggests that the absolute configuration of (–)‐**2aa** obtained when using (*S*)‐segphos is determined to be the (*M,M*)‐isomer, which is consistent with the X‐ray crystallographic analysis. The anisotropy factor of the ECD (*g*_abs_ = 2.7 × 10^−3^ at 359 nm) is markedly larger (about 20‐fold) than that of **H** (*g*_abs_ = 1.4 × 10^−4^).^[^ ^66^ ^]^ We also measured the CPL spectra for **2aa** (Figure 5c). As with the ECD spectra, the anisotropy factor of the CPL (*g*_lum_ = 2.7 × 10^−3^ at 380 nm) is markedly larger than that of **H** (*g*_lum_ = <1 × 10^−4^).^[^ ^66^ ^]^ We measured the solid‐state ECD and CPL using chiral crystals of **2aa**. The anisotropy factor of the ECD in the solid state is larger (*g*_abs_ = 7.9 × 10^−3^ at 367 nm, Figure S15) compared to the solution state (*g*_abs_ = 2.7 × 10^−3^). However, we did not observe any solid‐state CPL (Figure S17). We believe the absence of the solid‐state CPL may result from the helical axes and transition dipole moments of individual molecules in the spiral‐stair‐like helix assembly being mutually orthogonal in the excited state, which causes them to cancel each other out.

## Conclusion

We have achieved the enantio‐ and diastereoselective synthesis of helically chiral figure‐eight [10]cyclophenylenes **2** by the cationic rhodium(I)/biaryl bisphosphine complex‐catalyzed four consecutive intramolecular [2 + 2 + 2] cycloadditions of dodecaynes **1**. We demonstrate enantio‐ and diastereo‐determining steps by isolating five chiral and achiral reaction intermediates (**11**–**15**) in the reactions of dodecayne **1aa** at low temperatures with reduced amounts of the rhodium(I) catalyst. Introducing two flexible biphenyl units into **1** increases the rotational freedom of the reaction intermediates, yielding products **2** as a single diastereomer. The chiral figure‐eight [10]cyclophenylene **2aa** with ethyl and methyl side chains exhibits a spiral‐stair‐like single helix assembly in the crystal due to CH–π and CH–O interactions and a marked improvement in circularly polarized luminescence (CPL) properties [*g*_lum _= 2.7 × 10^−3^ (*Φ*_F_ = 0.153)] in solution, compared to the analogous helically chiral figure‐eight [8]cyclophenylene **H** [*g*_lum_ = <1 × 10^−4^ (*Φ*_F_ = 0.036)]. In the solid state, **2aa** displayed a broad fluorescence spectrum, likely due to excimer formation resulting from helix assembly. The anisotropy factor of solid‐state ECD is greater than that of the solution state. However, no solid‐state CPL was observed. The absence of CPL may stem from the fact that, unlike in a spiral‐column‐like helix assembly, the helical axis and the transition dipole moments of the individual molecules in a spiral‐stair‐like helix assembly are oriented orthogonally to each other, leading to their cancellation. Although flexible figure‐eight structures are generally less effective than rigid ones for enhancing CPL in the solid state, we have successfully transferred microscopic unimolecular chirality to macroscopic supramolecular chirality by employing a spiral‐stair‐like helix assembly using these flexible figure‐eight molecules. Future research will explore the unique chiral properties beyond CPL derived from this spiral‐stair‐like helix assembly.

## Conflict of Interests

The authors declare no conflict of interest.

## Supporting information



Supporting Information

Supporting Information

Supporting Information

## Data Availability

The data that support the findings of this study are available in the supplementary material of this article.

## References

[1] S. Jia , T. Tao , Y. Xie , L. Yu , X. Kang , Y. Zhang , W. Tang , J. Gong , Small 2024, 20, e2307874. 37890278 10.1002/smll.202307874

[2] F. García , R. Gómez , L. Sánchez , Chem. Soc. Rev. 2023, 52, 7524–7548. 37819705 10.1039/d3cs00470h

[3] Y. Dorca , E. E. Greciano , J. S. Valera , R. Gómez , L. Sánchez , Chem.‐Eur. J. 2019, 25, 5848–5864. 30561853 10.1002/chem.201805577

[4] S. M. Morrow , A. J. Bissette , S. P. Fletcher , Nat. Nanotechnol. 2017, 12, 410–419. 28474691 10.1038/nnano.2017.62

[5] E. Yashima , N. Ousaka , D. Taura , K. Shimomura , T. Ikai , K. Maeda , Chem. Rev. 2016, 116, 13752–13990. 27754649 10.1021/acs.chemrev.6b00354

[6] M. Liu , L. Zhang , T. Wang , Chem. Rev. 2015, 115, 7304–7397. 26189453 10.1021/cr500671p

[7] M. Oka , R. Kozako , Y. Teranishi , Y. Yamada , K. Miyake , T. Fujimura , R. Sasai , T. Ikeue , H. Iida , Chem.‐Eur. J. 2024, 30, e202303353. 38012829 10.1002/chem.202303353

[8] K. Salikolimi , V. K. Praveen , A. A. Sudhakar , K. Yamada , N. N. Horimoto , Y. Ishida , Nat. Commun. 2020, 11, 2311. 32385267 10.1038/s41467-020-16127-6 PMC7210886

[9] S. Li , L. Zhang , J. Jiang , Y. Meng , M. Liu , ACS Appl. Mater. Interfaces 2017, 9, 37386–37394. 28972781 10.1021/acsami.7b10353

[10] L. Zhang , Q. Jin , K. Lv , L. Qin , M. Liu , Chem. Commun. 2015, 51, 4234–4236. 10.1039/c5cc00261c 25670484

[11] H. Qiu , Y. Inoue , S. Che , Angew. Chem. Int. Ed. 2009, 48, 3069–3072; 10.1002/anie.200900303 19309027

[12] K. Kodama , Y. Kobayashi , K. Saigo , Chem.‐Eur. J. 2007, 13, 2144–2152. 17154322 10.1002/chem.200601295

[13] D. Li , C. Gao , C. Zhao , Q. Sun , Z. Xi , J. Han , R. Guo , Chem. Commun. 2024, 60, 4569–4572. 10.1039/d4cc00637b 38572692

[14] C. Gao , S. Li , C. Zhao , Q. Sun , X. Sun , L. Ge , L. Wang , Z. Xi , J. Han , R. Guo , Small 2024, 20, 2310234. 10.1002/smll.202310234 38155520

[15] M. Tena‐Solsona , J. Nanda , S. Díaz‐Oltra , A. Chotera , G. Ashkenasy , B. Escuder , Chem.‐Eur. J. 2016, 22, 6687–6694. 27004623 10.1002/chem.201600344

[16] J. Jiang , Y. Meng , L. Zhang , M. Liu , J. Am. Chem. Soc. 2016, 138, 15629–15635. 27934018 10.1021/jacs.6b08808

[17] A. Desmarchelier , X. Caumes , M. Raynal , A. Vidal‐Ferran , P. W. N. M. v an Leeuwen , L. Bouteiller , J. Am. Chem. Soc. 2016, 138, 4908–4916. 26998637 10.1021/jacs.6b01306

[18] Q. Jin , L. Zhang , H. Cao , T. Wang , X. Zhu , J. Jiang , M. Liu , Langmuir 2011, 27, 13847–13853. 21978005 10.1021/la203110z

[19] Y. Zhang , P. Chen , L. Jiang , W. Hu , M. Liu , J. Am. Chem. Soc. 2009, 131, 2756–2757. 19206235 10.1021/ja805891k

[20] Y. Yamamoto , T. Fukushima , Y. Suna , N. Ishii , A. Saeki , S. Seki , S. Tagawa , M. Taniguchi , T. Kawai , T. Aida , Science 2006, 314, 1761–1764. 17170300 10.1126/science.1134441

[21] J. P. Hill , W. Jin , A. Kosaka , T. Fukushima , H. Ichihara , T. Shimomura , K. Ito , T. Hashizume , N. Ishii , T. Aida , Science 2004, 304, 1481–1483. 15178796 10.1126/science.1097789

[22] Y. Wang , D. Niu , G. Ouyang , M. Liu , Nat. Commun. 2022, 13, 1370. 35361805 10.1038/s41467-022-29396-0 PMC8971395

[23] S. Hu , L. Hu , X. Zhu , Y. Wang , M. Liu , Angew. Chem. Int. Ed. 2021, 60, 19451–19457; 10.1002/anie.202107842 34196488

[24] D. Yang , P. Duan , L. Zhang , M. Liu , Nat. Commun. 2017, 8, 15727. 28585538 10.1038/ncomms15727 PMC5467208

[25] J. Kumar , T. Nakashima , H. Tsumatori , T. Kawai , J. Phys. Chem. Lett. 2014, 5, 316–321. 26270706 10.1021/jz402615n

[26] J. Kumar , T. Nakashima , H. Tsumatori , M. Mori , M. Naito , T. Kawai , Chem.‐Eur. J. 2013, 19, 14090–14097. 24026812 10.1002/chem.201302146

[27] J. Liu , H. Su , L. Meng , Y. Zhao , C. Deng , J. C. Y. Ng , P. Lu , M. Faisal , J. W. Y. Lam , X. Huang , H. Wu , K. S. Wong , B. Z. Tang , Chem. Sci. 2012, 3, 2737–2747.

[28] K. E. Phillips , T. J. Katz , S. Jockusch , A. J. Lovinger , N. J. Turro , J. Am. Chem. Soc. 2001, 123, 11899–11907. 11724596 10.1021/ja011706b

[29] M. S. Sundar , B. Klepetářová , L. Bednárová , G. Muller , Eur. J. Org. Chem. 2021, 2021, 146–150.

[30] N. J. Schuster , D. W. Paley , S. Jockusch , F. Ng , M. L. Steigerwald , C. Nuckolls , Angew. Chem. Int. Ed. 2016, 55, 13519–13523; 10.1002/anie.201607878 27717214

[31] K. Nakano , H. Oyama , Y. Nishimura , S. Nakasako , K. Nozaki , Angew. Chem. Int. Ed. 2012, 51, 695–699; 10.1002/anie.201106157 22135206

[32] T. Hatakeyama , S. Hashimoto , T. Oba , M. Nakamura , J. Am. Chem. Soc. 2012, 134, 19600–19603. 23167918 10.1021/ja310372f

[33] T. Kaseyama , S. Furumi , X. Zhang , K. Tanaka , M. Takeuchi , Angew. Chem. Int. Ed. 2011, 50, 3684–3687; 10.1002/anie.201007849 21416571

[34] M. A. Shcherbina , X. B. Zeng , T. Tadjiev , G. Ungar , S. H. Eichhorn , K. E. S. Phillips , T. J. Katz , Angew. Chem. Int. Ed. 2009, 48, 7837–7840; 10.1002/anie.200903658 19739171

[35] K. Sato , S. Arai , T. Yamagishi , T. Tanase , Acta Crystallogr. Sect. C Cryst. Struct. Commun. 2003, 59, 162–164. 10.1107/s0108270102023739 12711796

[36] C. Nuckolls , T. J. Katz , L. Castellanos , J. Am. Chem. Soc. 1996, 118, 3767–3768.

[37] S. Sato , A. Yoshii , S. Takahashi , S. Furumi , M. Takeuchi , H. Isobe , Proc. Natl. Acad. Sci. USA 2017, 114, 13097–13101. 29180419 10.1073/pnas.1717524114 PMC5740620

[38] K. Kato , K. Takaba , S. Maki‐Yonekura , N. Mitoma , Y. Nakanishi , T. Nishihara , T. Hatakeyama , T. Kawada , Y. Hijikata , J. Pirillo , L. T. Scott , K. Yonekura , Y. Segawa , K. Itami , J. Am. Chem. Soc. 2021, 143, 5465–5469. 33759524 10.1021/jacs.1c00863

[39] G. R. Kiel , K. L. Bay , A. E. Samkian , N. J. Schuster , J. B. Lin , R. C. Handford , C. Nuckolls , K. N. Houk , T. D. Tilley , J. Am. Chem. Soc. 2020, 142, 11084–11091. 32450694 10.1021/jacs.0c03177

[40] L. Zhang , S. Chen , J. Jiang , X. Dong , Y. Cai , H.‐J. Zhang , J. Lin , Y.‐B. Jiang , Org. Lett. 2022, 24, 3179–3183. 35475653 10.1021/acs.orglett.2c00928

[41] Q. Zhou , W. Yuan , Y. Li , Y. Han , L. Bao , W. Fan , L. Jiao , Y. Zhao , Y. Ni , Y. Zou , H. Yang , J. Wu , Angew. Chem. Int. Ed. 2024,137, e202417749; 10.1002/anie.202417749 39431291

[42] R. Yoshina , J. Hirano , E. Nishimoto , Y. Sakamoto , S. Minabe , M. Uyanik , K. Ishihara , T. Ikai , E. Yashima , T. Omine , F. Ishiwari , A. Saeki , J. Kim , J. Oh , D. Kim , G. Liu , T. Yasuda , H. Shinokubo , J. Am. Chem. Soc. 2024, 146, 29383–29390. 39315432 10.1021/jacs.4c07985 PMC11528406

[43] L. H. Wang , J. Nogami , Y. Nagashima , K. Tanaka , Org. Lett. 2023, 25, 4225–4230. 37219051 10.1021/acs.orglett.3c00895

[44] Y. Zhang , D. Yang , S. H. Pun , H. Chen , Q. Miao , Precis. Chem. 2023, 1, 107–111. 10.1021/prechem.3c00009 PMC12382250 40881112

[45] L. Palomo , L. Favereau , K. Senthilkumar , M. Stępień , J. Casado , F. J. Ramírez , Angew. Chem. Int. Ed. 2022, e202206976; 10.1002/anie.202206976 PMC9544083 35785514

[46] C. Yao , B. Kauffmann , I. Huc , Y. Ferrand , Chem. Commun. 2022, 58, 5789–5792. 10.1039/d2cc01696f 35466334

[47] M. Krzeszewski , H. Ito , K. Itami , J. Am. Chem. Soc. 2022, 144, 862–871. 34910487 10.1021/jacs.1c10807

[48] X. Zhang , H. Liu , G. Zhuang , S. Yang , P. Du , Nat. Commun. 2022, 13, 3543. 35729154 10.1038/s41467-022-31281-9 PMC9213505

[49] X. Zhang , H. Shi , G. Zhuang , S. Wang , J. Wang , S. Yang , X. Shao , P. Du , Angew. Chem. Int. Ed. 2021, 60, 17368–17372; 10.1002/anie.202104669 33945657

[50] Y. Yang , O. Blacque , S. Sato , M. Juríček , Angew. Chem. Int. Ed. 2021, 60, 13529–13535; 10.1002/anie.202101792 PMC8252656 33635576

[51] J. Wang , Y. Y. Ju , K. H. Low , Y. Z. Tan , J. Liu , Angew. Chem. Int. Ed. 2021, 60, 11814–11818; 10.1002/anie.202102637 33751785

[52] K. Li , Z. Xu , J. Xu , T. Weng , X. Chen , S. Sato , J. Wu , Z. Sun , J. Am. Chem. Soc. 2021, 143, 20419–20430. 34817177 10.1021/jacs.1c10170

[53] W. Fan , T. Matsuno , Y. Han , X. Wang , Q. Zhou , H. Isobe , J. Wu , J. Am. Chem. Soc. 2021, 143, 15924–15929. 34550688 10.1021/jacs.1c08468

[54] L. H. Wang , N. Hayase , H. Sugiyama , J. Nogami , H. Uekusa , K. Tanaka , Angew. Chem. Int. Ed. 2020, 59, 17951–17957; 10.1002/anie.202006959 32618087

[55] T. A. Schaub , E. A. Prantl , J. Kohn , M. Bursch , C. R. Marshall , E. J. Leonhardt , T. C. Lovell , L. N. Zakharov , C. K. Brozek , S. R. Waldvogel , S. Grimme , R. Jasti , J. Am. Chem. Soc. 2020, 142, 8763–8775. 32279489 10.1021/jacs.0c01117

[56] H. Kubo , D. Shimizu , T. Hirose , K. Matsuda , Org. Lett. 2020, 22, 9276–9281. 33213148 10.1021/acs.orglett.0c03506

[57] Y. Nojima , M. Hasegawa , N. Hara , Y. Imai , Y. Mazaki , Chem. Commun. 2019, 55, 2749–2752. 10.1039/c8cc08929a 30633278

[58] A. Robert , G. Naulet , H. Bock , N. Vanthuyne , M. Jean , M. Giorgi , Y. Carissan , C. Aroulanda , A. Scalabre , E. Pouget , F. Durola , Y. Coquerel , Chem.‐Eur. J. 2019, 25, 14364–14369. 31397923 10.1002/chem.201902637

[59] K. Senthilkumar , M. Kondratowicz , T. Lis , P. J. Chmielewski , J. Cybińska , J. L. Zafra , J. Casado , T. Vives , J. Crassous , L. Favereau , M. Stępień , J. Am. Chem. Soc. 2019, 141, 7421–7427. 30998349 10.1021/jacs.9b01797

[60] A. Robert , P. Dechambenoit , E. A. Hillard , H. Bock , F. Durola , Chem. Commun. 2017, 53, 11540–11543. 10.1039/c7cc06798d 28948988

[61] W. Nakanishi , T. Matsuno , J. Ichikawa , H. Isobe , Angew. Chem. Int. Ed. 2011, 50, 6048–6051; 10.1002/anie.201102210 21604350

[62] Y. Zhen , W. Yue , Y. Li , W. Jiang , S. Di Motta , E. Di Donato , F. Negri , S. Ye , Z. Wang , Chem. Commun. 2010, 46, 6078–6080. 10.1039/c0cc01011a 20657895

[63] T. V. V. Ramakrishna , P. R. Sharp , Organometallics 2004, 23, 3079–3081.

[64] B. Thulin , O. Wennerström , Tetrahedron Lett. 1977, 18, 929–930.

[65] B. Thulin , O. Wennerström , B. J. Nielsen , I. Johnson , A. Taticchi , T. Anthonsen , Acta Chem. Scand. B 1976, 30b, 688–690.

[66] J. Nogami , D. Hashizume , Y. Nagashima , K. Miyamoto , M. Uchiyama , K. Tanaka , Nat. Synth. 2023, 2, 888–897.

[67] A. Pla‐quintana , A. Roglans , Molecules 2022, 27, 1332–1360. 35209119 10.3390/molecules27041332 PMC8880486

[68] Y. Shibata , K. Tanaka , in Rhodium Catalysis in Organic Synthesis: Methods and Reactions (Ed.: K. Tanaka ), Wiley‐VCH, Weinheim 2019, pp. 183–228.

[69] T. Shibata , M. Fujimoto , H. Hirashima , T. Chiba , K. Endo , Synthesis 2012, 44, 3269–3284.

[70] P. Matton , S. Huvelle , M. Haddad , P. Phansavath , V. Ratovelomanana‐Vidal , Synthesis 2022, 54, 4–32.

[71] I. G. Stará , I. Starý , Acc. Chem. Res. 2020, 53, 144–158. 31833763 10.1021/acs.accounts.9b00364

[72] A. Pla‐Quintana , A. Roglans , Asian J. Org. Chem. 2018, 7, 1706–1718.

[73] M. Babazadeh , S. Soleimani‐Amiri , E. Vessally , A. Hosseinian , L. Edjlali , RSC Adv. 2017, 7, 43716–43736.

[74] M. Amatore , C. Aubert , Eur. J. Org. Chem. 2015, 265–286.

[75] Transition‐Metal‐Mediated Aromatic Ring Construction (Ed.: K. Tanaka ), Wiley, Hoboken 2013, pp. 243–298.

[76] S. Perreault , T. Rovis , Chem. Soc. Rev. 2009, 38, 3149–3159. 19847348 10.1039/b816702h PMC2893402

[77] K. Tanaka , Chem. Asian J. 2009, 4, 508–518. 19101940 10.1002/asia.200800378

[78] T. Shibata , K. Tsuchikama , Org. Biomol. Chem. 2008, 6, 1317–1323. 18385836 10.1039/b720031e

[79] For a review on cycloparaphenylene synthesis via [2 + 2 + 2] cycloaddition, see: D. Kohrs , J. Volkmann , H. A. Wegner , Chem. Commun. 2022, 58, 7483–7494. 10.1039/d2cc02289c 35748854

[80] Y. Kawai , T. Oriki , Y. Sato , J. Nogami , Y. Kamiya , S. Suzuki , K. Tanaka , Org. Lett. 2024, 26, 7869–7874. 39255331 10.1021/acs.orglett.4c02712 PMC11421083

[81] J. Nogami , Y. Nagashima , H. Sugiyama , K. Miyamoto , Y. Tanaka , H. Uekusa , A. Muranaka , M. Uchiyama , K. Tanaka , Angew. Chem. Int. Ed. 2022, 61, e202200800; 10.1002/anie.202200800 35166005

[82] J. Nogami , Y. Nagashima , K. Miyamoto , A. Muranaka , M. Uchiyama , K. Tanaka , Chem. Sci. 2021, 12, 7858–7865. 34168839 10.1039/d1sc00861g PMC8188516

[83] J. Nogami , Y. Tanaka , H. Sugiyama , H. Uekusa , A. Muranaka , M. Uchiyama , K. Tanaka , J. Am. Chem. Soc. 2020, 142, 9834–9842. 32362122 10.1021/jacs.0c03684

[84] S. Nishigaki , Y. Shibata , A. Nakajima , H. Okajima , Y. Masumoto , T. Osawa , A. Muranaka , H. Sugiyama , A. Horikawa , H. Uekusa , H. Koshino , M. Uchiyama , A. Sakamoto , K. Tanaka , J. Am. Chem. Soc. 2019, 141, 14955–14960. 31418559 10.1021/jacs.9b06197

[85] S. Hitosugi , W. Nakanishi , T. Yamasaki , H. Isobe , Nat. Commun. 2011, 2, 8–12.

[86] A. Kar , N. Mangu , H. M. Kaiser , M. Beller , M. K. Tse , Chem. Commun. 2008, 386, 386–388. 10.1039/b714928j 18399216

[87] M. Zhang , A. A. Sayyad , A. Dhesi , A. Orellana , J. Org. Chem. 2020, 85, 13621–13629. 32954732 10.1021/acs.joc.0c01770

[88] T. Kippo , T. Fukuyama , I. Ryu , Org. Lett. 2011, 13, 3864–3867. 21699262 10.1021/ol201395p

[89] C. Mitsui , H. Tanaka , H. Tsuji , E. Nakamura , Chem. Asian J. 2011, 6, 2296–2300. 21748855 10.1002/asia.201100326

[90] X. Zhang , K. Mashima , K. Koyano , N. Sayo , H. Kumobayashi , S. Akutagawa , H. Takaya , J. Chem. Soc., Perkin. Trans. 1994, 1, 2309.

[91] A. Meißner , A. Preetz , H. J. Drexler , W. Baumann , A. Spannenberg , A. König , D. Heller , ChemPlusChem 2015, 80, 169–180.

[92] CCDC 2390406 [(±)‐**2aa**], CCDC 2388999 [(–)‐**2aa**], and CCDC 2389753 [(–)‐**2ba**] contain the supplementary crystallographic data for this paper. This data can be obtained free of charge via www.ccdc.cam.ac.uk/conts/retrieving.html (or from the Cambridge Crystallographic Data Centre, 12, Union Road, Cambridge CB2 1EZ, UK; fax: (+44) 1223‐336‐033; or deposit@ccdc.cam.ac.uk).

[93] Y. Segawa , A. Yagi , H. Ito , K. Itami , Org. Lett. 2016, 18, 1430–1433. 26953497 10.1021/acs.orglett.6b00365

