# Supplementary material for: Enantio‐ and Diastereoselective Synthesis and Spiral‐Stair‐Like Single Helix Assembly of Figure‐Eight Cyclophenylenes
Source: Angew Chem Int Ed Engl. 2025 Apr 7;64(22):e202502764. doi: 10.1002/anie.202502764 (PMC12105691; doi:10.1002/anie.202502764)
Supplement: Supplementary file 1 — Supporting Information [file ANIE-64-e202502764-s001.pdf]

Table of Contents

1. General Information S2
2. Synthesis of Cyclic Polyynes S3–S7
3. Synthesis of Figure-Eight [10]Cyclophenylenes S8–S9
4. Isolation of Reaction Intermediates S10–S11
5. ^{1}H, ^{13}C, and 2D NMR Spectra of New Compounds S13–S38
6. Chiral HPLC Charts S39–S43
7. Photophysical and Chiroptical Properties S44– S47
8. X-Ray Crystallographic Analyses S48–S58
9. Theoretical Calculations S59–S68
10. References S69

1. General Information

1.1. General Experimental Information

Dry *i*-Pr_2NH (*N,N*-diisopropylamine, No. D0925, Sigma-Aldrich), dry degassed DMF (*N,N*-dimethylformamide, No. 044-32075, FUJIFILM Wako Pure Chemical Corporation), and dry degassed CH_2Cl_2 (No. 284505, Aldrich) were used as received. Solvents for the synthesis of substrates were dried over Molecular Sieves 4Å (Wako) before use. H_8-binap and Segphos were obtained from Takasago International Corporation. 3,3'-Diiodo-4,4'-dimethoxy-1,1'-biphenyl,[1] **4a**,[2] **4b**,[3] **9a**,[4] and **9b**[5] were prepared according to the literature. All other commercially available reagents were obtained from TCI Chemicals, FUJIFILM Wako Pure Chemical Corporation, Sigma-Aldrich, and Kanto Chemicals and used as received unless otherwise noted.

Silica gel column chromatography was performed using silica gel [Silica Gel 60 N (spherical, neutral), Kanto Chemicals] and JIS (Japanese Industrial Standards) special grade solvents. Silica gel preparative thin layer chromatography (PTLC) was performed using silica gel (Wakogel® B-5F) and JIS special grade solvents.

All reactions were carried out under an atmosphere of argon or nitrogen in oven-dried glassware with magnetic stirring.

1.2. General Analytical Information

All compounds were characterized by ^1H and ^{13}C NMR spectroscopy. Copies of the ^1H and ^{13}C NMR spectra for all new compounds can be found in the section 5 "^1H, ^{13}C, and 2D NMR Spectra of New Compounds" of the Supplementary Information. ^1H, ^{13}C, COSY, NOESY, and HMBC NMR data were collected on a Bruker AVANCE III HD 400 at ambient temperature. ^{13}C NMR data of **10** was collected on a Bruker AVANCE III HD 500 at ambient temperature. ^1H and ^{13}C NMR data of **14** were collected on a Bruker AVANCE III HD 600 at ambient temperature. All ^1H NMR experiments are reported in δ units, parts per million (ppm), and were measured relative to the signals for residual chloroform (7.26 ppm). All ^{13}C NMR spectra are reported in ppm relative to deuterochloroform (77.01 ppm) and were obtained with ^1H decoupling.

All previously unreported compounds were additionally characterized by high-resolution mass spectrometry (HRMS) using a Bruker micrOTOF Focus II instrument. Melting points were determined on a Mettler MP50 and were uncorrected. A polarimetry measurement was performed using a JASCO P-2200 circular polarimeter. Chiral HPLC analyses were performed on a JASCO HPCL 2000 series.

The UV/Vis absorption spectra in the solution state were recorded on a JASCO V-630 spectrometer with a resolution of 1.0 nm. The UV/Vis absorption spectra in the solid state were recorded on a JASCO V-560 spectrometer with a resolution of 1.0 nm. The emission spectra in the solution state were recorded on a JASCO FP-6200 spectrometer with a resolution of 1.0 nm. The emission spectrum in the solid state was recorded on a JASCO FP-8550 spectrometer with a resolution of 0.5 nm. The fluorescence quantum yields in the solution state were measured on a Hamamatsu Photonics, Absolute PL Quantum Yield Measurement System, C11347-01. The fluorescence quantum yield in the solution state was measured on a JASCO FP-8550 spectrofluorometer attached with a JASCO ILF-135 integrating sphere (diameter 120 mm).

The electronic circular dichroism (ECD) spectra were obtained on a JASCO J-820 spectrodichrometer, using a 10 mm quartz cell for solution. The magnitude of the ECD signal is expressed in terms of molar circular dichroism $\Delta\varepsilon/M^{-1}$ cm^{-1}. The circularly polarized luminescence (CPL) spectra were obtained on a JASCO CPL-300 spectrophotometer, using a 10 mm quartz cell for solution.

2. Synthesis of Cyclic Polyynes

2.1. Synthesis of 3,3'-Diiodo-[1,1'-biphenyl]-4,4'-diol (3)

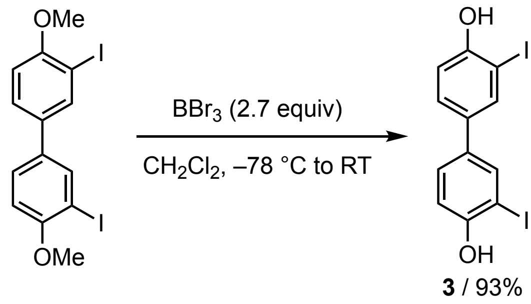

To a solution of 3,3'-diiodo-4,4'-dimethoxy-1,1'-biphenyl[1] (13.84 g, 29.7 mmol) in dry CH_2Cl_2 (300 mL) was added a solution of BBr_3 in CH_2Cl_2 (80.2 mL, 80.2 mmol) dropwise at –78 °C. The mixture was warmed to room temperature and stirred for 16 h. The resulting mixture was quenched with water, extracted with EtOAc twice, dried Na_2SO_4, and concentrated under reduced pressure. The residue was purified by silica gel column chromatography (eluent: *n*-hexane/EtOAc = 2:1) to give **3** (12.14 g, 27.7 mmol, 93% yield).

Colorless solid; mp 163.0 °C (decomposition); ^{1}H NMR (400MHz, $CDCl_3$) δ 7.79 (d, J = 2.2 Hz, 2H), 7.38 (dd, J = 2.2, 8.4 Hz, 2H), 7.03(d, J = 8.4 Hz, 2H), 5.29 (s, 2H); ^{13}C NMR (100 MHz, $CDCl_3$) δ 154.2, 136.2, 133.9, 128.6, 115.2, 86.1; HRMS (ESI) calcd for $C_{12}H_7I_2O_2$ $[M–H]^–$ 436.8541, found 436.8452.

2.2. Synthesis of 5a and 5b

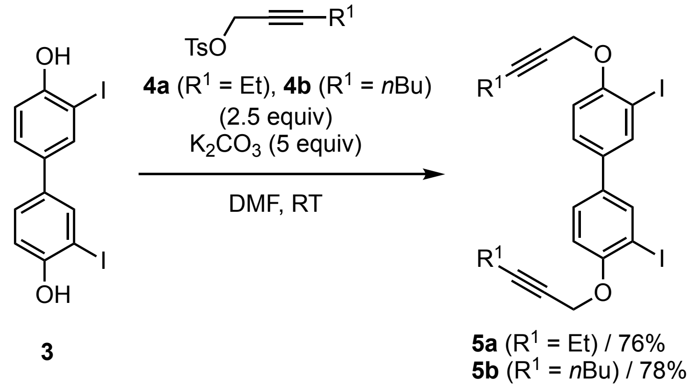

3

5a (R^1 = Et) / 76%
5b (R^1 = *n*Bu) / 78%

2.2.1. Synthesis of 3,3'-Diiodo-4,4'-bis(pent-2-yn-1-yloxy)-1,1'-biphenyl (5a)

To a solution of **3** (12.04 g, 27.5 mmol) and K_2CO_3 (18.99 g, 137.5 mmol) in dry DMF (150 mL) was added pent-2-yn-1-yl 4-methylbenzenesulfonate[2] (**4a**, 10.10 g, 68.7 mmol). The mixture was stirred at room temperature for 16 h. The resulting mixture was quenched with water, extracted with Et_2O twice, dried Na_2SO_4 and the solvent removed under reduced pressure. The residue was purified by silica gel column chromatography (eluent: *n*-hexane/EtOAc = 4:1) to give **5a** (12.00 g, 21.0 mmol, 76% yield).

Colorless solid; mp 103.5–105.5 °C; ^{1}H NMR (400MHz, $CDCl_3$) δ 7.93 (d, J = 2.2 Hz, 2H), 7.44 (dd, J = 2.2, 8.6 Hz, 2H), 7.03(d, J = 8.6 Hz, 2H), 4.76 (t, J = 2.1 Hz, 4H), 2.23 (tq, J = 2.1, 3.5 Hz, 4H), 4.76 (t, J = 3.5 Hz, 6H); ^{13}C NMR (100 MHz, $CDCl_3$) δ 156.0, 137.6, 134.4, 127.5, 113.1, 90.3, 87.1, 73.7, 57.8, 13.6, 12.5; HRMS (ESI) calcd for $C_{22}H_{20}I_2O_2Na$ $[M+Na]^+$ 592.9445, found 592.9487.

2.2.2. Synthesis of 3,3'-Diiodo-4,4'-bis(hept-2-yn-1-yloxy)-1,1'-biphenyl (5b)

To a solution of **3** (4.38 g, 10.0 mmol) and K_2CO_3 (6.91 g, 50.0 mmol) in dry DMF (100 mL) was added hept-2-yn-1-yl 4-methylbenzenesulfonate[3] (**4b**, 6.67 g, 25.0 mmol). The mixture was stirred at room temperature for 18 h. The resulting mixture was quenched with water, extracted with Et_2O twice, dried Na_2SO_4 and the solvent removed under reduced pressure. The residue was purified by washing with hexane to give **5b** (4.73g, 7.77 mmol, 78% yield).

Colorless solid; mp 69.5–70.5 °C; ^{1}H NMR (400 MHz, $CDCl_3$) δ 7.93 (d, J = 2.2 Hz, 2H), 7.44 (dd, J = 2.2, 8.5 Hz, 2H), 7.03 (d, J = 8.5 Hz, 2H), 4.77 (t, J = 2.1 Hz, 4H), 2.21 (tt, J = 2.1, 10.5 Hz, 4H), 1.33–1.52 (m, 8H) 0.89 (t, J = 7.2 Hz, 6H); ^{13}C NMR (100 MHz, $CDCl_3$) δ 156.1, 137.7, 134.4, 127.5, 113.3, 89.1, 87.2, 74.4, 57.8, 30.4, 21.9, 18.5, 13.6; HRMS (ESI) calcd for $C_{26}H_{28}I_2O_2Na$ $[M+Na]^+$ 649.0071, found 649.0090.

2.3.Synthesis of 7a and 7b

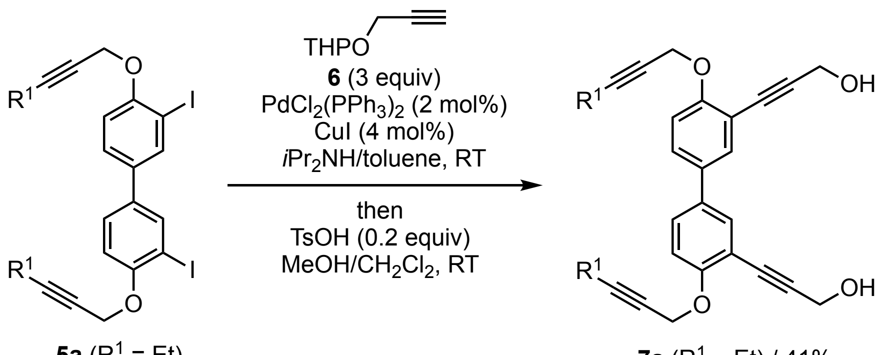

5a (R^1 = Et)
5b (R^1 = *n*Bu)

7a (R^1 = Et) / 41%
7b (R^1 = *n*Bu) / 54%

2.3.1. Synthesis of 3,3'-(4,4'-Bis(pent-2-yn-1-yloxy)-[1,1'-biphenyl]-3,3'-diyl)bis(prop-2-yn-1-ol) (7a)

To a solution of **5a** (11.40 g, 20.0 mmol), CuI (76 mg, 0.40 mmol), and $Pd(PPh_3)_2Cl_2$ (140 mg, 0.20 mmol) in *i*-Pr_2NH (70 mL) and toluene (140 ml) was added 2-(prop-2-yn-1-yloxy)tetrahydro-2H-pyran (**6**, 8.4 mL, 60.0 mmol). The mixture was stirred at room temperature for 19 h. The resulting mixture was filtered and concentrated. The residue was roughly purified by silica gel column chromatography (eluent: *n*-hexane/EtOAc = 4:1). The residue was used in the next step without further purification.

To a solution of this crude in MeOH (200 mL) and CH_2Cl_2 (50 mL) was added *p*-toluenesulfonic acid monohydrate (PTSA•H_2O, 0.761 g, 4.00 mmol). The mixture was stirred at room temperature for 2.5 h. The reaction was quenched with water and extracted with CH_2Cl_2 twice. The combined organic layer was washed with saturated aqueous NaCl, dried over Na_2SO_4, and concentrated. The residue was purified by silica gel column chromatography (eluent: *n*-hexane/EtOAc = 1:1) to give **7a** (3.49 g, 8.18 mmol, 41% yield in 2 steps).

Yellow solid; mp 127.1 °C (decomposition); ^{1}H NMR (400 MHz, $CDCl_3$) δ 7.58 (d, J = 2.3 Hz, 2H), 7.43 (dd, J = 2.4, 8.6 Hz, 2H), 7.07 (d, J = 8.7 Hz, 2H), 4.78 (t, J = 2.1 Hz, 4H), 4.55 (s, 4H), 2.21 (tq, J = 2.1, 7.5 Hz, 4H), 2.02 (s, 2H), 1.13 (t, J = 7.5 Hz, 6H); ^{13}C NMR (100 MHz, $CDCl_3$) δ 157.6, 132.8, 131.9, 127.8, 113.1, 112.7, 91.7, 90.2, 81.8, 73.9, 57.3, 51.9, 13.6, 12.5; HRMS (ESI) calcd for $C_{28}H_{26}O_4Na$ $[M+Na]^+$ 449.1723, found 449.1717.

2.3.2. Synthesis of 3,3'-(4,4'-Bis(hept-2-yn-1-yloxy)-[1,1'-biphenyl]-3,3'-diyl)bis(prop-2-yn-1-ol) (7b)

To a solution of **5b** (4.51 g, 7.4 mmol), CuI (56 mg, 0.30 mmol), and $Pd(PPh_3)_2Cl_2$ (104 mg, 0.15 mmol) in *i*-Pr_2NH (20 mL) and toluene (40 ml) was added 2-(prop-2-yn-1-yloxy) tetrahydro-2H-pyran (**6**, 3.14 mL, 22.3 mmol). The mixture was stirred at room temperature for 19 h. The resulting mixture was filtered and concentrated. The residue was roughly purified by silica gel column chromatography (eluent: n-hexane/EtOAc = 4:1) to give crude. The residue was used in the next step without further purification.

To a solution of this crude in MeOH (60 mL) and CH_2Cl_2 (40 mL) was added *p*-toluenesulfonic acid monohydrate (PTSA•H_2O, 0.282 g, 1.48 mmol). The mixture was stirred at room temperature for 2.5 h. The reaction was quenched with water and extracted with CH_2Cl_2 twice. The combined organic layer was washed with saturated aqueous NaCl, dried over Na_2SO_4, and concentrated. The residue was purified by silica gel column chromatography (eluent: *n*-hexane/EtOAc = 2:1) to give **7b** (1.84 g, 3.96 mmol, 54% yield in 2 steps).

Yellow solid; mp 100.2–101.5 °C; ^{1}H NMR (400 MHz, $CDCl_3$) δ 7.60 (d, J = 2.3 Hz, 2H), 7.44 (dd, J = 2.3, 8.7 Hz, 2H), 7.09 (d, J = 8.7 Hz, 2H), 4.79 (t, J = 2.1 Hz, 4H), 4.55 (s, 4H), 2.21 (tt, J = 2.1, 10.4 Hz, 4H), 1.82 (s, 2H), 1.33–1.51 (m, 8H), 0.89 (t, J = 7.3 Hz, 6H); ^{13}C NMR (100 MHz, $CDCl_3$) δ 157.5, 132.8, 131.8, 127.7, 113.1, 112.7, 91.8, 89.0, 81.8, 74.6, 57.3, 51.8, 30.4, 21.9, 18.5, 13.6; HRMS (ESI) calcd for $C_{32}H_{34}O_4Na$ $[M+Na]^+$ 505.2349 , found 505.2349.

2.4. Synthesis of 8a and 8b

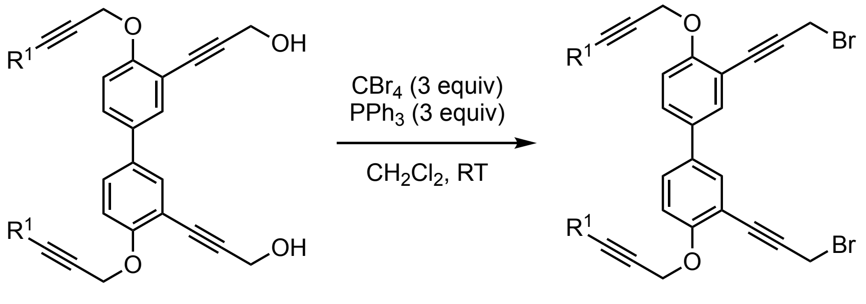

7a (R^1 = Et)
7b (R^1 = *n*Bu)

8a (R^1 = Et) / 46%
8b (R^1 = *n*Bu) / 42%

2.4.1. Synthesis of 3,3'-Bis(3-bromoprop-1-yn-1-yl)-4,4'-bis(pent-2-yn-1-yloxy)-1,1'-biphenyl (8a)

To a solution of **7a** (1.43 g, 3.35 mmol) and PPh_3 (2.65 g, 10.1 mmol) in CH_2Cl_2 (30 mL) was added a solution of CBr_4 (3.35 g, 10.1 mmol) in CH_2Cl_2 (30 mL) at 0 °C. The mixture was stirred at 0 °C for 1 h. The resulting mixture was concentrated. The residue was purified by silica gel column chromatography (eluent: *n*-hexane/EtOAc = 10:1) to give **8a** (0.843 g, 1.52 mmol, 46% yield).

Colorless solid; mp 86.3 °C (decomposition); ^{1}H NMR (400 MHz, $CDCl_3$) δ 7.60 (d, J = 2.3 Hz, 2H), 7.47 (dd, J = 2.3, 8.6 Hz, 2H), 7.09 (d, J = 8.7 Hz, 2H), 4.78 (t, J = 2.0 Hz, 4H), 4.23 (s, 4H), 2.22 (tq, J = 2.1, 7.5 Hz, 4H), 1.14 (t, J = 7.5 Hz, 6H); ^{13}C NMR (100 MHz, $CDCl_3$) δ 157.9, 132.8, 132.1, 128.3, 113.3, 112.4, 90.3, 88.4, 83.0, 73.9, 57.3, 15.7, 13.6, 12.5; HRMS (ESI) calcd for $C_{28}H_{24}Br_2O_2Na$ $[M+Na]^+$ 573.0035, found 573.0034.

2.4.2. Synthesis of 3,3'-Bis(3-bromoprop-1-yn-1-yl)-4,4'-bis(hept-2-yn-1-yloxy)-1,1'-biphenyl (8b)

To a solution of **7b** (1.80 g, 3.86 mmol) and PPh_3 (3.04 g, 11.6 mmol) in CH_2Cl_2 (50 mL) was added a solution of CBr_4 (3.85 g, 11.6 mmol) in CH_2Cl_2 (30 mL) at 0 °C. The mixture was stirred at 0 °C for 1 h. The resulting mixture was concentrated. The residue was purified by silica gel column chromatography (eluent: *n*-hexane/EtOAc = 10:1) to give **8b** (0.967 g, 1.64 mmol, 42% yield).

Colorless solid; mp 82.6–84.2 °C; ^{1}H NMR (400 MHz, $CDCl_3$) δ 7.60 (d, J = 2.4 Hz, 2H), 7.46 (dd, J = 2.4, 8.7 Hz, 2H), 7.09 (d, J = 8.7 Hz, 2H), 4.79 (t, J = 2.1 Hz, 4H), 4.23 (s, 4H), 2.21 (tt, J = 2.1, 10.5 Hz, 4H), 1.33–1.52 (m, 8H), 0.89 (t, J = 7.2 Hz, 6H); ^{13}C NMR (100 MHz, $CDCl_3$) δ 157.9, 132.8, 132.1, 128.2, 113.3, 112.4, 89.1, 88.4, 83.0, 74.5, 57.4, 30.4, 21.9, 18.5, 15.7, 13.6; HRMS (ESI) calcd for $C_{32}H_{32}Br_2O_2Na$ $[M+Na]^+$ 629.0661, found 629.0672.

2.5. Synthesis of Cyclic Polyynes 1aa, 1ab, and 1ba

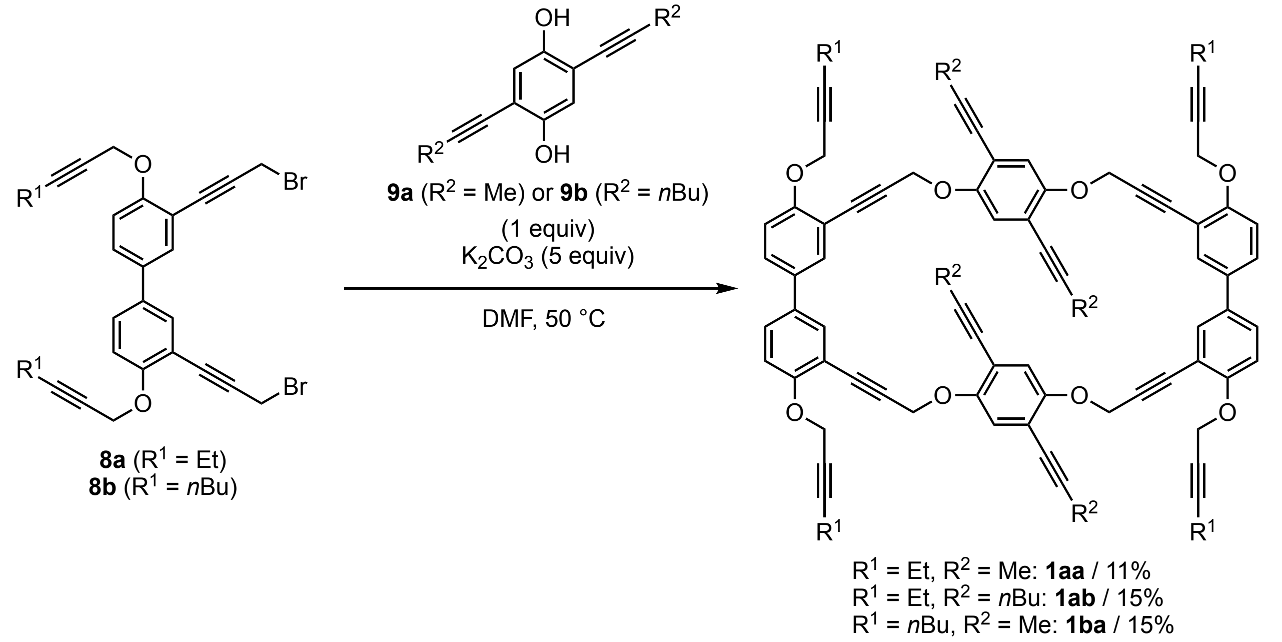

2.5.1. Synthesis of Cyclic Polyyne 1aa

To a Schlenk tube was added a solution of **8a** (0.276 g, 0.500 mmol), 2,5-di(prop-1-yn-1-yl)benzene-1,4-diol[4] (**9a**, 0.093 g, 0.500 mmol), and K_2CO_3 (0.346 g, 2.50 mmol) in DMF (25 mL, 0.02 mol/L) at room temperature. The mixture was stirred at 50 °C for 16 h. The reaction was quenched with water and combined and extracted with CH_2Cl_2 twice. The combined organic layer was washed with saturated aqueous NaCl, dried over Na_2SO_4, and concentrated. The residue was dissolved in toluene and concentrated again. The resulting mixture was purified by PTLC (eluent: *n*-hexane/CH_2Cl_2 = 1/3) to give **1aa** (32.3 mg, 0.0280 mmol, 11% yield).

Colorless Solid; mp 181.4 °C (decomposition); ^{1}H NMR (400 MHz, $CDCl_3$) δ 7.49 (d, J = 2.4 Hz, 4H), 7.39 (dd, J = 2.4, 8.6 Hz, 4H), 7.22 (s, 4H), 7.02 (d, J = 8.7 Hz, 4H), 5.01 (s, 8H), 4.76 (t, J = 2.0 Hz, 8H), 2.19 (tq, J = 2.0, 7.5 Hz, 8H), 2.09 (s, 12H), 1.11 (t, J = 7.5 Hz, 12H); ^{13}C NMR (100 MHz, $CDCl_3$) δ 157.6, 152.5, 132.7, 132.1, 127.8, 119.4, 114.6, 113.2, 112.6, 91.6, 90.1, 88.3, 84.2, 75.8, 74.1, 58.4, 57.3, 13.6, 12.5, 4.9; HRMS (ESI) calcd for $C_{80}H_{64}O_8Na$ $[M+Na]^+$ 1175.4493, found 1175.4526.

2.5.2. Synthesis of Cyclic Polyyne 1ab

To a Schlenk tube was added a solution of **8a** (0.276 g, 0.500 mmol), 2,5-di(hex-1-yn-1-yl)benzene-1,4-diol[5] (**9b**, 0.135 g, 0.500 mmol), and K_2CO_3 (0.346 g, 2.50 mmol) in DMF (25 mL, 0.02 mol/L) at room temperature. The mixture was stirred at 50 °C for 16 h. The reaction was

quenched with water and extracted with CH_2Cl_2 twice. The combined organic layer was washed with saturated aqueous NaCl, dried over Na_2SO_4, and concentrated. The residue was dissolved in toluene and concentrated again. The resulting mixture was purified by PTLC (eluent: *n*-hexane/CH_2Cl_2 = 1/3) to give **1ab** (50.4 mg, 0.0381 mmol, 15% yield).

Colorless Solid; mp 155.4 °C (decomposition); ^{1}H NMR (400 MHz, $CDCl_3$) δ 7.45 (d, J = 2.4 Hz, 4H), 7.37 (dd, J = 2.4, 8.6 Hz, 4H), 7.19 (s, 4H), 7.02 (d, J = 8.7 Hz, 4H), 5.00 (s, 8H), 4.75 (t, J = 1.9 Hz, 8H), 2.42 (t, J = 7.1 Hz, 8H), 2.19 (tq, J = 2.0, 12.5 Hz, 8H), 1.50–1.57 (m, 8 H), 1.37–1.46 (m, 8H), 1.11 (t, J = 7.5 Hz, 12H), 0.85 (t, J = 7.3 Hz, 12H); ^{13}C NMR (100 MHz, $CDCl_3$) δ 157.6, 152.7, 132.8, 132.1, 127.8, 119.8, 115.2, 114.9, 113.2, 112.6, 96.2, 90.0, 88.4, 84.1, 76.6, 74.1, 58.6, 57.3, 30.8, 22.0, 19.5, 13.6, 13.6, 12.5; HRMS (ESI) calcd for $C_{92}H_{88}O_8Na$ $[M+Na]^+$ 1343.6371, found 1343.6372.

2.5.3. Synthesis of Cyclic Polyyne 1ba

To a Schlenk tube was added a solution of **8b** (0.295 g, 0.500 mmol), 2,5-di(prop-1-yn-1-yl)benzene-1,4-diol[4] (**9a**, 0.093 g, 0.500 mmol), and K_2CO_3 (0.346 g, 2.50 mmol) in DMF (25 mL, 0.02 mol/L) at room temperature. The mixture was stirred at 50 °C for 16 h. The reaction was quenched with water and combined and extracted with CH_2Cl_2 twice. The combined organic layer was washed with saturated aqueous NaCl, dried over Na_2SO_4, and concentrated. The residue was dissolved in toluene and concentrated again. The resulting mixture was purified by PTLC (eluent: *n*-hexane/CH_2Cl_2 = 1/3) to give **1ba** (49.0 mg, 0.0387 mmol, 15% yield).

Colorless Solid; mp 112.4 °C (decomposition); ^{1}H NMR (400 MHz, $CDCl_3$) δ 7.50 (d, J = 2.4 Hz, 4H), 7.39 (dd, J = 2.4, 8.6 Hz, 4H), 7.23 (s, 4H), 7.03 (d, J = 8.7 Hz, 4H), 5.01 (s, 8H), 4.76 (t, J = 1.9 Hz, 8H), 2.19 (tt, J = 2.0, 10.4 Hz, 8H), 2.09 (s, 12H), 1.43–1.50 (m, 8H), 1.31–1.40 (m, 8H), 0.87 (t, J = 7.3 Hz, 12H); ^{13}C NMR (100 MHz, $CDCl_3$) δ 157.7, 152.6, 132.7, 132.1, 127.8, 119.4, 114.6, 113.3, 112.6, 91.6, 88.9, 88.3, 84.1, 75.8, 74.7, 58.3, 57.3, 30.4, 21.9, 18.5, 13.6, 4.9; HRMS (ESI) calcd for $C_{88}H_{80}O_8Na$ $[M+Na]^+$ 1287.5745, found 1287.5716.

3. Synthesis of Figure-Eight [10]Cyclophenylenes

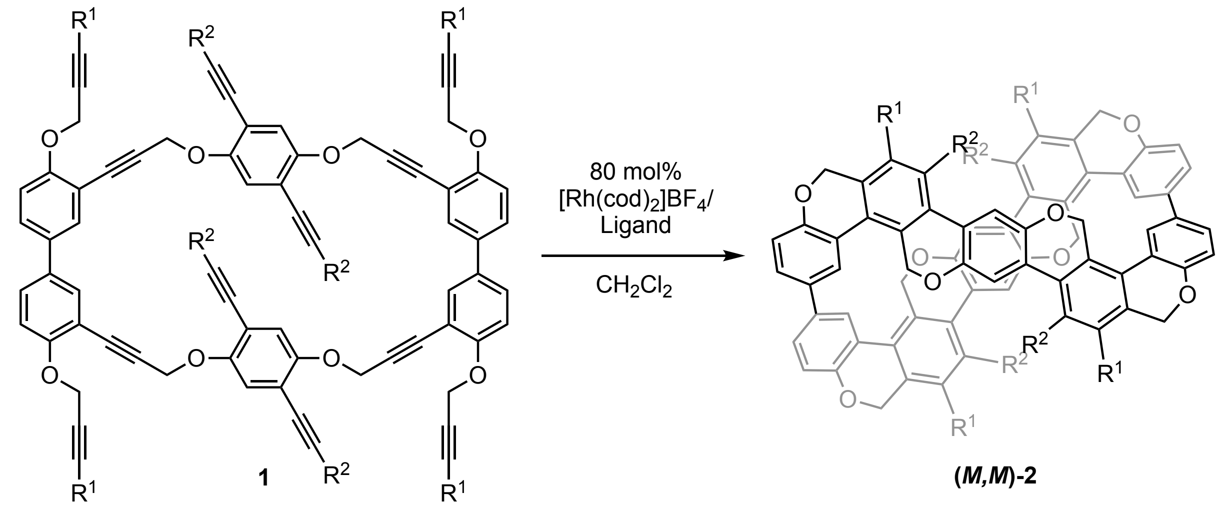

3.1.1. Synthesis of (*M,M*)-(–)-2aa (R^1 = Et, R^2 = Me)

(*S*)-tol-Segphos (9.0 mg, 0.014 mmol) and [Rh(cod)$_2$]BF_4 (5.5 mg, 0.014 mmol) were dissolved in CH_2Cl_2 (2.0 mL), and the mixture was stirred at room temperature for 30 min. H_2 was introduced to the resulting solution in a Schlenk tube. After stirring at room temperature for 30 min, the resulting mixture was concentrated to dryness. The residue was dissolved in CH_2Cl_2 (3 mL). To this solution was added a solution of **1aa** (19.5 mg, 0.0169 mmol) in CH_2Cl_2 (5.5 mL). The mixture was stirred at 40 °C for 24 h. The resulting mixture was concentrated and purified by silica gel PTLC (eluent: n-hexane/ CH_2Cl_2 = 1:4) to give (*M,M*)-(–)-**2aa** (17.0 mg, 0.0147 mmol, 87% yield, er = 89:11).

Colorless solid; mp 200.5 °C (decomposition); $[\alpha]^{25}_{D}$ –1698° (*c* 1.3 μg/cm^3, $CHCl_3$, er = 89:11); ^{1}H NMR (400 MHz, $CDCl_3$) δ 7.79 (dd, *J* = 1.9, 8.6 Hz, 4H), 7.44 (d, *J* = 1.9 Hz, 4H), 7.26 (s, 4H), 7.21 (d, *J* = 8.5 Hz, 4H), 5.49 (d, *J* = 13.2 Hz, 4H), 5.41 (d, *J* = 13.3 Hz, 4H), 5.04 (d, *J* = 13.4 Hz, 4H), 4.84 (d, *J* = 13.2 Hz, 4H), 2.69–2.84 (m, 8H), 2.60 (s, 12H), 1.22 (t, *J* = 7.5 Hz, 12H); ^{13}C NMR (100 MHz, $CDCl_3$) δ 155.7, 150.9, 138.4, 132.5, 132.2, 131.6, 131.1, 130.7, 125.8, 125.5, 125.5, 123.9, 123.4, 118.1, 117.0, 68.5, 67.0, 22.5, 18.2, 14.1; HRMS (ESI) calcd for $C_{80}H_{64}O_8Na$ $[M+Na]^+$ 1175.4493, found 1175.4418.

3.1.2. Synthesis of (*M,M*)-(–)-2ab (R^1 = Et, R^2 = *n*Bu)

(*S*)-Segphos (2.1 mg, 0.0034 mmol) and [Rh(cod)$_2$]BF_4 (1.4 mg, 0.0034 mmol) were dissolved in CH_2Cl_2 (1.0 mL), and the mixture was stirred at room temperature for 30 min. H_2 was introduced to the resulting solution in a Schlenk tube. After stirring at room temperature for 30 min, the resulting mixture was concentrated to dryness. The residue was dissolved in CH_2Cl_2 (0.5 mL). To this solution was added a solution of **1ab** (5.6 mg, 0.0042 mmol) in CH_2Cl_2 (1.6 mL). The mixture was stirred at room temperature for 16 h. The resulting mixture was concentrated and purified by silica gel PTLC (eluent: n-hexane/ CH_2Cl_2 = 1:3) to give (*M,M*)-(–)-**2ab** (3.8 mg, 0.029 mmol, 71% yield, er = 79:21).

Colorless solid; mp 161.3 °C (decomposition); $[\alpha]^{25}_{D}$ –1732° (*c* 4.2 μg/cm^3, $CHCl_3$, er = 75:25); ^{1}H NMR (400 MHz, $CDCl_3$) δ 7.84 (dd, *J* = 2.1, 8.7 Hz, 4H), 7.52 (d, *J* = 2.0 Hz, 4H), 7.32 (s, 4H), 7.20 (d, *J* = 8.6 Hz, 4H), 5.47 (d, *J* = 13.2 Hz, 4H), 5.45 (d, *J* = 12.9 Hz, 4H), 5.00 (d, *J* = 12.8 Hz, 4H), 4.85 (d, *J* = 13.2 Hz, 4H), 2.72–2.97 (m, 16H), 1.63–1.79 (m, 8H), 1.45–1.50 (m, 8H), 1.22 (t, *J* = 7.4 Hz, 12H), 0.95 (t, *J* = 7.3 Hz, 12H); ^{13}C NMR (100 MHz, $CDCl_3$) δ 155.9, 151.3, 138.3, 137.4, 133.1, 130.9, 130.8, 130.3, 126.2, 125.3, 125.2, 123.6, 123.3, 117.7, 115.9, 68.1, 67.1, 34.1, 29.9, 22.7, 21.8, 15.4, 13.8; HRMS (ESI) calcd for $C_{92}H_{88}O_8$ $[M]^+$ 1320.6474, found 1320.6489.

3.1.3. Synthesis of (*M,M*)-(–)-2ba (R^1 = *n*Bu, R^2 = Me)

(*S*)-Segphos (2.1 mg, 0.0034 mmol) and $[Rh(cod)_2]BF_4$ (1.4 mg, 0.0034 mmol) were dissolved in CH_2Cl_2 (1.0 mL), and the mixture was stirred at room temperature for 30 min. H_2 was introduced to the resulting solution in a Schlenk tube. After stirring at room temperature for 30 min, the resulting mixture was concentrated to dryness. The residue was dissolved in CH_2Cl_2 (0.5 mL). To this solution was added a solution of **1ba** (5.4 mg, 0.0043 mmol) in CH_2Cl_2 (1.6 mL). The mixture was stirred at room temperature for 16 h. The resulting mixture was concentrated and purified by silica gel PTLC (eluent: *n*-hexane/ CH_2Cl_2 = 1:3) to give (*M,M*)-(–)-**2ba** (5.1 mg, 0.040 mmol, 94% yield, er = 85:15).

Colorless solid; mp 173.5 °C (decomposition); $[\alpha]^{25}_{D}$ –1081° (*c* 0.67 mg/cm^3, $CHCl_3$, er = 75:25); ^1H NMR (400 MHz, $CDCl_3$) δ 7.79 (dd, J = 1.9, 8.7 Hz, 4H), 7.44 (d, J = 1.9 Hz, 4H), 7.26 (s, 4H), 7.21 (d, J = 8.6 Hz, 4H), 5.48 (d, J = 13.2 Hz, 4H), 5.41 (d, J = 13.4 Hz, 4H), 5.04 (d, J = 13.4 Hz, 4H), 4.83 (d, J = 13.1 Hz, 4H), 2.65-2.78 (m, 8H), 2.59 (s, 12H), 1.45–1.52 (m, 8H), 0.98 (t, J = 7.0 Hz, 12H); ^{13}C NMR (100 MHz, $CDCl_3$) δ 155.7, 150.9, 137.4, 132.7, 132.4, 131.6, 131.1, 130.7, 125.7, 125.6, 125.5, 123.9, 123.4, 118.1, 117.0, 68.5, 67.1, 32.1, 29.2, 23.1, 18.5, 13.9; HRMS (ESI) calcd for $C_{88}H_{80}O_8Na$ $[M+Na]^+$ 1287.5745, found 1287.5750.

4. Isolation of Reaction Intermediates

(*S*)-tol-Segphos (2.6 mg, 0.0040 mmol) and $[Rh(cod)_2]BF_4$ (1.6 mg, 0.0040 mmol) were dissolved in CH_2Cl_2 (2.0 mL), and the mixture was stirred at room temperature for 30 min. H_2 was introduced to the resulting solution in a Schlenk tube. After stirring at room temperature for 30 min, the resulting mixture was concentrated to dryness. The residue was dissolved in CH_2Cl_2 (2.0 mL). To this solution was added a solution of **1aa** (11.5 mg, 0.010 mmol) in CH_2Cl_2 (3.0 mL). The mixture was stirred at an indicated temperature for 16 h. The resulting mixture was concentrated and purified by silica gel PTLC twice (eluent: *n*-hexane/ CH_2Cl_2 = 1:2 and *n*-hexane/ EtOAc = 4:1) to give intermediates **10**–**14**, as well as **2aa**.

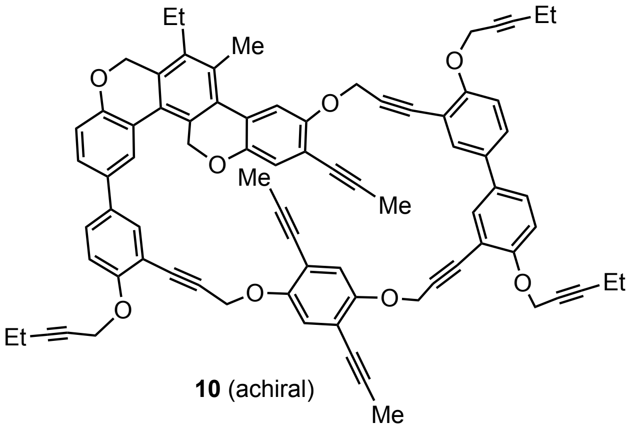

10 (achiral)

The structure of **10** was determined from the following NMR spectral analysis: (1) Eighteen C peaks in alkyne region (91.7–74.01 ppm), (2) five singlet methylene H peaks (5.23–4.90 ppm), and (3) one Me peak on the benzene ring (2.68 ppm) and three alkyne Me peaks (2.13, 2.04, and 1.89 ppm).

Data for **10**: Colorless Solid; ^{1}H NMR (400 MHz, $CDCl_3$) δ 7.60 (s, 1H), 7.48-7.51 (m, 2H), 7.43 (dd, *J* = 2.0, 8.4 Hz, 1H), 7.39-7.42 (m, 2H), 7.33 (dd, *J* = 2.5, 8.6 Hz, 1H), 7.09-7.21 (m, 4H), 7.18 (s, 1H), 7.11 (s, 1H), 7.00 (d, *J* = 8.6 Hz, 1H), 6.99 (s, 1H), 6.89 (d, *J* = 8.6 Hz, 1H), 5.23 (s, 2H), 5.15 (s, 2H), 5.10 (s, 2H), 4.97 (s, 2H), 4.90 (s, 2H), 4.79 (t, *J* = 2.0 Hz, 2H), 4.62 (t, *J* = 2.0 Hz, 2H), 4.53 (t, *J* = 2.0 Hz, 2H), 2.78 (q, *J* = 7.3 Hz, 2H), 2.68 (s, 3H), 2.08–2.26 (m, 6H), 2.13 (s, 3H), 2.04 (s, 3H), 1.89 (s, 3H), 1.23 (t, *J* = 8.0 Hz, 3H), 1.13 (t, *J* = 7.5 Hz, 3H), 1.11 (t, *J* = 7.5 Hz, 3H), 1.06 (t, *J* = 7.5 Hz, 3H).; ^{13}C NMR (125 MHz, $CDCl_3$) *δ* 157.8, 157.71, 157.69, 155.7, 152.8, 152.6, 152.1, 151.0, 138.2, 133.78, 133.76, 133.3, 133.0, 132.6, 132.5, 132.20, 132.16, 132.0, 131.1, 130.9, 128.2, 128.1, 128.0, 127.2, 126.6, 125.9, 125.6, 123.7, 121.4, 119.3, 118.4, 117.6, 115.1, 114.4, 114.3, 114.1, 113.4, 113.0, 112.8, 112.6, 112.5, 91.7, 91.6, 90.9, 90.2, 90.12, 90.06, 88.5, 88.13, 88.10, 84.3, 84.0, 83.4, 75.74, 75.71, 75.68, 74.1, 74.02, 74.01, 68.4, 67.1, 58.6, 58.5, 57.9, 57.4, 57.13, 57.06, 43.4, 29.7, 22.6, 18.3, 14.2, 13.6, 13.5, 12.54, 12.51, 12.48, 4.9, 4.8, 4.6; HRMS (ESI) calcd for $C_{80}H_{64}O_8Na$ $[M+Na]^+$ 1175.4493, found 1175.4435.

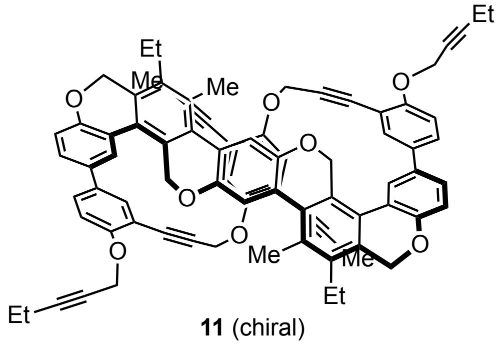

11 (chiral)

The structure of **11** was determined from the following NMR spectral analysis: (1) Six C peaks in alkyne region (91.8–74.0 ppm), (2) six doublet methylene H peaks in the cyclic structure (5.47, 5.38, 5.11, 4.92, 4.87, and 4.82 ppm), (3) one singlet methylene H peaks in the side chain (4.80 ppm, 4H), (4) one Me peak on the benzene ring (2.60 ppm, 6H). Additionally, a sufficient quantity of this compound was isolated so that the structure was also determined by 2D NMR (Figure S7~S9).

Data for (–)-**11**: Colorless Solid; $[\alpha]^{25}_{D}$ –307° (*c* 9.2 μg /cm3, $CHCl_3$, er = 96:4); ^1H NMR (400 MHz, $CDCl_3$) δ 7.54-7.58 (m, 4H), 7.45 (dd, *J* = 2.1, 8.4 Hz, 2H), 7.32 (s, 2H), 7.21 (d, *J* = 2.0 Hz, 2H), 7.17 (d, *J* = 8.4 Hz, 2H), 7.15 (d, *J* = 9.4 Hz, 2H), 7.08 (s, 2H), 5.47 (d, *J* = 13.3 Hz, 2H), 5.38 (d, *J* = 13.2 Hz, 2H), 5.11 (d, *J* = 13.3 Hz, 2H), 4.92 (d, *J* = 15.7 Hz, 2H), 4.87 (d, *J* = 15.7 Hz, 2H), 4.82 (d, *J* = 10.7 Hz, 2H), 4.80 (s, 4H), 2.68–2.87 (m, 4H), 2.60 (s, 6H), 2.23 (tq, *J* = 2.0, 12.5 Hz, 4H), 1.91 (s, 6H), 1.23 (t, *J* = 7.5 Hz, 6H), 1.15 (t, *J* = 7.5 Hz, 6H).; ^{13}C NMR (100 MHz, $CDCl_3$) *δ* 157.7, 155.9, 153.3, 151.2, 138.1, 133.9, 133.7, 132.6, 132.4, 132.0, 131.0, 130.8, 128.2, 127.3, 126.5, 125.8, 125.3, 123.9, 118.3, 117.4, 116.8, 114.2, 113.4, 112.8, 91.8, 90.2, 88.4, 83.4, 75.5, 74.0, 68.4, 67.2, 58.9, 57.4, 22.5, 17.8, 14.1, 13.6, 12.6, 4.7; HRMS (ESI) calcd for $C_{80}H_{64}O_8Na$ $[M+Na]^+$ 1175.4493, found 1175.4512.

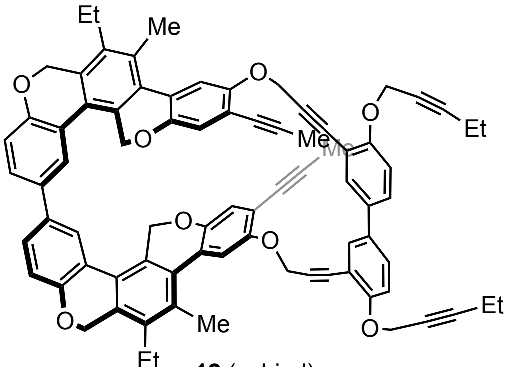

12 (achiral)

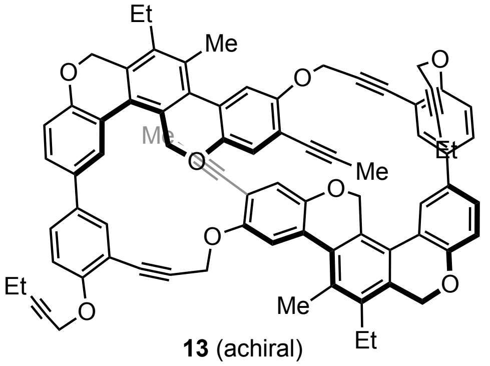

13 (achiral)

These structures are intermediate with double [2+2+2] cycloaddition reactions proceeded (**12** and **13**) because of the two Me peaks on the benzene ring [2.63 (s, 6H: mol-A), 2.49 (s, 6H: mol-B), 2.16 (s, 6H: mol-B), 2.12 (s, 6H: mol-A)] from ^1H NMR spectral analysis. The two C_2 symmetric structures that are not **11** are **12** and **13**.

Data for a mixture of **12** and **13** (mol-A/mol-B = 1:1.2, undetermined whether mol-A or mol-B is **12** or **13**): Colorless Solid ; ^1H NMR (400 MHz, $CDCl_3$) δ. 7.66 (s, 2H: mol-A), 7.38-7.46 (m, 6H: mol-B), 7.37 (d, *J* = 2.4 Hz, 2H: mol-A), 7.29-7.34 (m, 4H: mol-B), 7.23 (d, *J* = 1.9 Hz, 2H: mol-A), 7.17 (d, *J* = 8.2 Hz, 2H: mol-A), 7.09 (d, *J* = 8.7 Hz, 2H: mol-B), 7.07 (s, 2H: mol-B), 7.05 (d, *J* = 8.3 Hz, 2H: mol-B), 6.99 (d, *J* = 8.7 Hz, 2H: mol-A), 6.98 (s, 2H: mol-A), 6.89–6.93 (m, 4H: mol-A), 4.79 (s, 6H), 4.69 (t, *J* = 2.0 Hz, 4H), 2.63 (s, 6H: mol-A), 2.49 (s, 6H: mol-B), 2.16 (s, 6H: mol-B), 2.12 (s, 6H: mol-A), 1.24 (t, *J* = 7.8 Hz, 6H), 1.15 (t, *J* = 7.5 Hz, 6H), 1.10 (t, *J* = 7.5 Hz, 6H), 1.02 (t, *J* = 6.9 Hz, 6H).

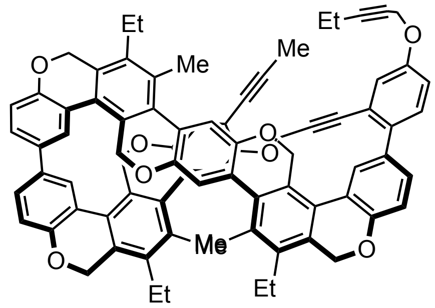

14 (chiral)

The structure of **14** was determined from the following NMR spectral analysis: (1) Six C peaks in alkyne region (91.3–74.0 ppm), (2) three Me peak on the benzene ring (2.60, 2.58, and 2.54 ppm) and one alkyne Me peaks (2.08 ppm).

Data for (–)-**14**: Colorless Solid; $[\alpha]^{25}_{D}$ –235° (*c* 3.6 μg /cm3, $CHCl_3$, er = 80:20); ^{1}H NMR (600 MHz, $CDCl_3$)δ 7.65 (d, *J* = 2.3 Hz, 1H), 7.63 (dd, *J* = 2.4, 8.8 Hz, 1H), 7.55-7.60 (m, 4H), 7.35 (s, 1H), 7.27 (s, 1H), 7.22 (s, 1H), 7.21 (d, *J* = 8.5 Hz, 1H), 7.18 (d, *J* = 8.5 Hz, 2H), 7.15 (d, *J* = 8.8 Hz, 1H), 7.13 (d, *J* = 1.9 Hz, 1H), 7.05 (d, *J* = 2.0 Hz, 1H), 6.89 (s, 1H), 5.49 (d, *J* = 13.5 Hz, 1H), 5.46 (d, *J* = 13.5 Hz, 1H), 5.46 (d, *J* = 13.2 Hz, 1H), 5.35 (d, *J* = 13.4 Hz, 1H), 5.33 (d, *J* = 12.8 Hz, 1H), 5.32 (d, *J* = 15.1 Hz, 1H), 5.17 (d, *J* = 13.0 Hz, 1H), 5.15 (d, *J* = 13.0 Hz, 1H), 4.97 (d, *J* = 13.3 Hz, 1H), 4.94 (d, *J* = 13.3 Hz, 1H), 4.92 (d, *J* = 13.3 Hz, 1H), 4.89 (d, *J* = 13.4 Hz, 1H), 4.84 (s, 2H), 4.82 (d, *J* = 15.4 Hz, 1H), 4.80 (d, *J* = 13.4 Hz, 1H), 2.66-2.84 (m, 6H), 2.60 (s, 3H), 2.58 (s, 3H), 2.54 (s, 3H), 2.24 (tq, *J* = 2.0, 12.5 Hz, 2H), 2.08 (s, 3H), 1.23 (t, *J* = 7.5 Hz, 3H), 1.22 (t, *J* = 7.4 Hz, 3H), 1.17 (t, *J* = 7.5 Hz, 3H), 1.15 (t, *J* = 7.5 Hz, 3H).; ^{13}C NMR (150 MHz, $CDCl_3$) *δ* 157.9, 155.9, 155.6, 155.3, 154.8, 151.9, 151.2, 151.1, 138.23, 138.19, 134.1, 132.9, 132.7, 132.6, 132.53, 132.46, 132.3, 131.9, 131.3, 130.9, 130.8, 130.70, 130.67, 127.8, 126.6, 126.5, 126.4, 126.2, 125.83, 125.81, 125.43, 125.39, 125.1, 124.2, 123.9, 123.8, 121.4, 118.8, 117.8, 117.6, 116.7, 116.5, 115.3, 113.4, 112.9, 91.3, 90.2, 89.0, 83.3, 76.0, 74.0, 69.0, 68.5, 68.1, 67.3, 67.2, 67.1, 62.0, 57.2, 31.9, 29.7, 22.5, 22.4, 22.3, 17.9, 17.8, 17.7, 14.06, 14.02, 13.99, 13.6, 12.5, 4.8; HRMS (ESI) calcd for $C_{80}H_{64}O_8Na$ $[M+Na]^+$ 1175.4493, found 1175.4504.

5. ^{1}H, ^{13}C, and 2D NMR Spectra of New Compounds

3,3'-Diiodo-[1,1'-biphenyl]-4,4'-diol (3)

^{1}H NMR ($CDCl_3$, 400 MHz)

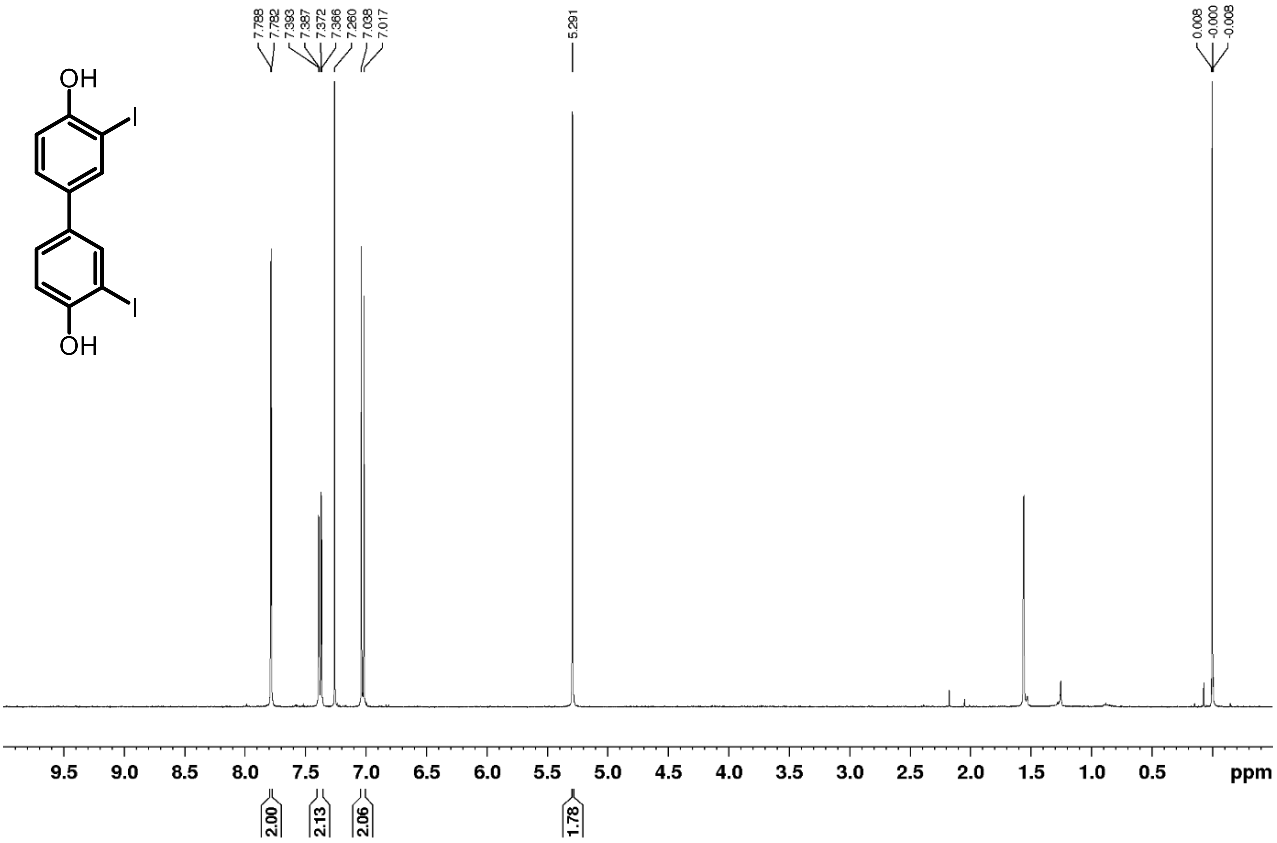

^{13}C NMR ($CDCl_3$, 100 MHz)

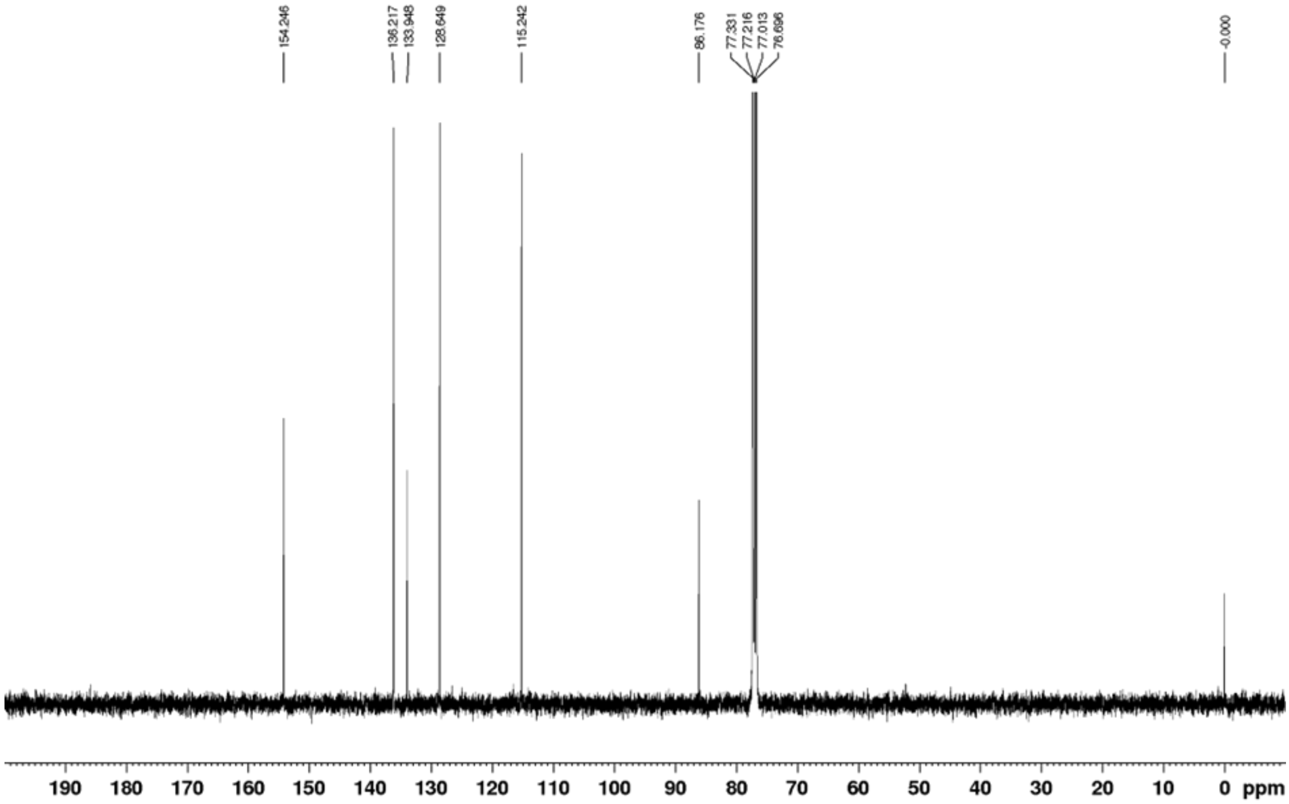

3,3'-Diiodo-4,4'-bis(pent-2-yn-1-yloxy)-1,1'-biphenyl (5a)

^{1}H NMR ($CDCl_3$, 400 MHz)

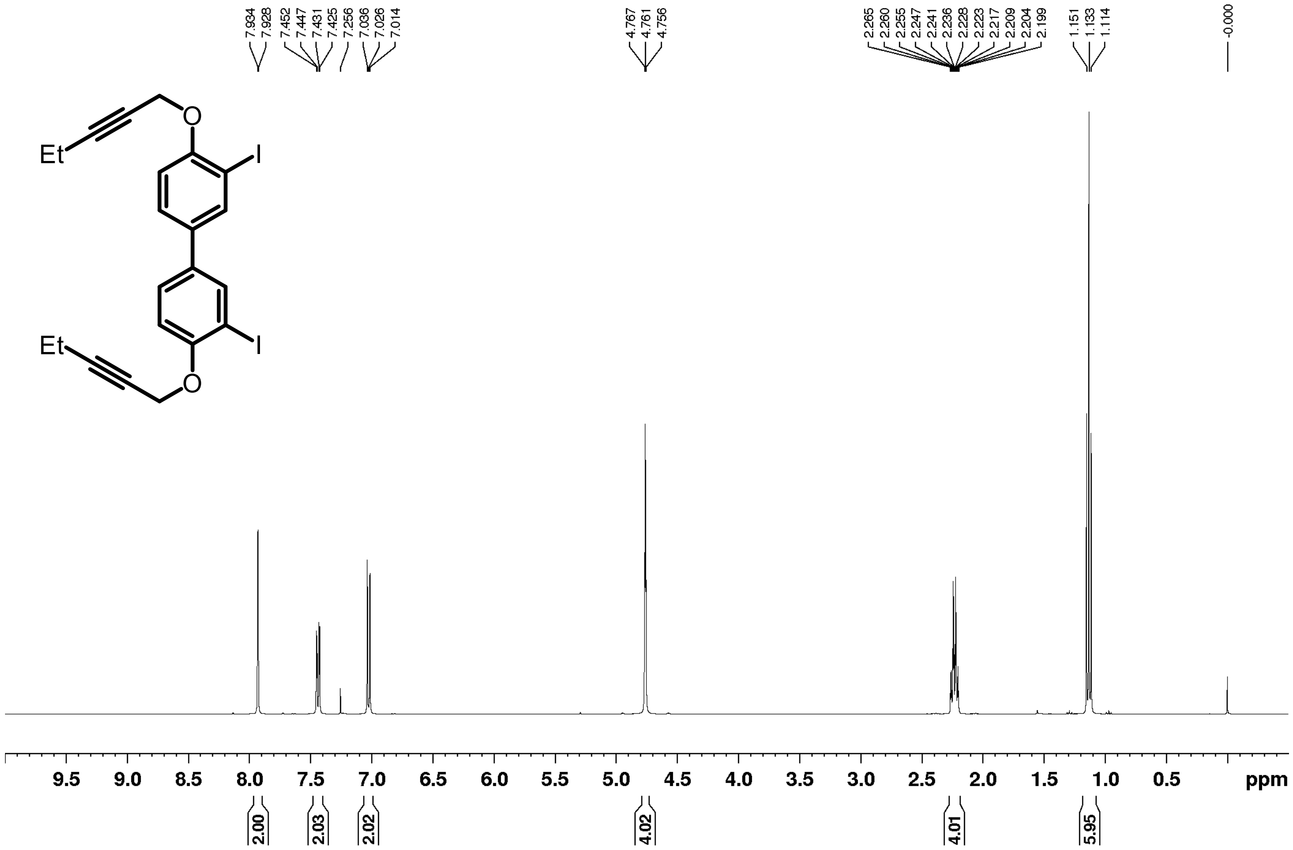

^{13}C NMR ($CDCl_3$, 100 MHz)

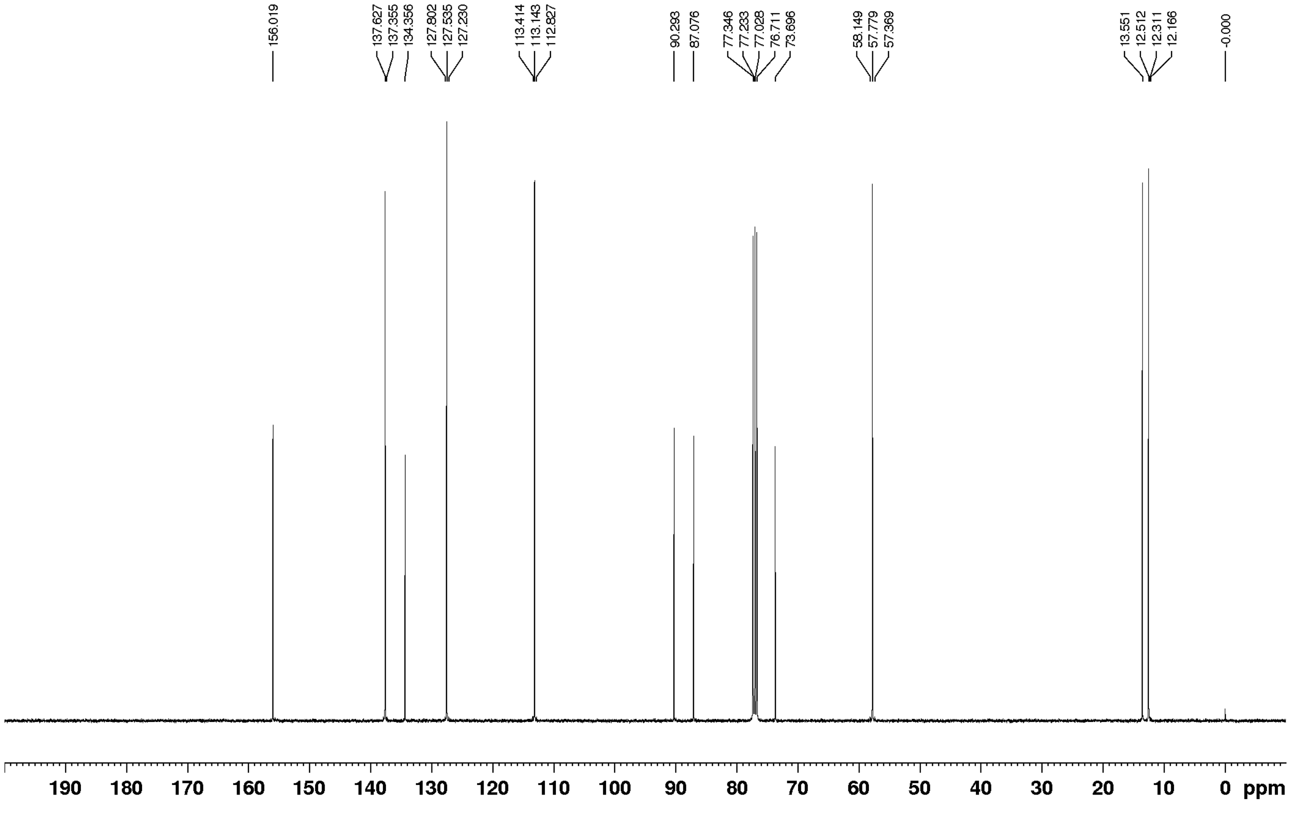

3,3'-Diiodo-4,4'-bis(pent-2-yn-1-yloxy)-1,1'-biphenyl (5b)

^{1}H NMR ($CDCl_3$, 400 MHz)

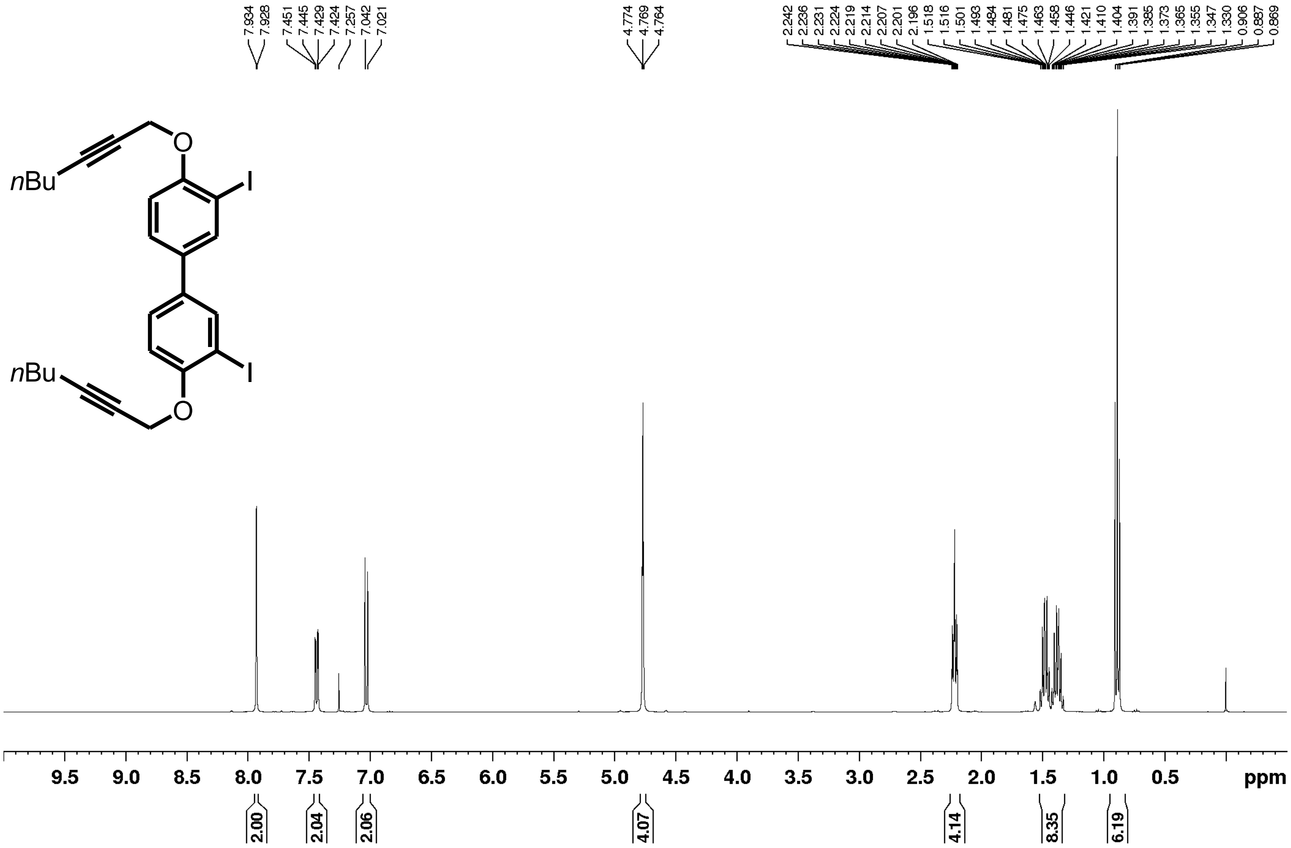

^{13}C NMR ($CDCl_3$, 100 MHz)

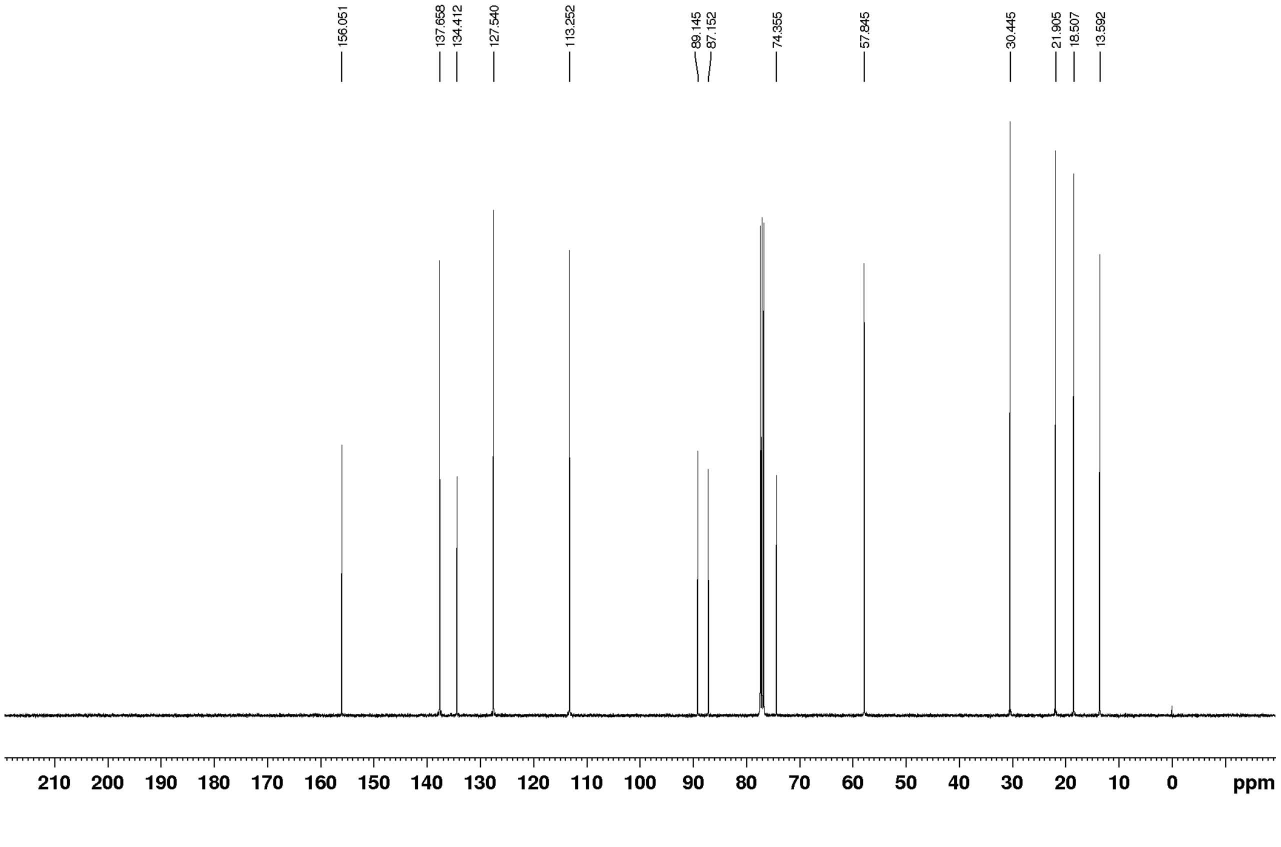

3,3'-(4,4'-Bis(pent-2-yn-1-yloxy)-[1,1'-biphenyl]-3,3'-diyl)bis(prop-2-yn-1-ol) (7a)

^{1}H NMR ($CDCl_3$, 400 MHz)

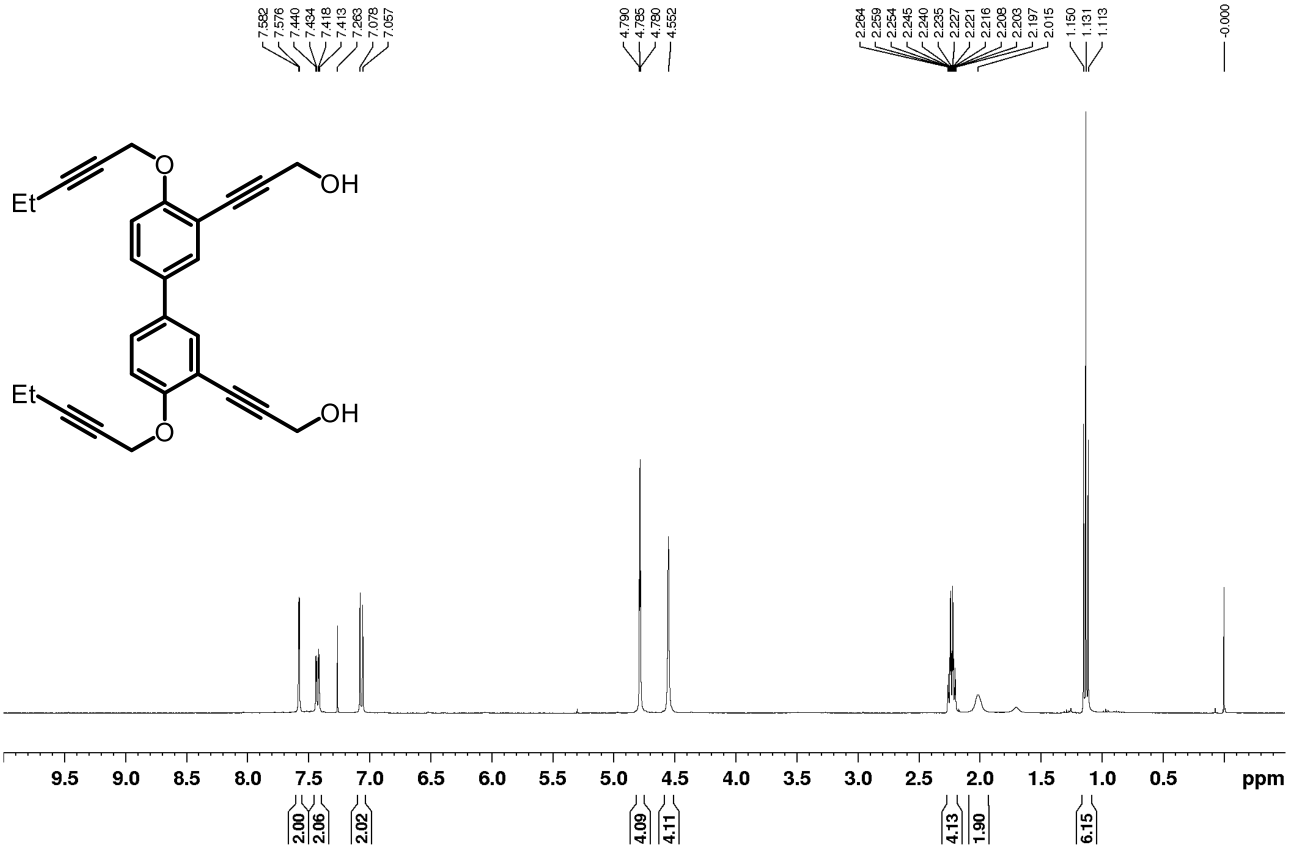

^{13}C NMR ($CDCl_3$, 100 MHz)

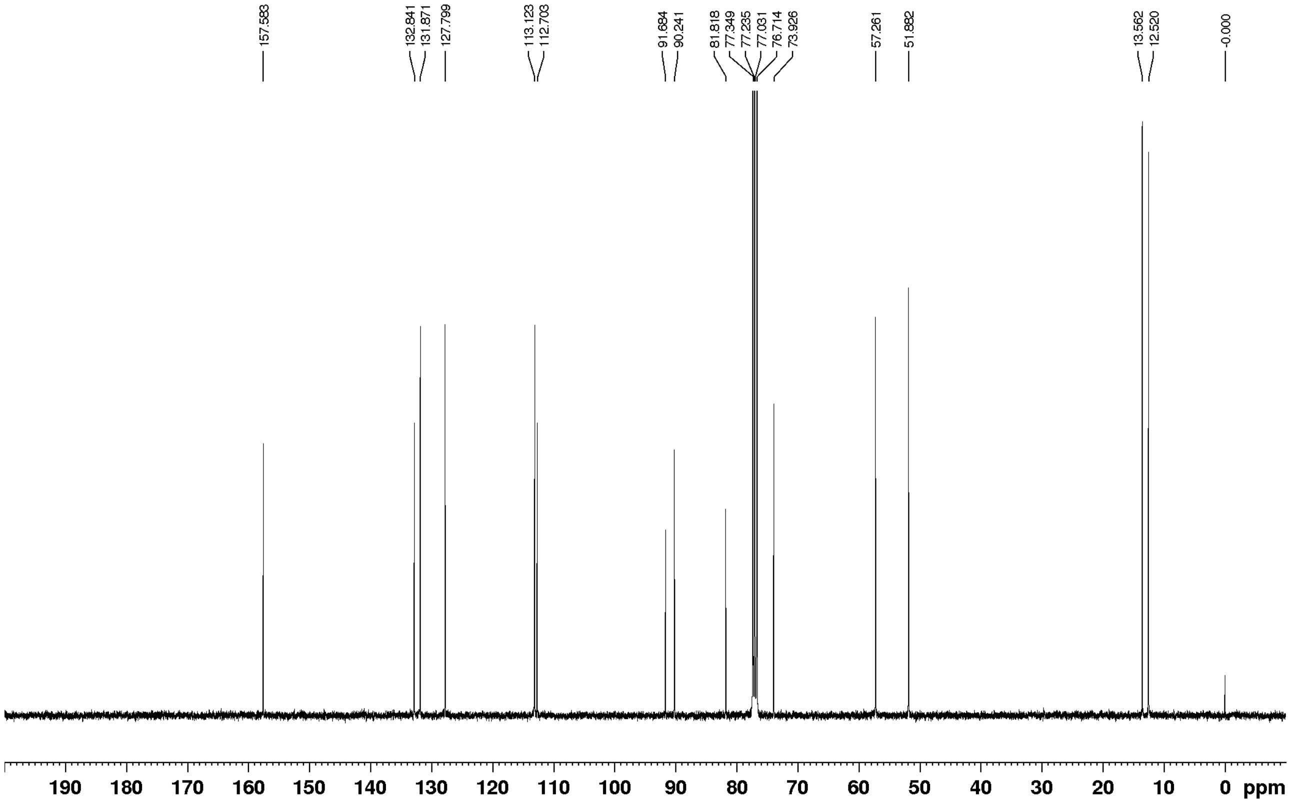

3,3'-(4,4'-Bis(hept-2-yn-1-yloxy)-[1,1'-biphenyl]-3,3'-diyl)bis(prop-2-yn-1-ol) (7b)

^{1}H NMR ($CDCl_3$, 400 MHz)

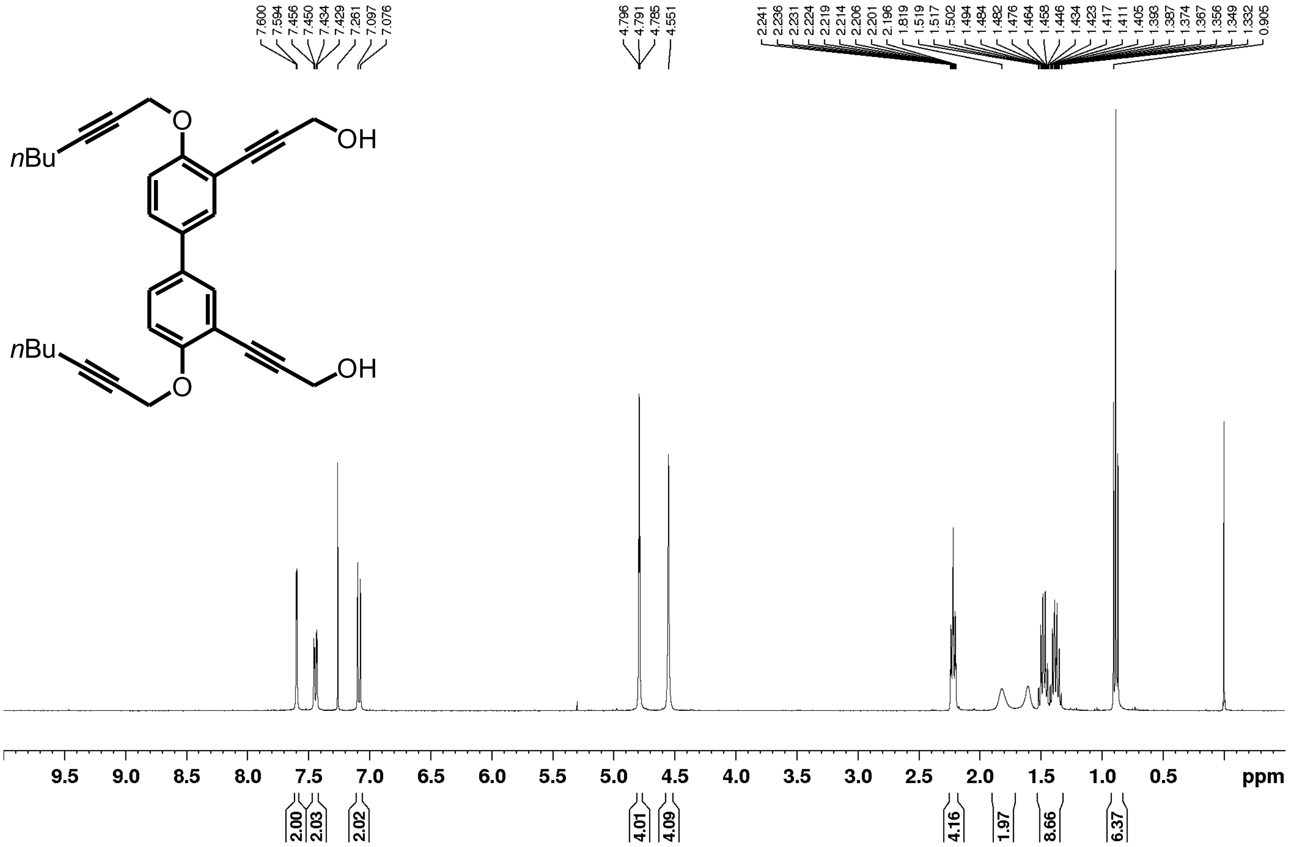

^{13}C NMR ($CDCl_3$, 100 MHz)

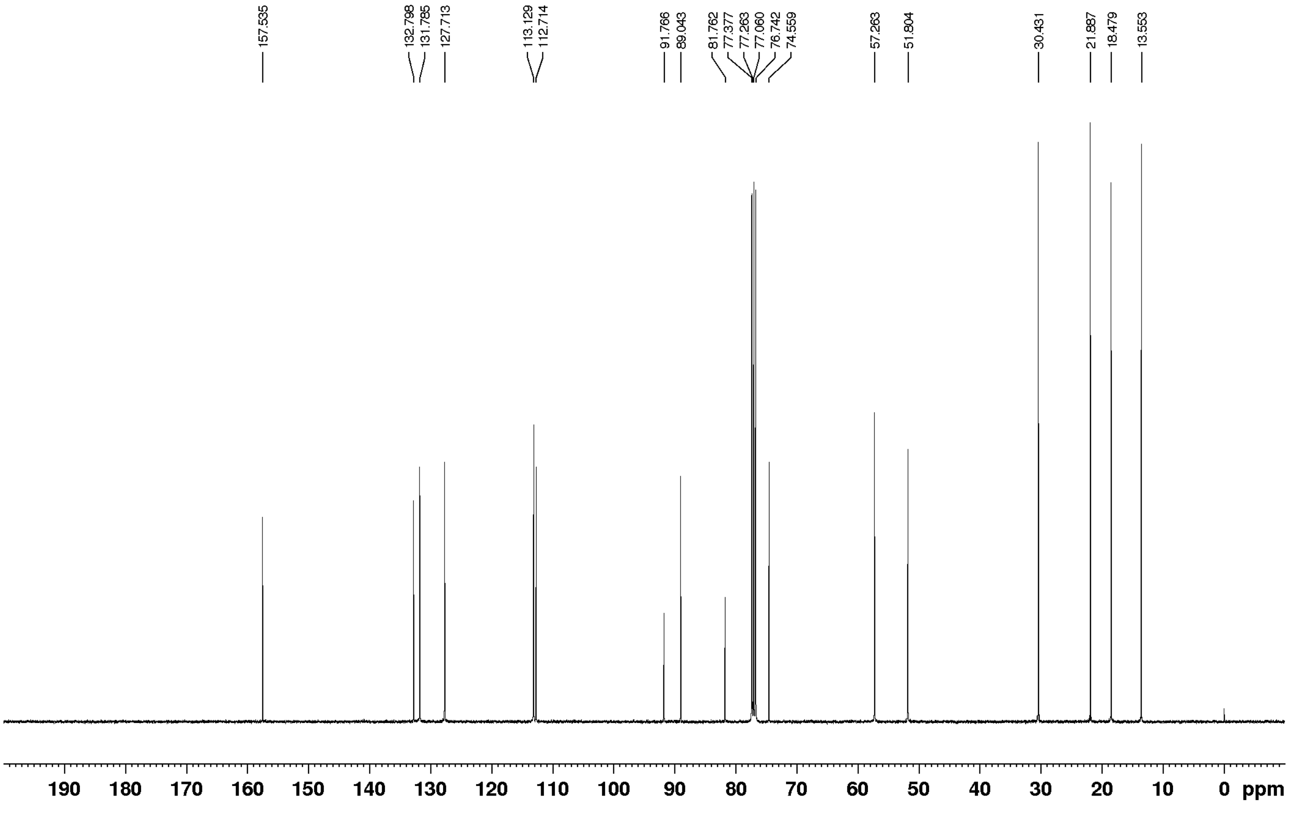

3,3'-Bis(3-bromoprop-1-yn-1-yl)-4,4'-bis(pent-2-yn-1-yloxy)-1,1'-biphenyl (8a)

^{1}H NMR ($CDCl_3$, 400 MHz)

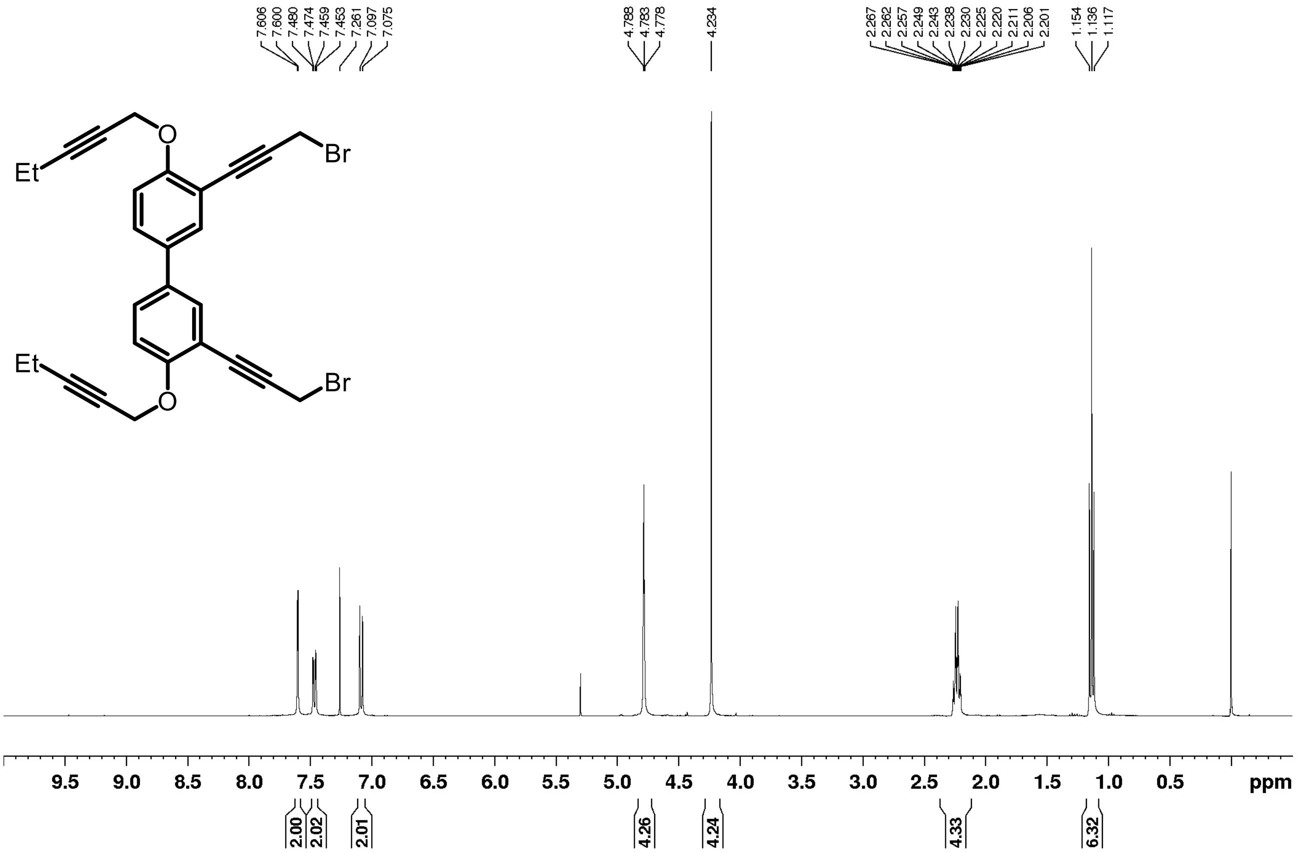

^{13}C NMR ($CDCl_3$, 100 MHz)

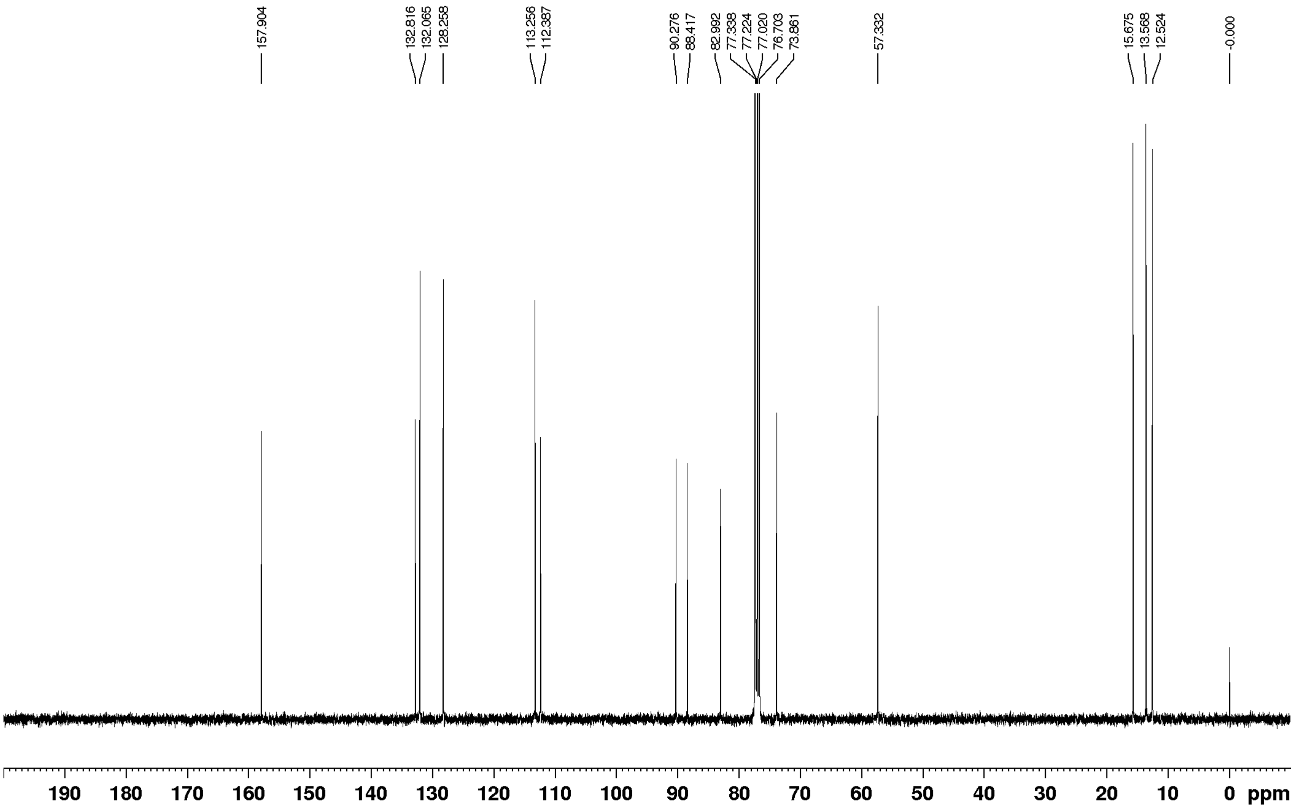

3,3'-Bis(3-bromoprop-1-yn-1-yl)-4,4'-bis(hept-2-yn-1-yloxy)-1,1'-biphenyl (8b)

^{1}H NMR ($CDCl_3$, 400 MHz)

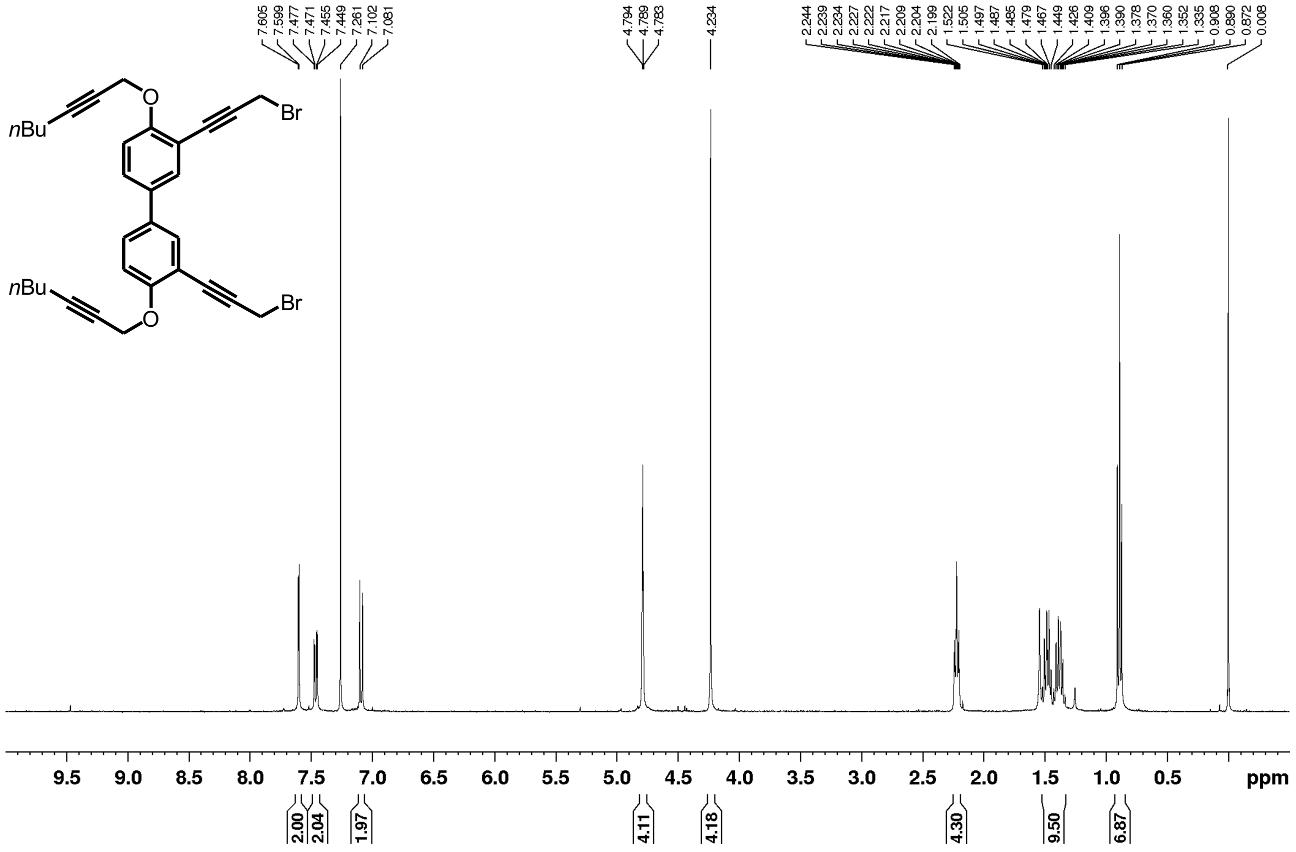

^{13}C NMR ($CDCl_3$, 100 MHz)

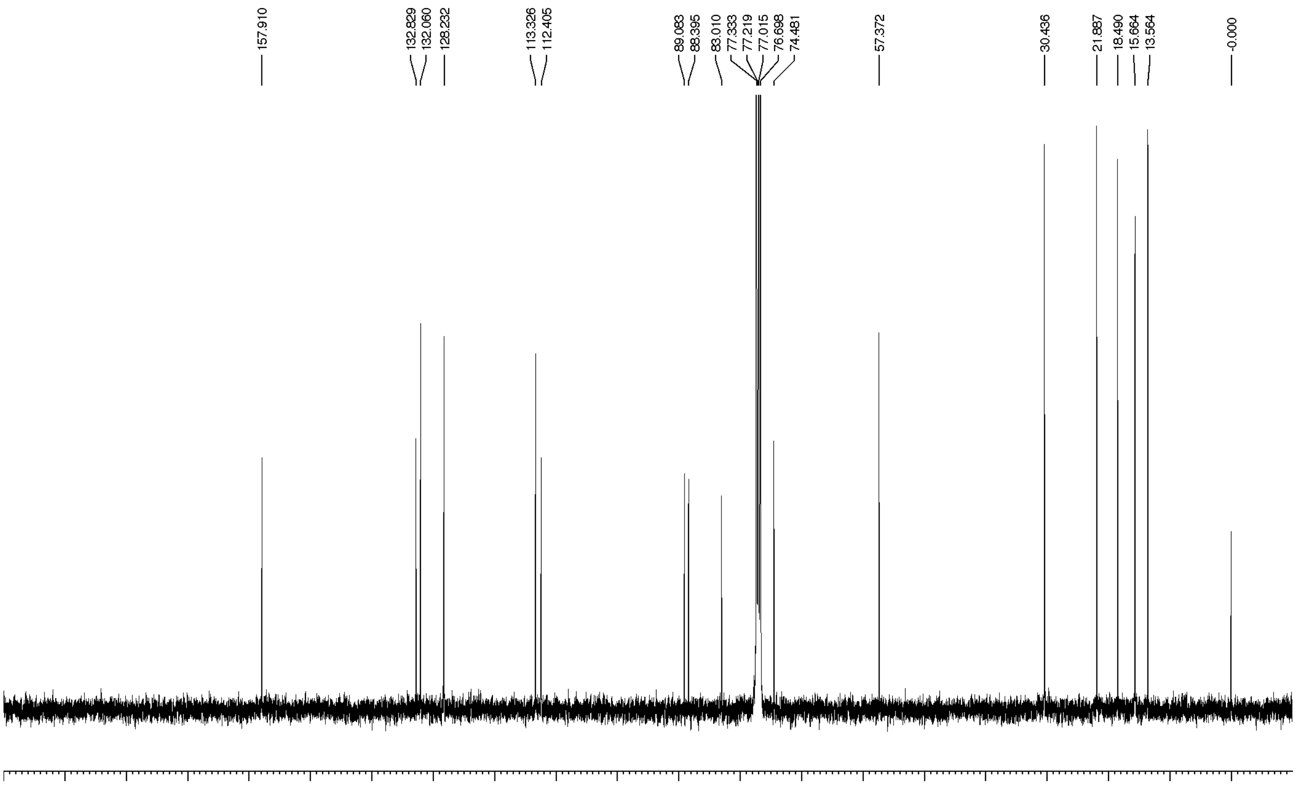

Macrocycle 1aa

^{1}H NMR ($CDCl_3$, 400 MHz)

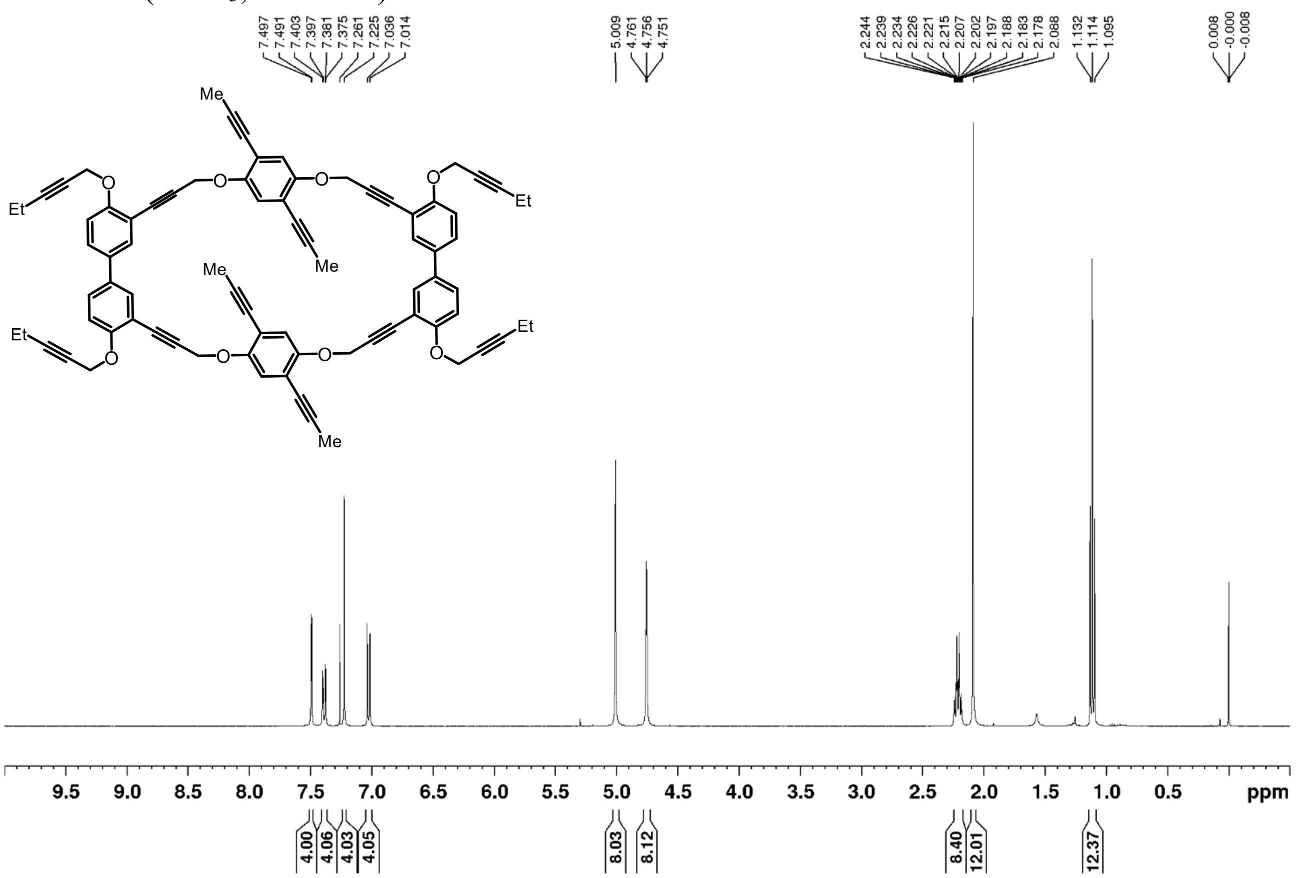

^{13}C NMR ($CDCl_3$, 100 MHz)

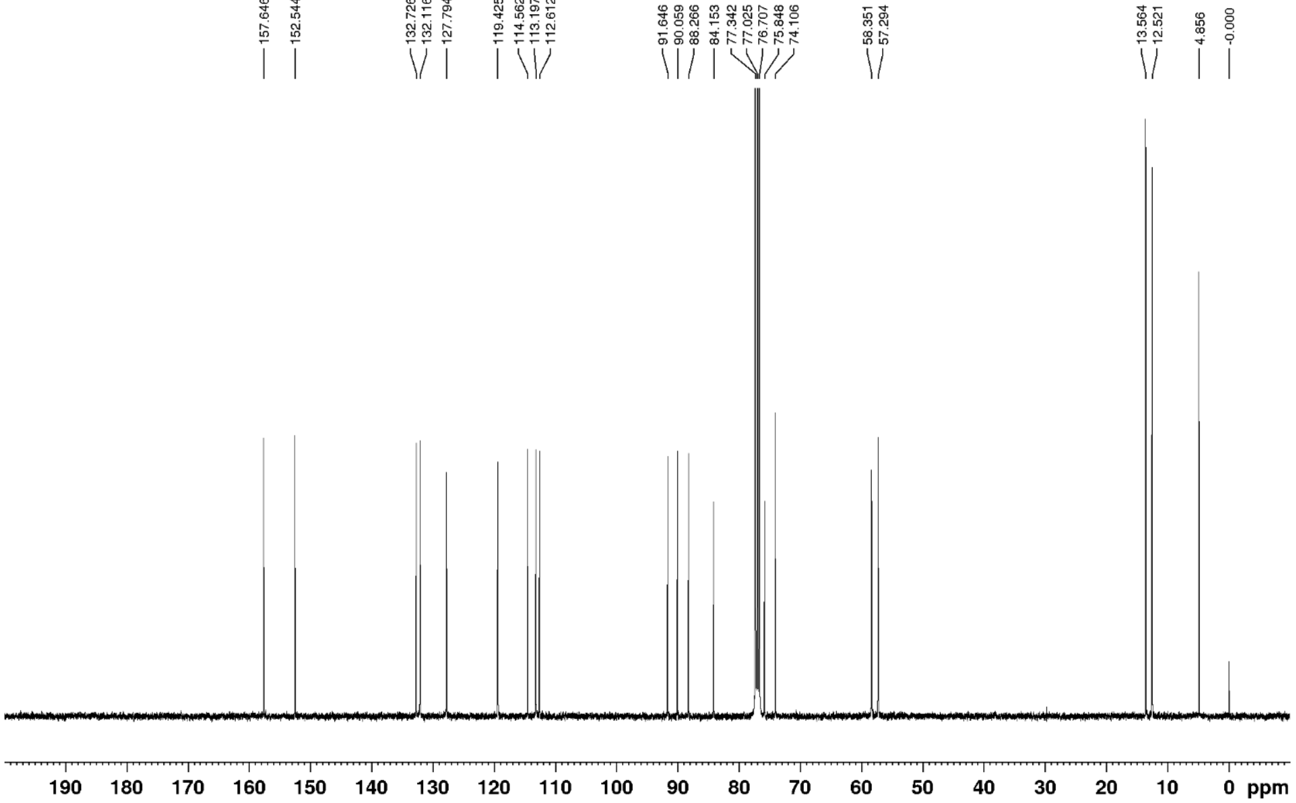

Macrocycle 1ab

^{1}H NMR ($CDCl_3$, 400 MHz)

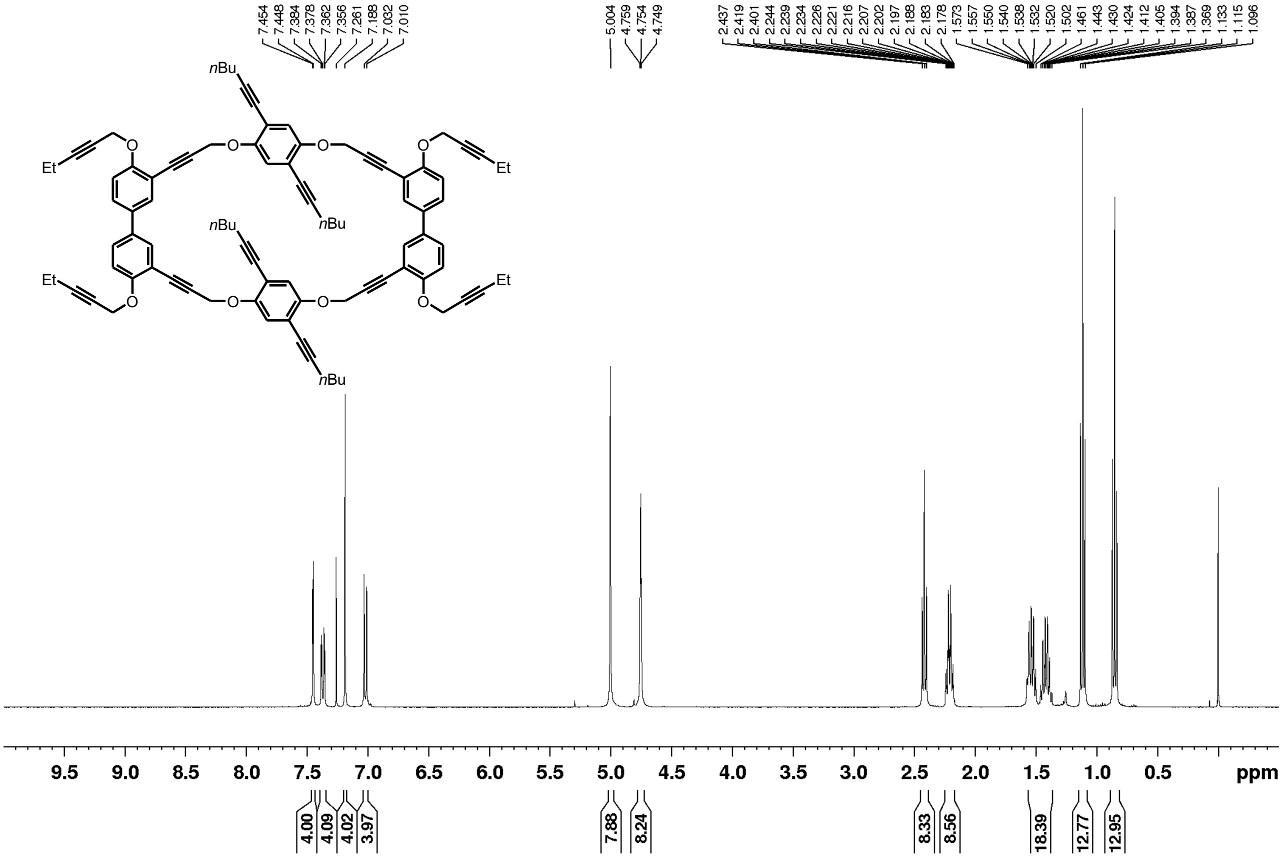

^{13}C NMR ($CDCl_3$, 100 MHz)

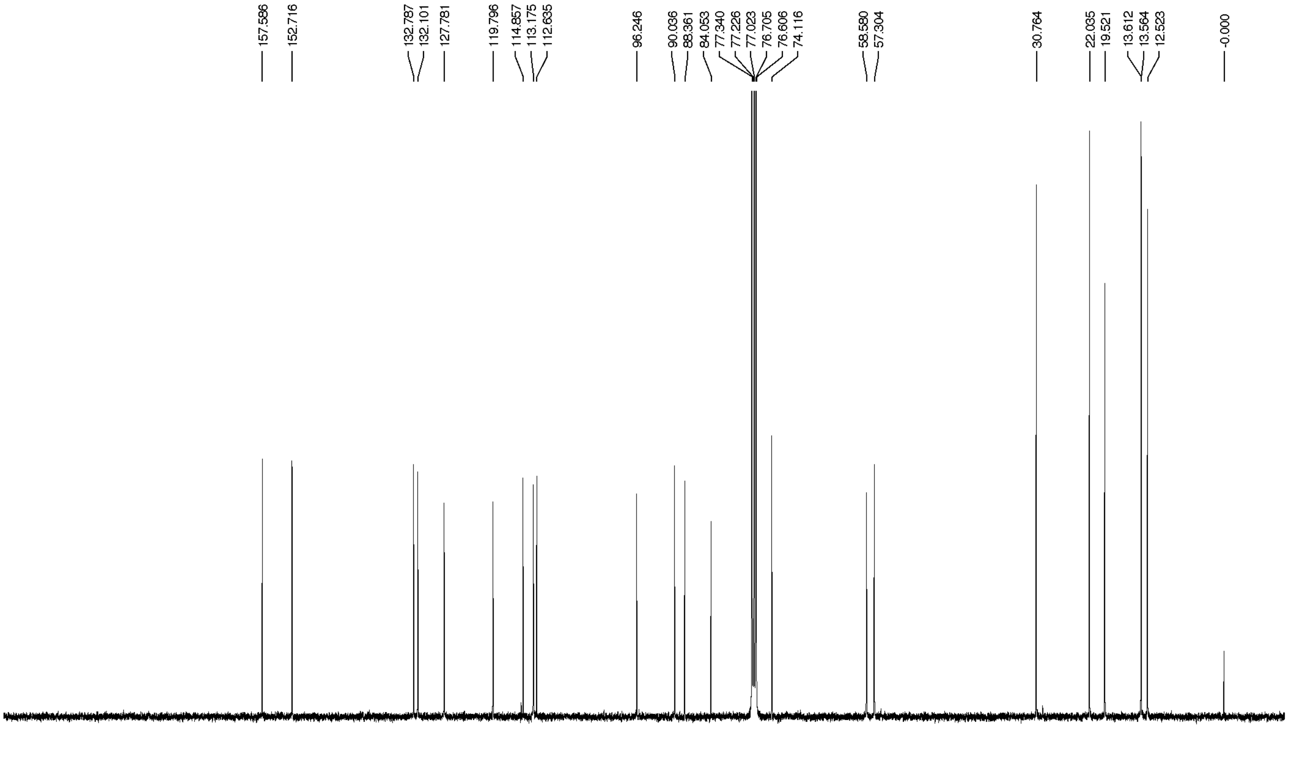

Macrocycle 1ba

^{1}H NMR ($CDCl_3$, 400 MHz)

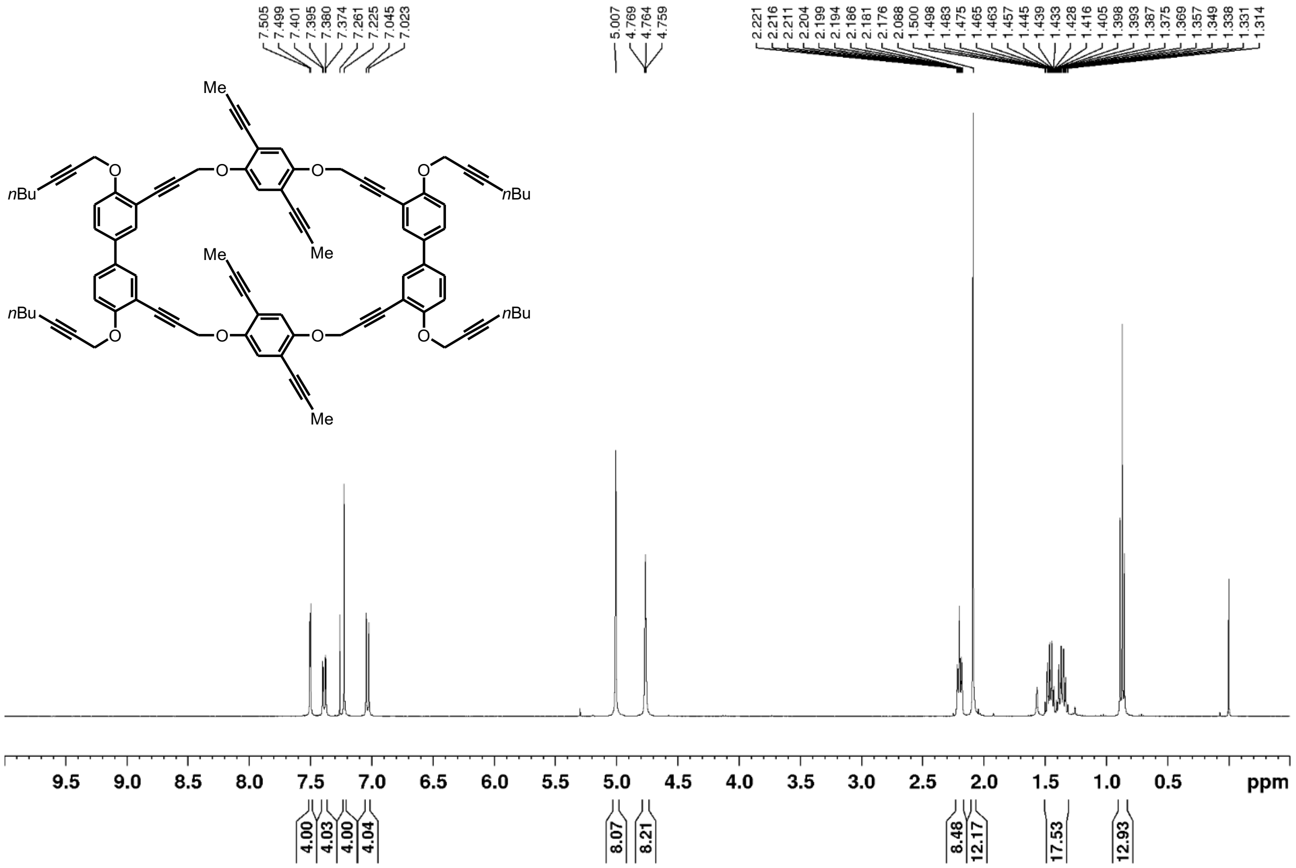

^{13}C NMR ($CDCl_3$, 100 MHz)

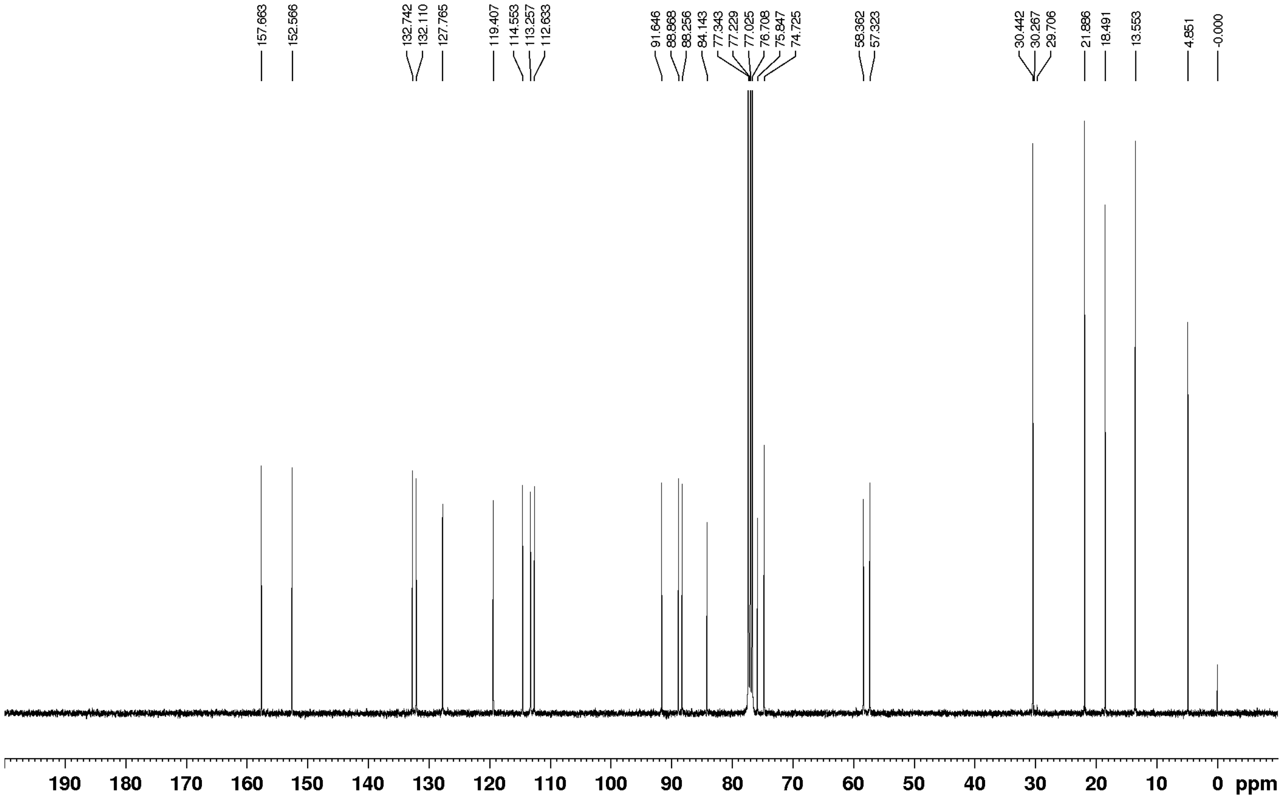

Figure-eight molecule 2aa

^{1}H NMR ($CDCl_3$, 400 MHz)

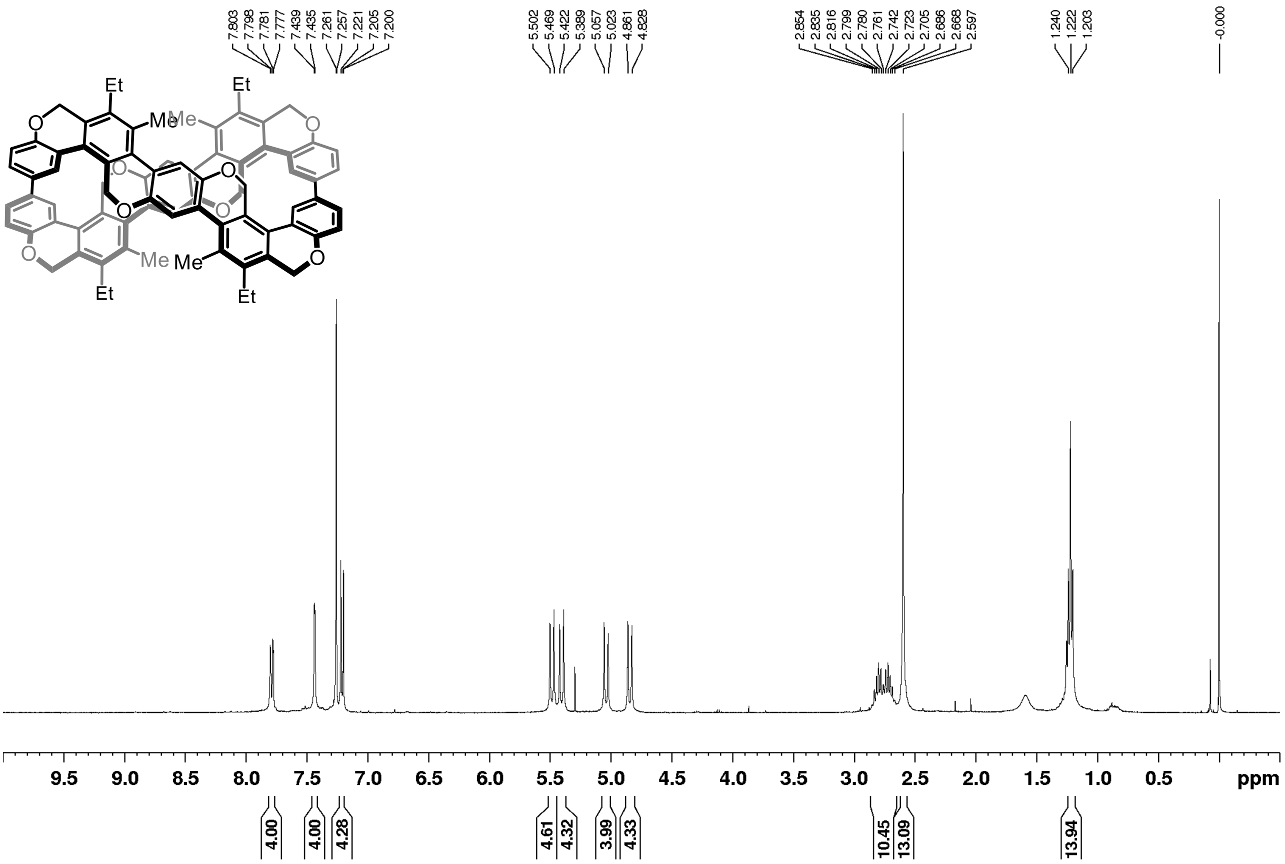

^{13}C NMR ($CDCl_3$, 100 MHz)

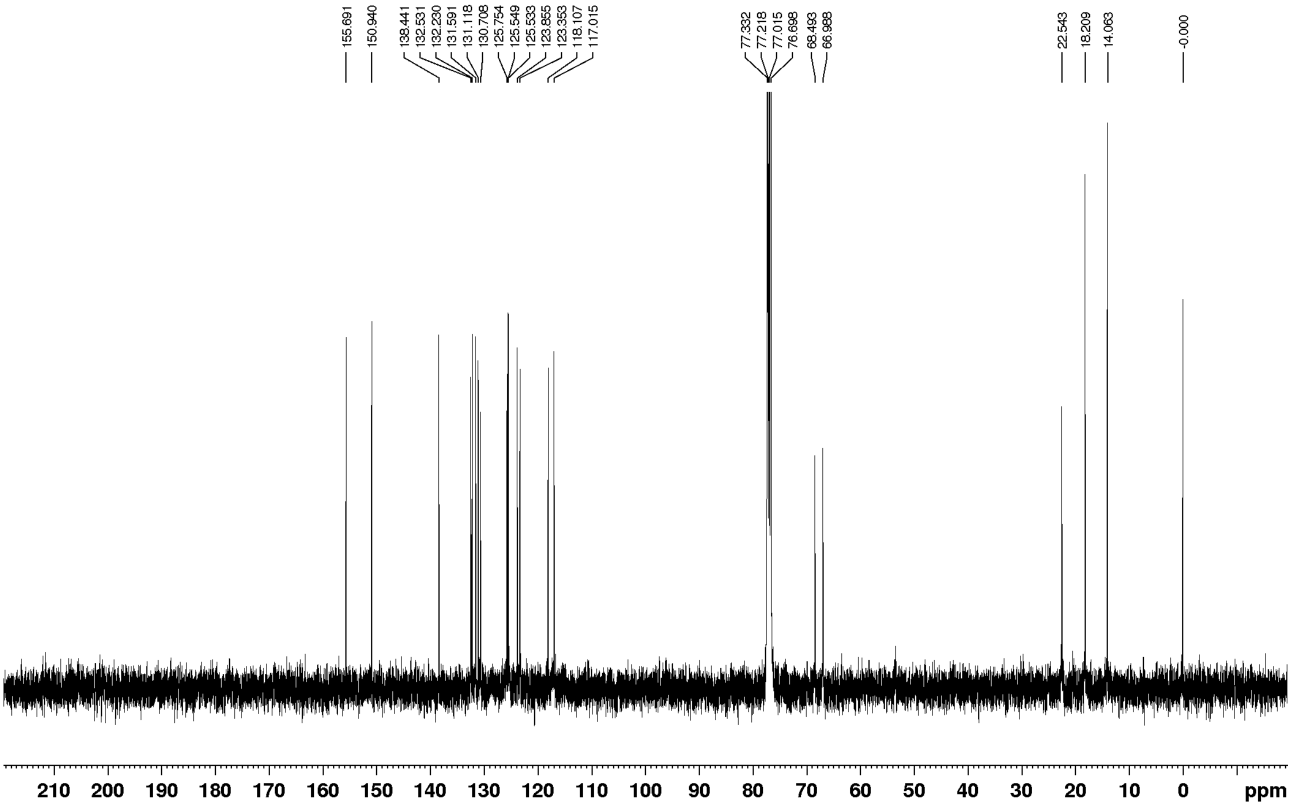

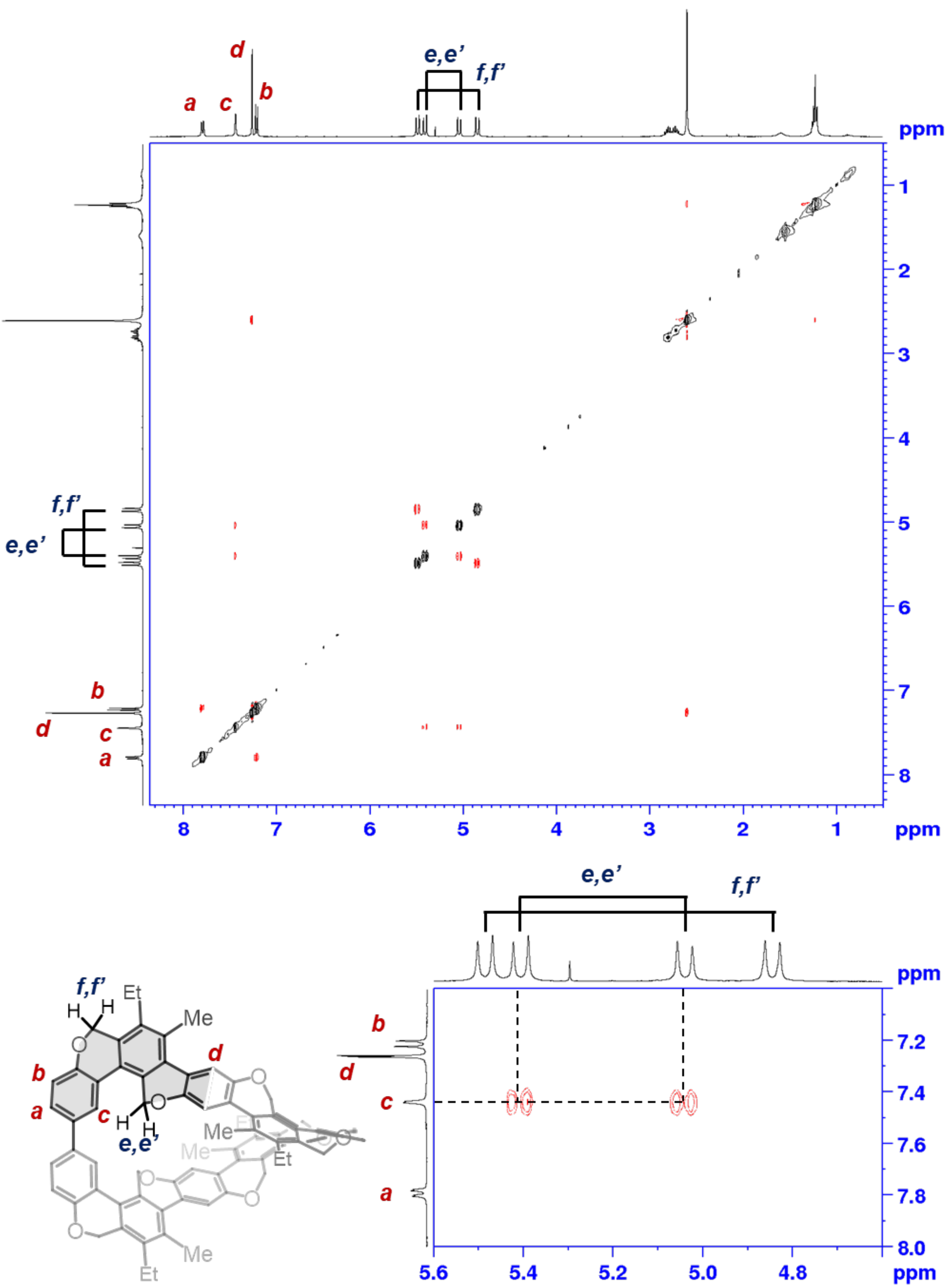

Figure S1. NOESY spectrum of **2aa** ($CDCl_3$, 400 MHz).

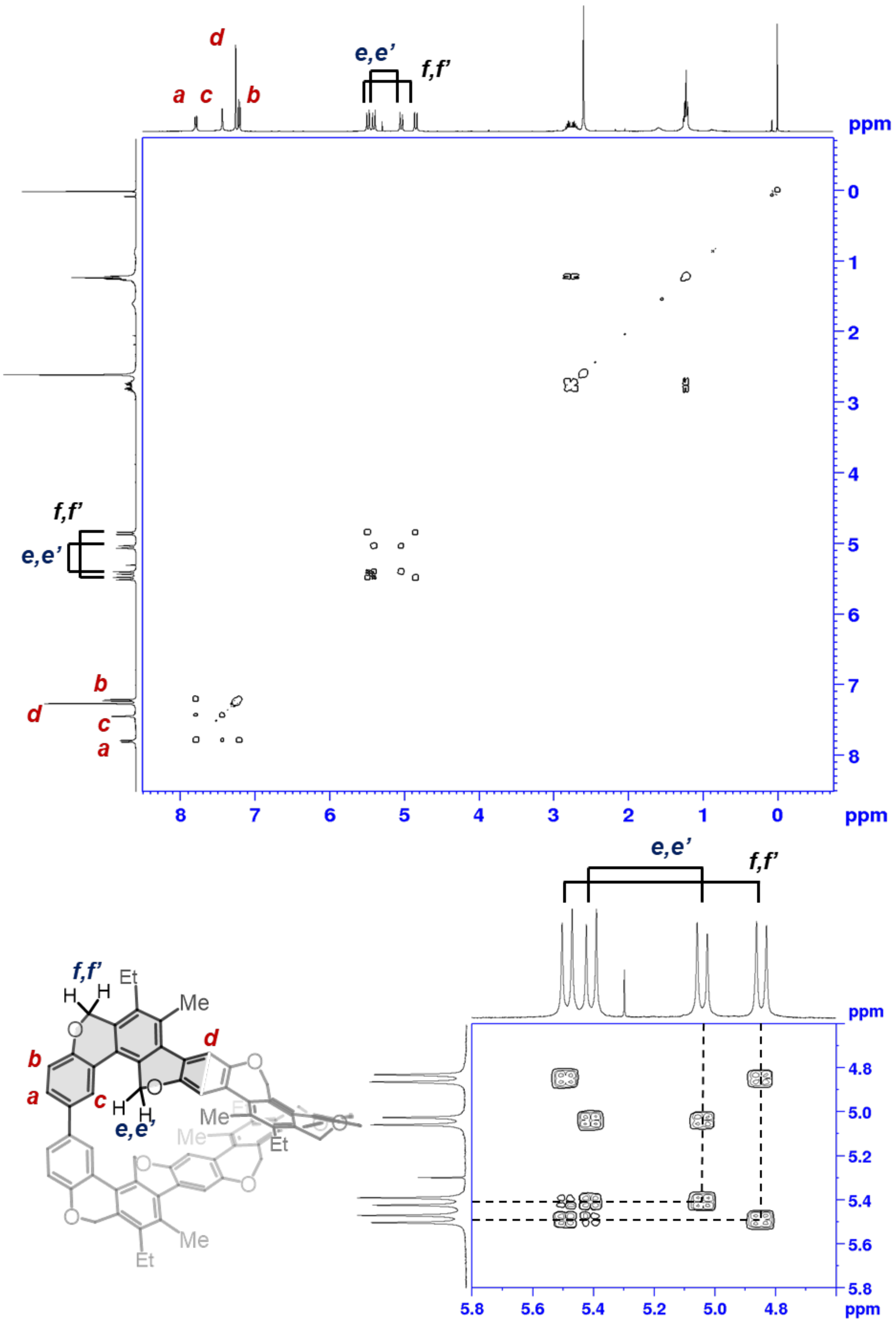

Figure S2. COSY spectrum of **2aa** ($CDCl_3$, 400 MHz).

Figure-eight molecule 2ab

^{1}H NMR ($CDCl_3$, 400 MHz)

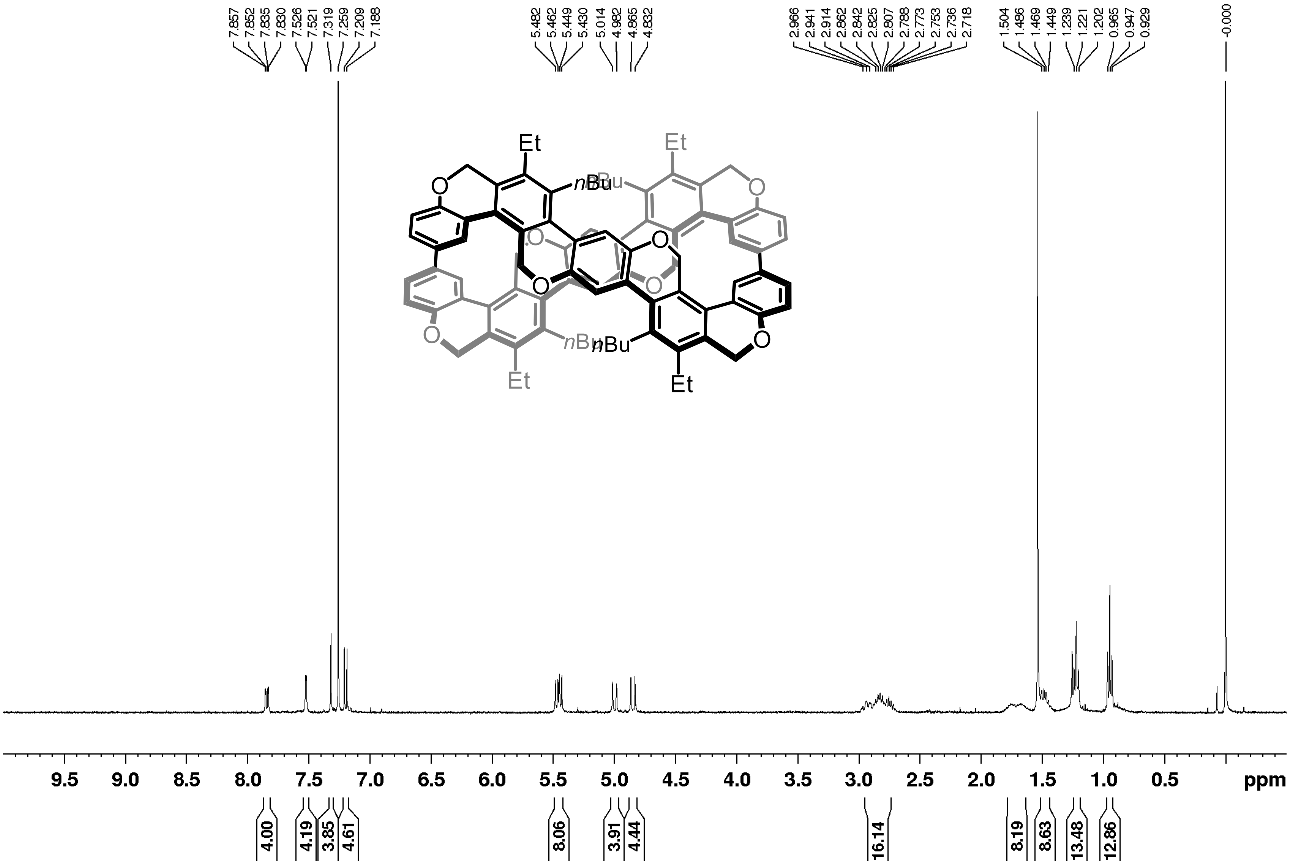

^{13}C NMR ($CDCl_3$, 100 MHz)

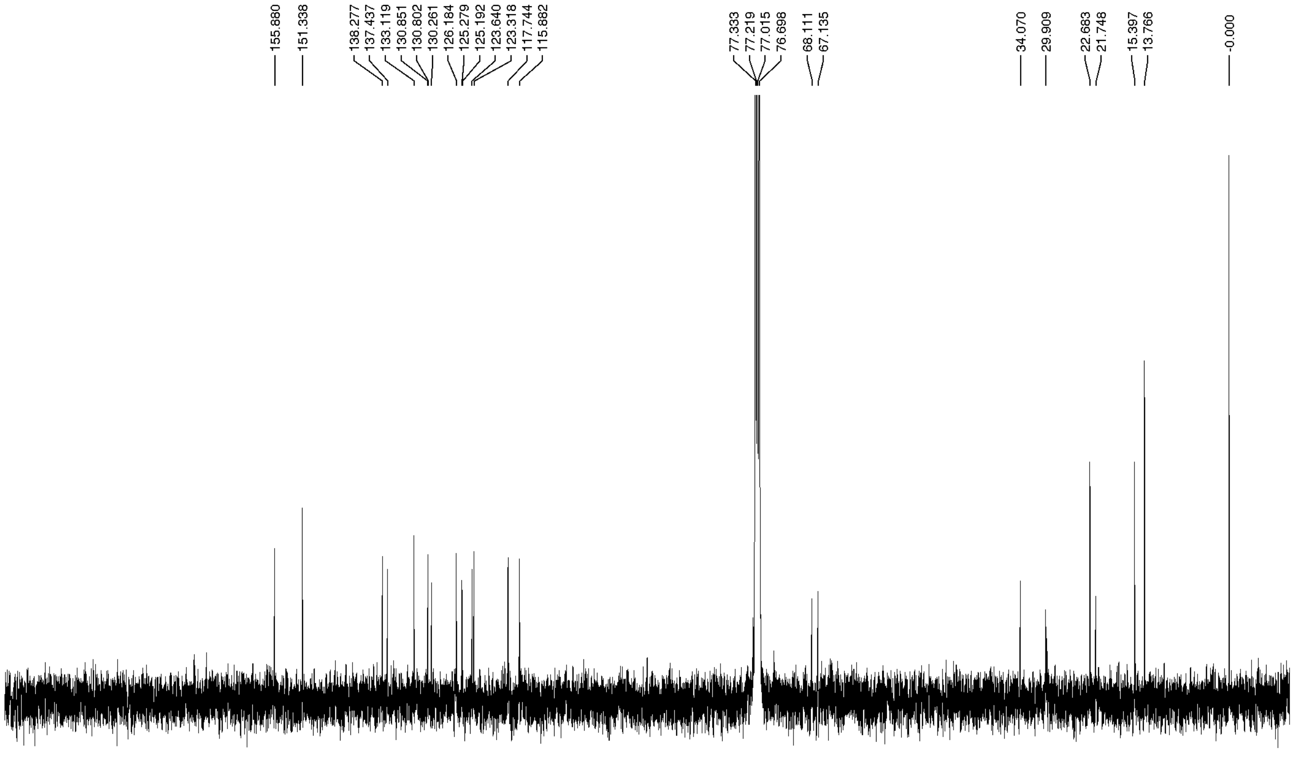

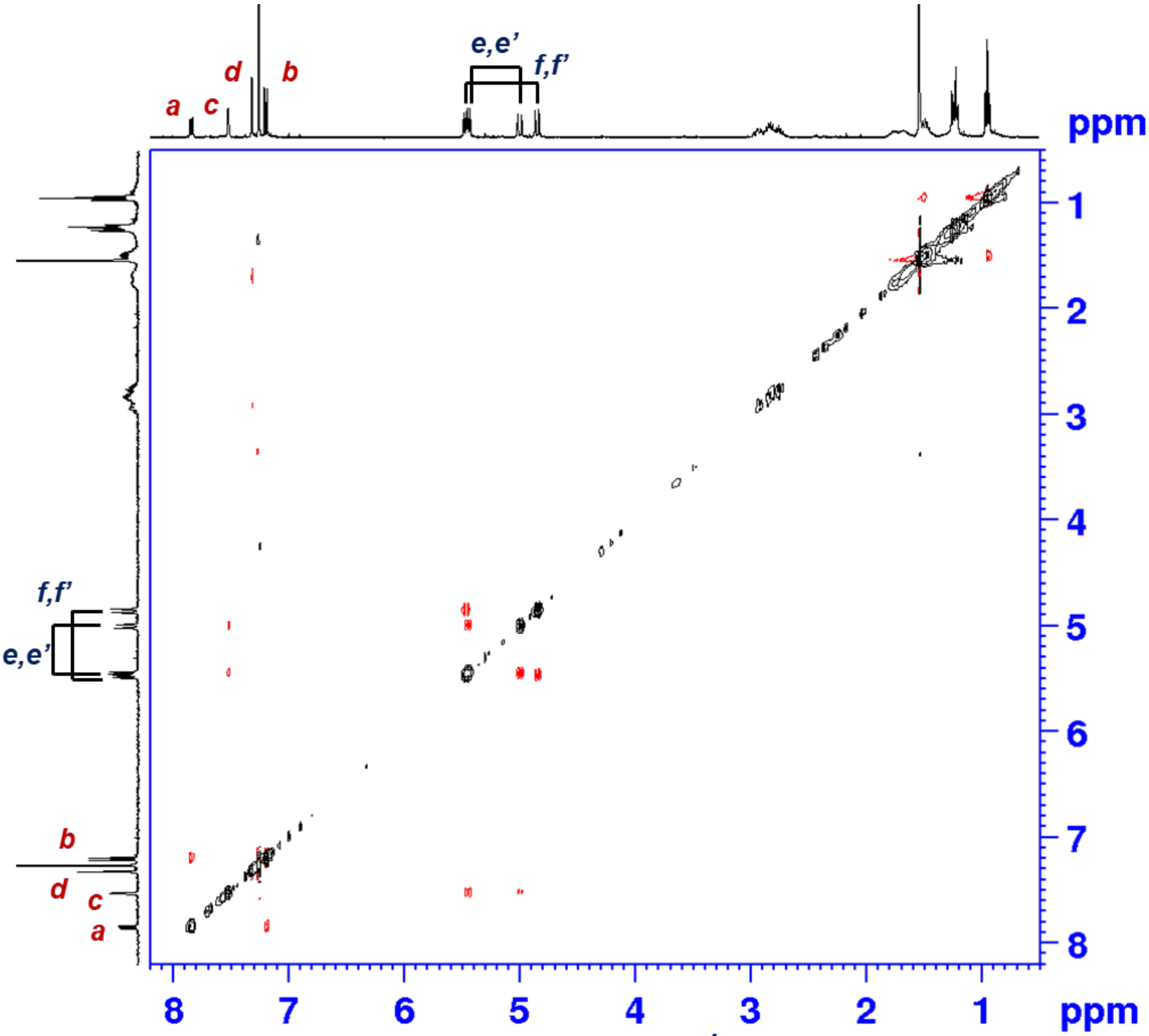

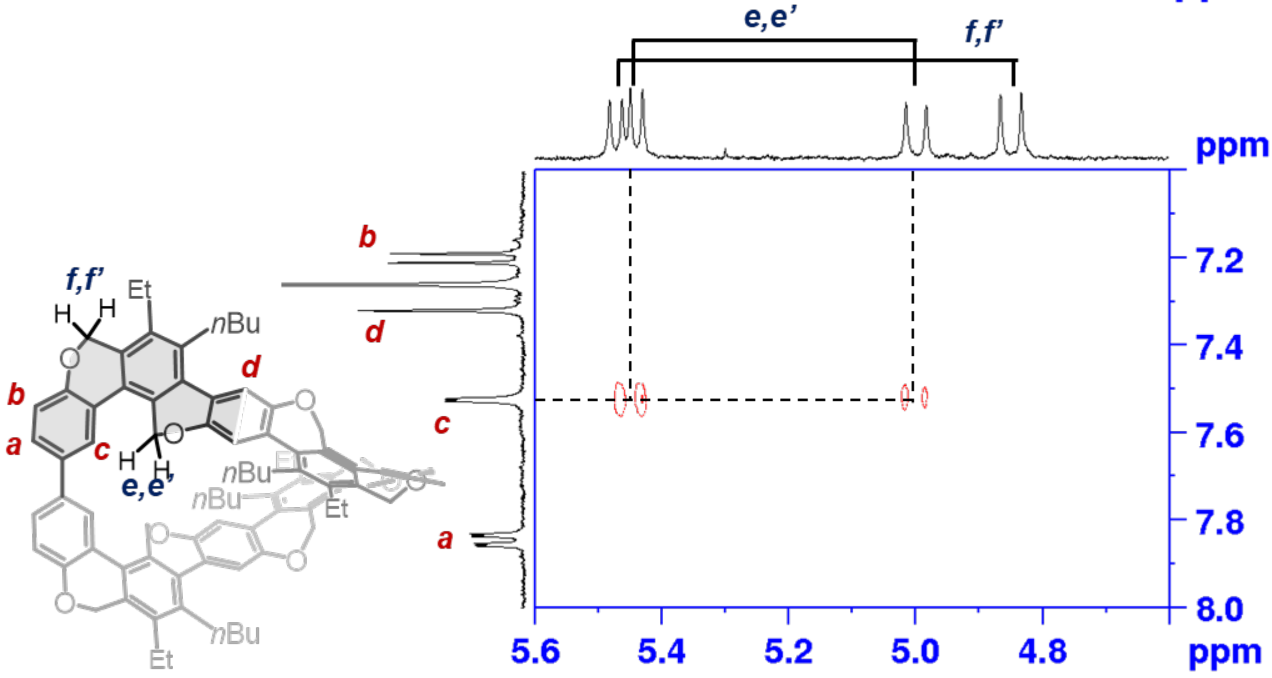

Figure S3. NOESY spectrum of **2ab** ($CDCl_3$, 400 MHz).

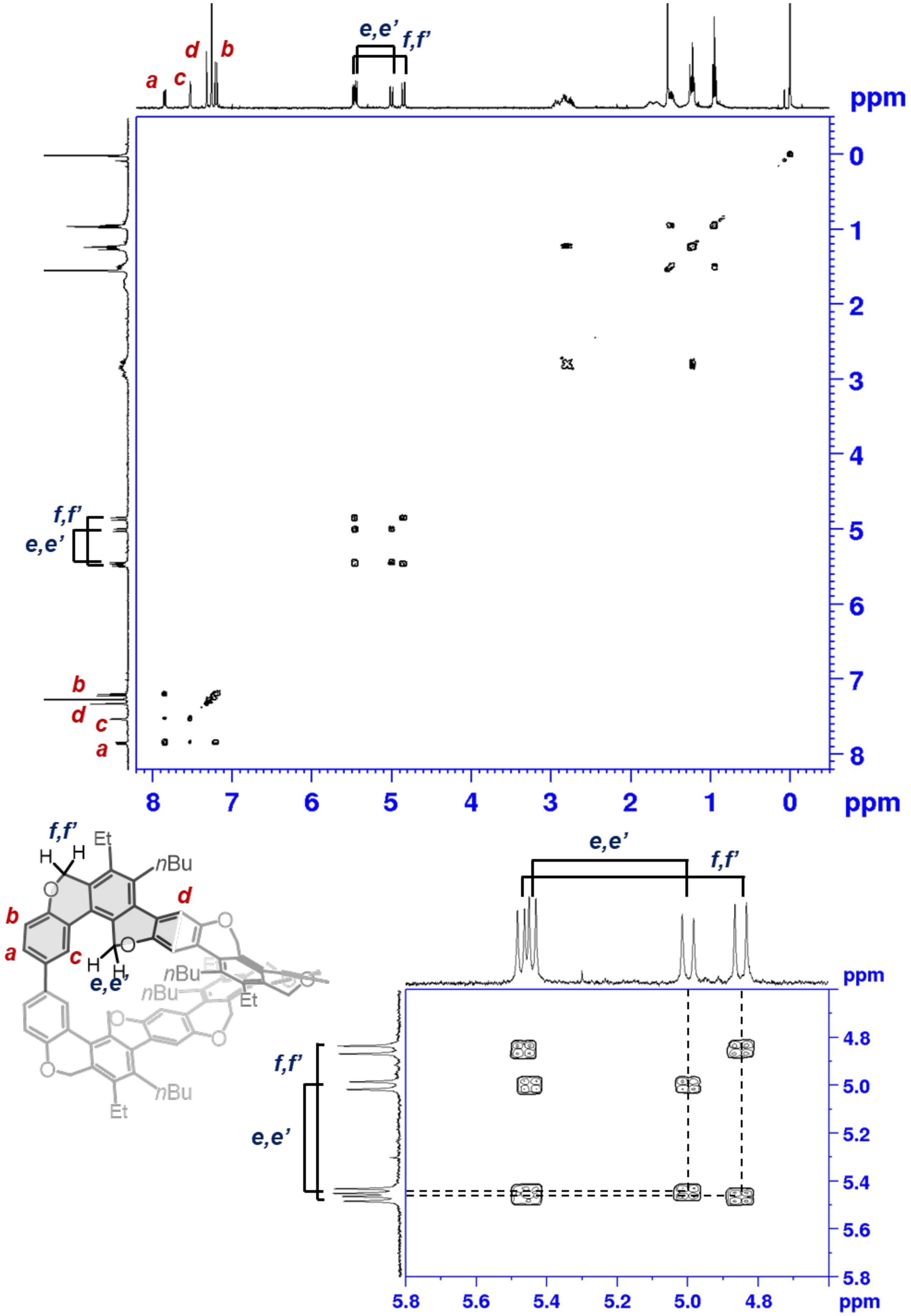

Figure S4. COSY spectrum of **2ab** ($CDCl_3$, 400 MHz).

Figure-eight molecule 1ba

^{1}H NMR ($CDCl_3$, 400 MHz)

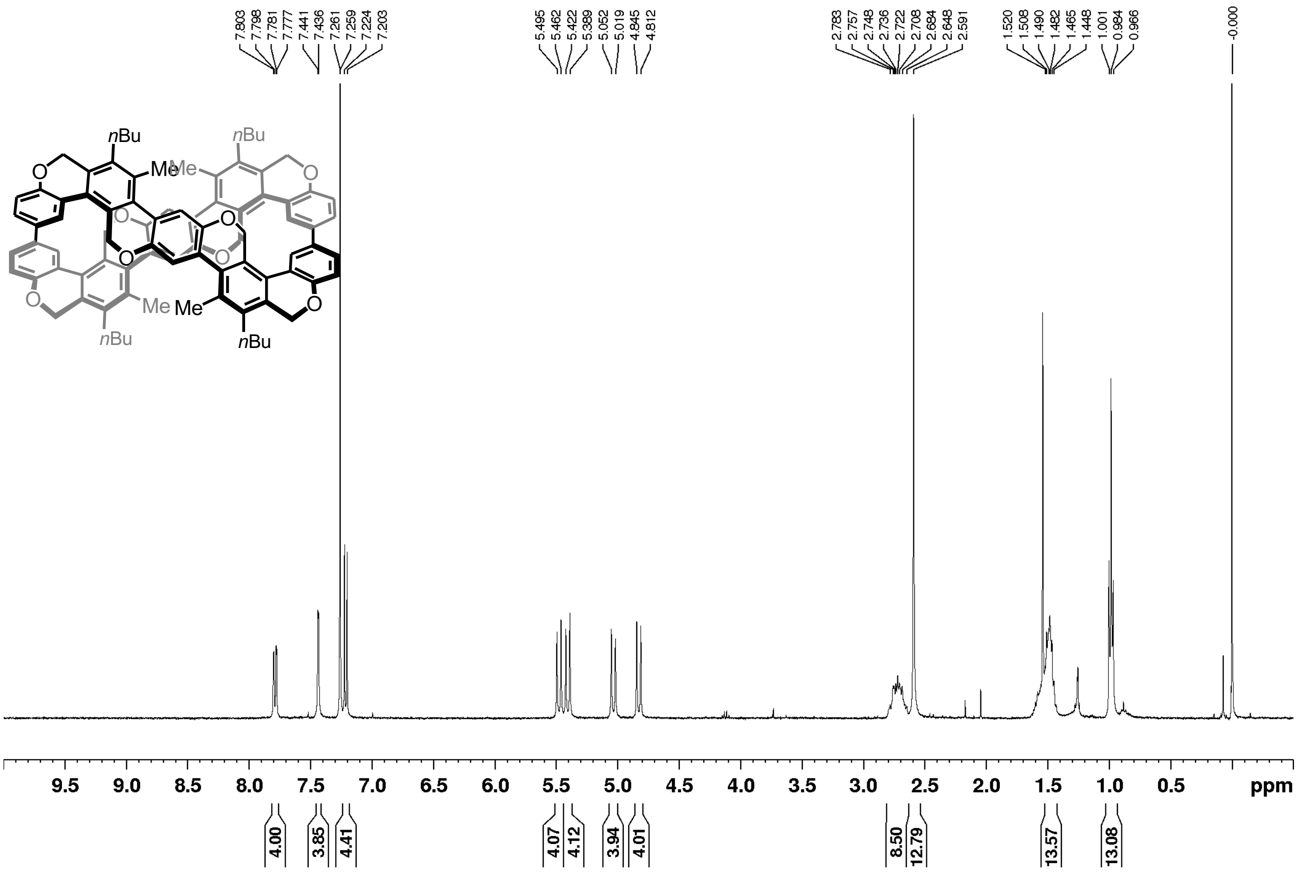

^{13}C NMR ($CDCl_3$, 100 MHz)

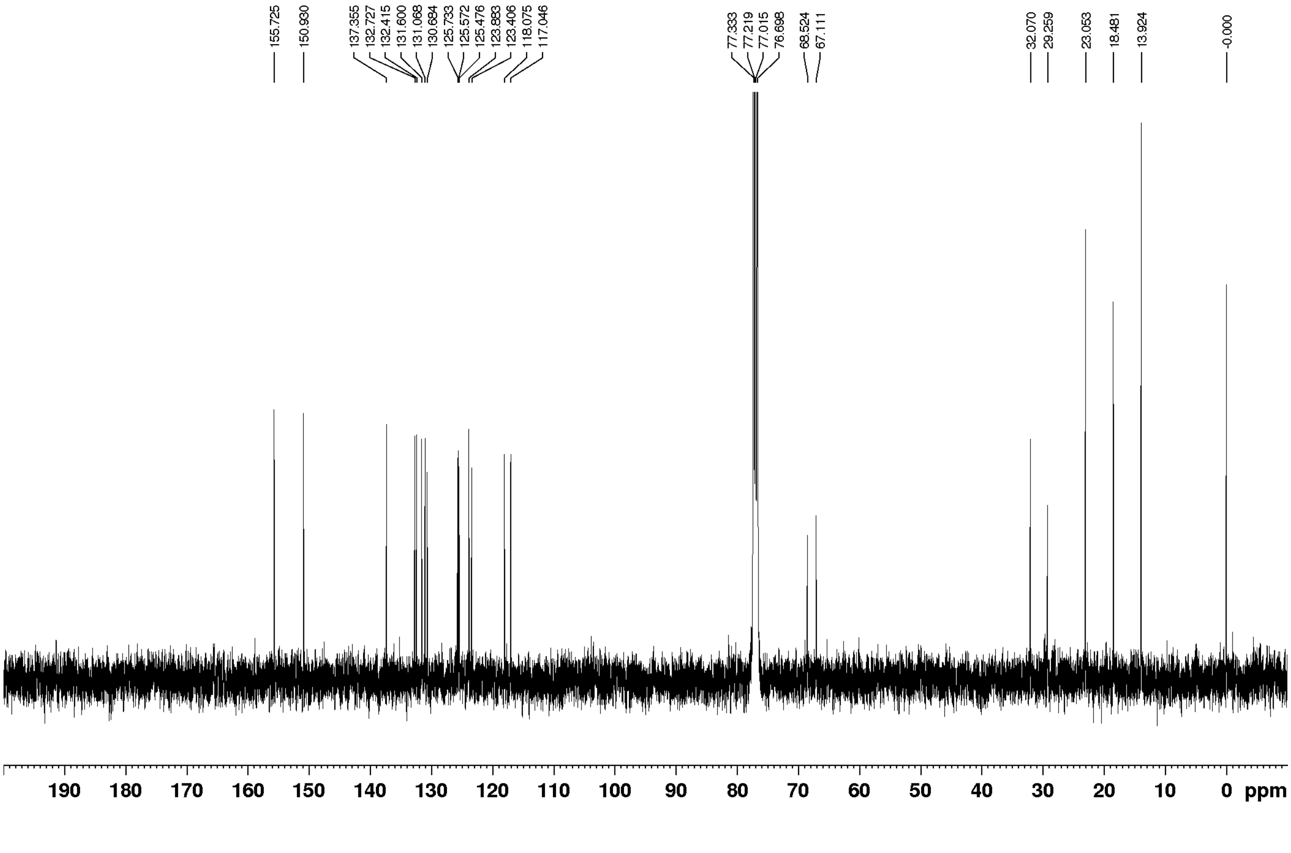

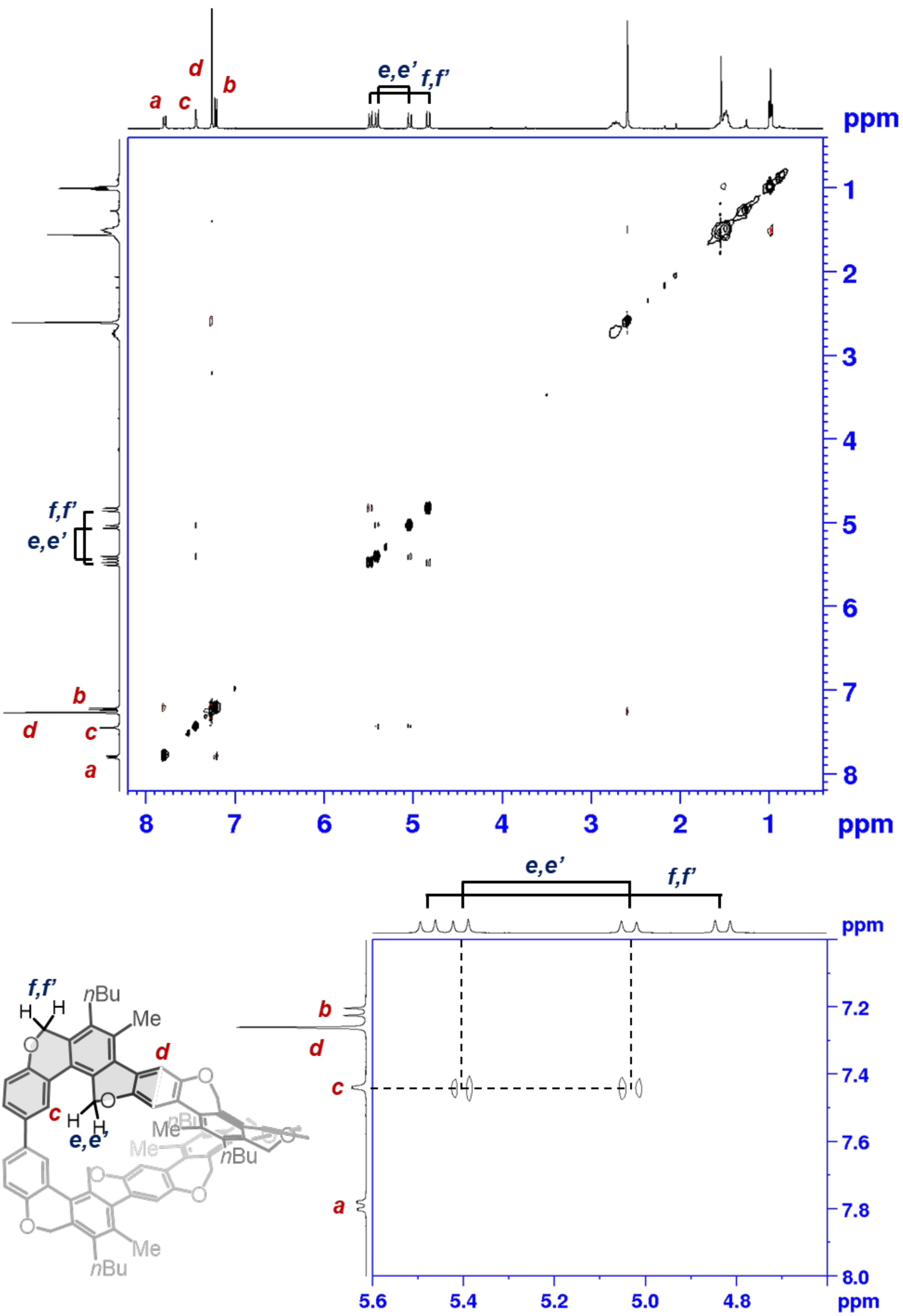

Figure S5. NOESY spectrum of **2ba** ($CDCl_3$, 400 MHz).

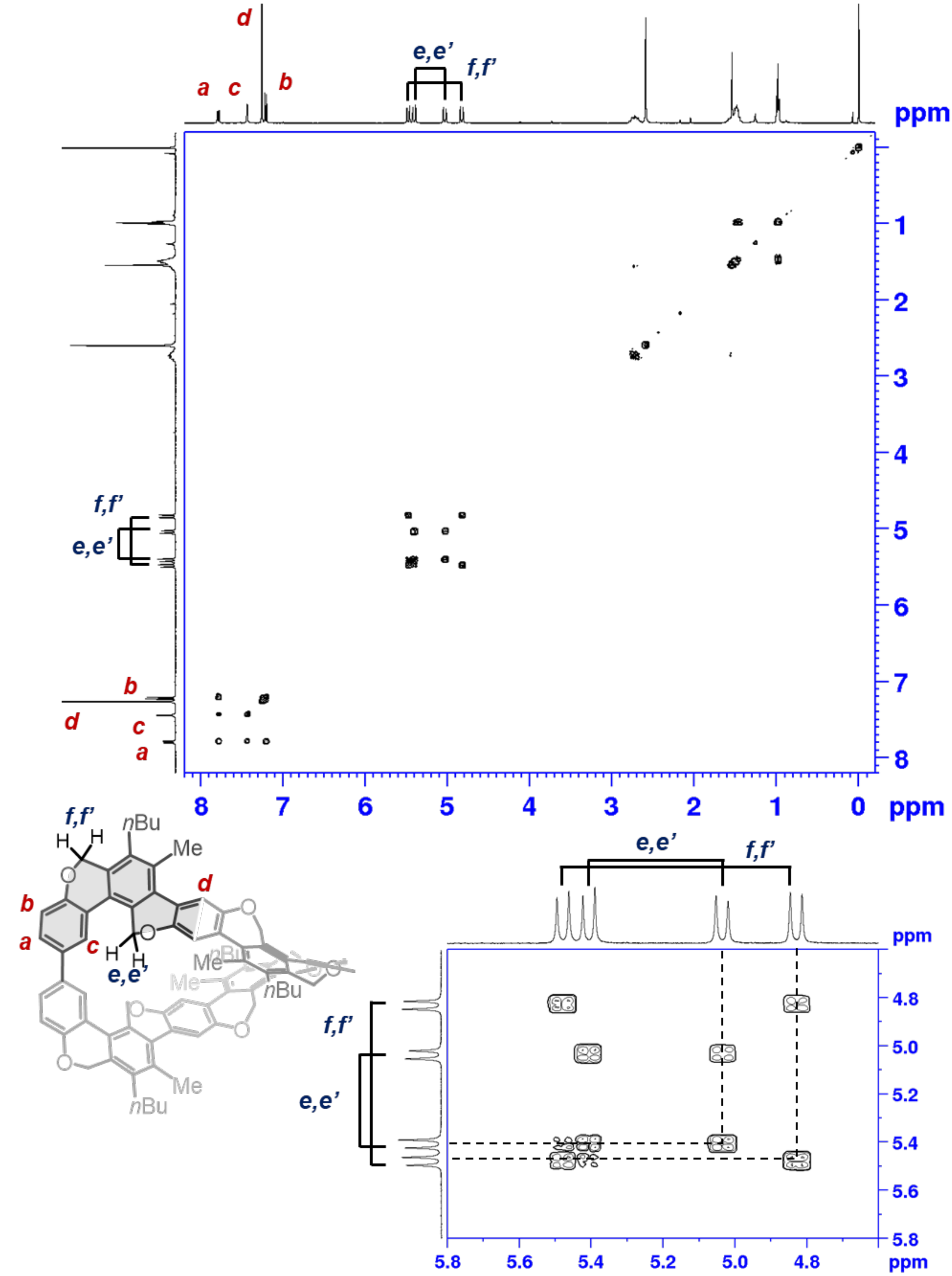

Figure S6. COSY spectrum of **2ba** ($CDCl_3$, 400 MHz).

Intermediate 10

^{1}H NMR ($CDCl_3$, 400 MHz)

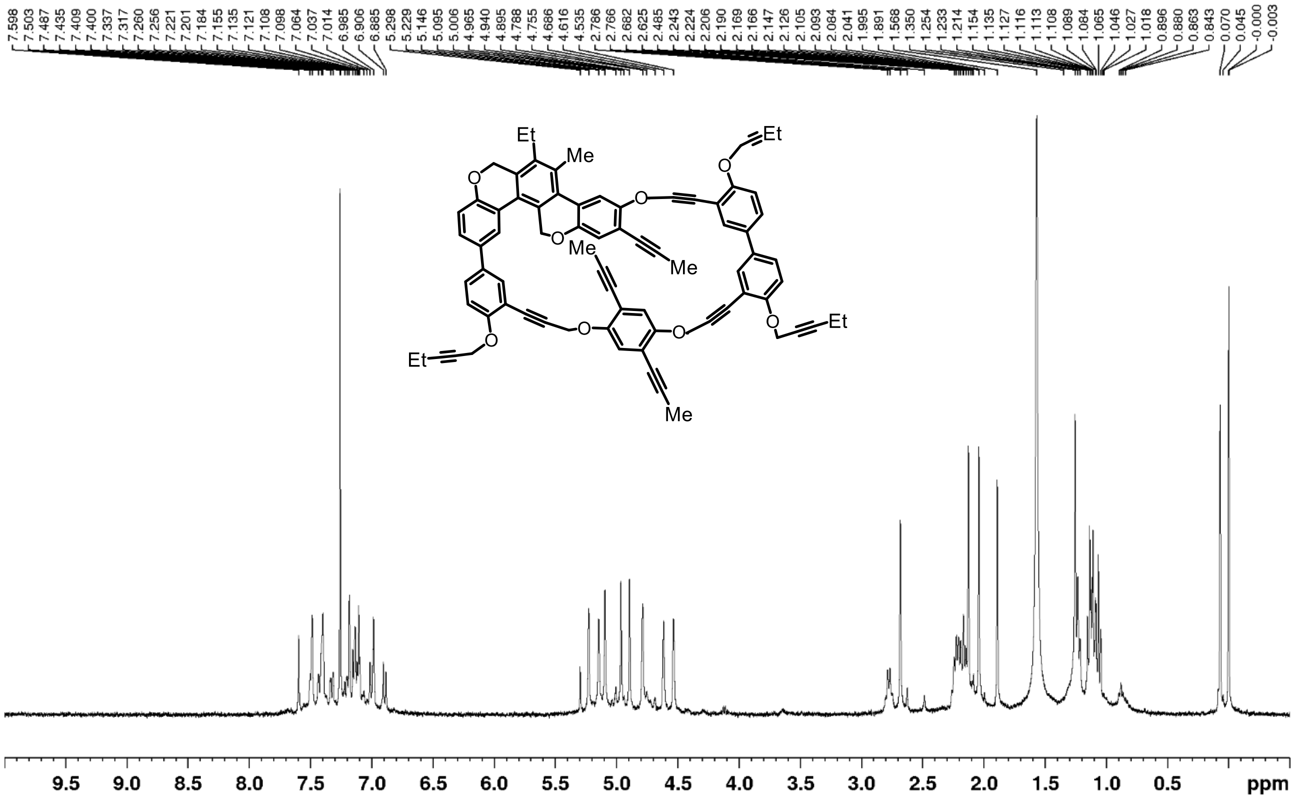

^{13}C NMR ($CDCl_3$, 125 MHz)

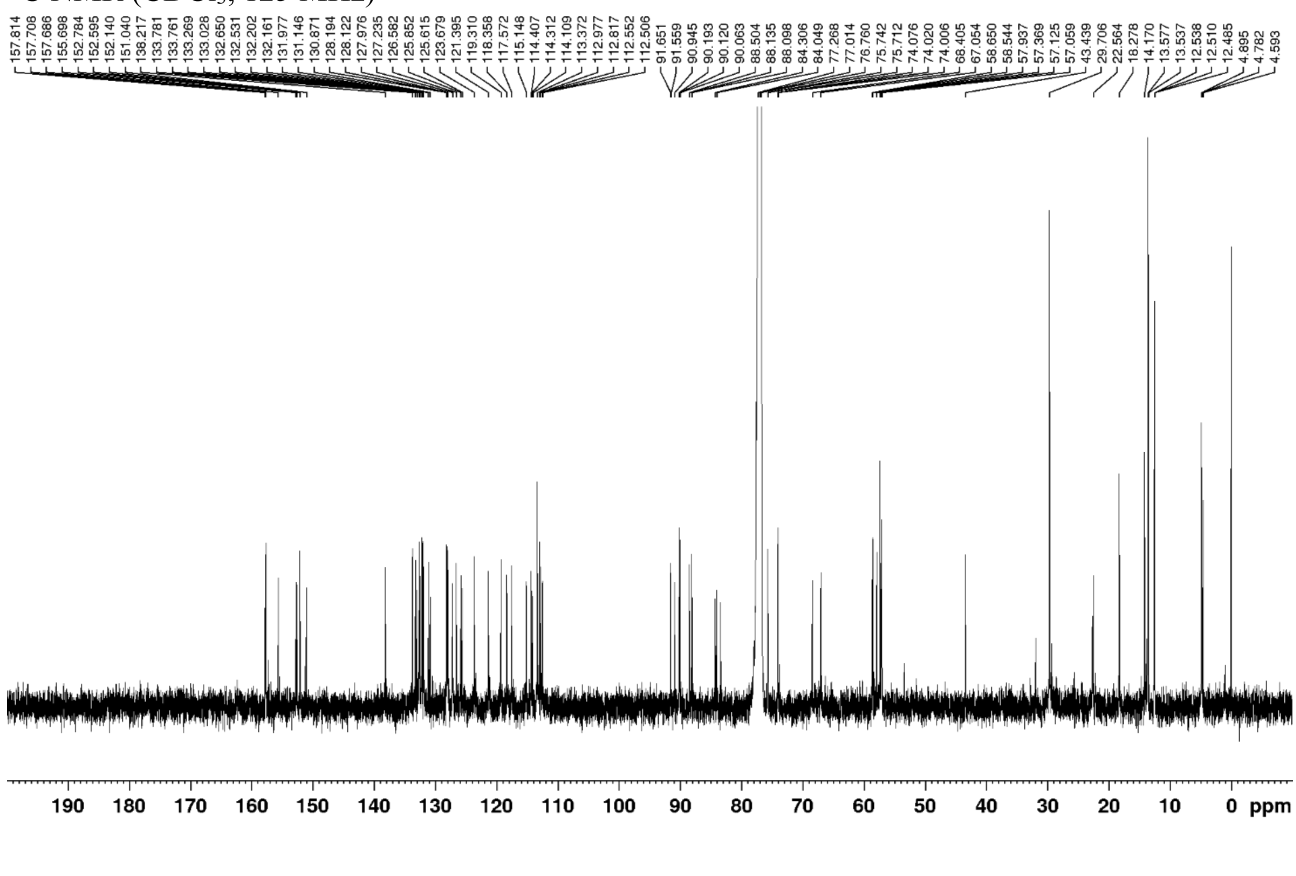

Intermediate 11

^1H NMR ($CDCl_3$, 400 MHz)

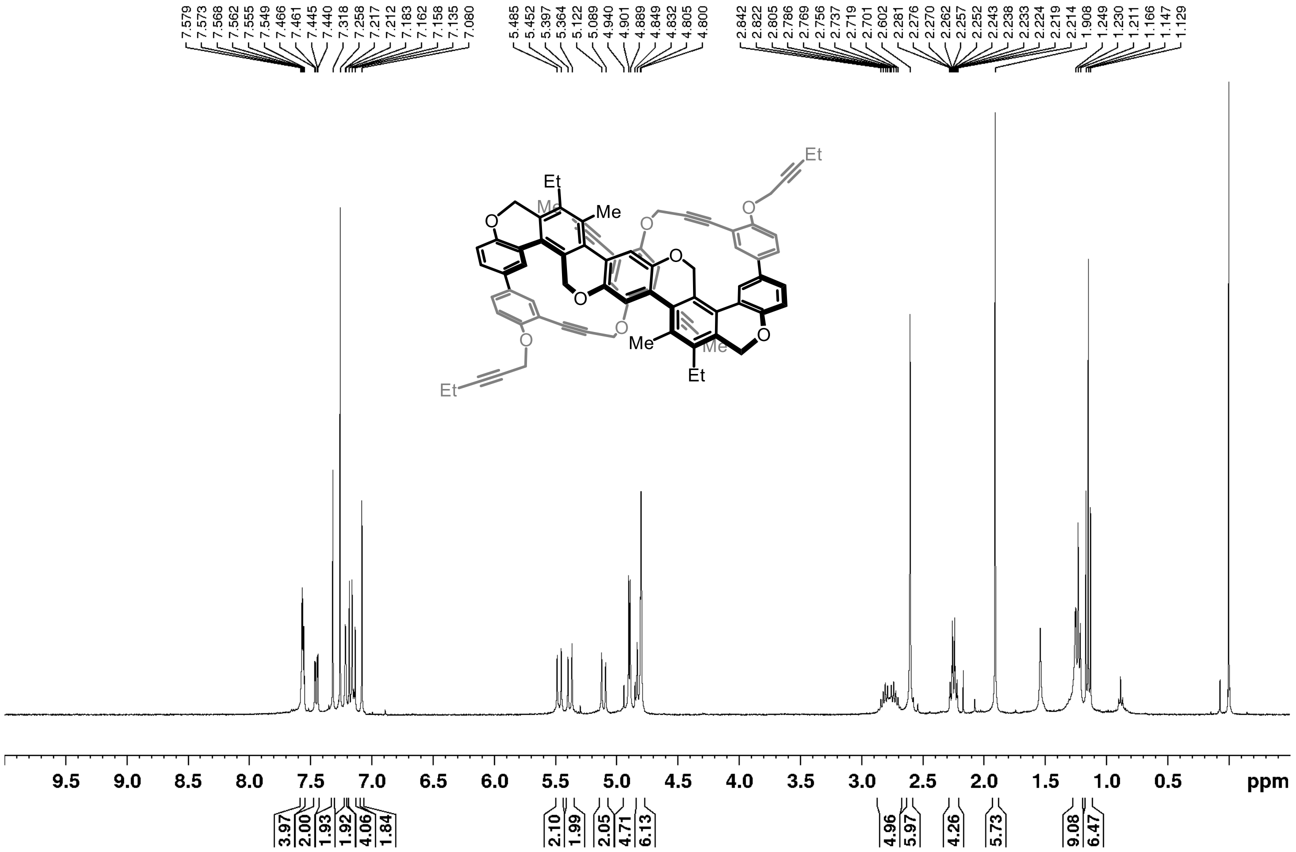

^{13}C NMR ($CDCl_3$, 100 MHz)

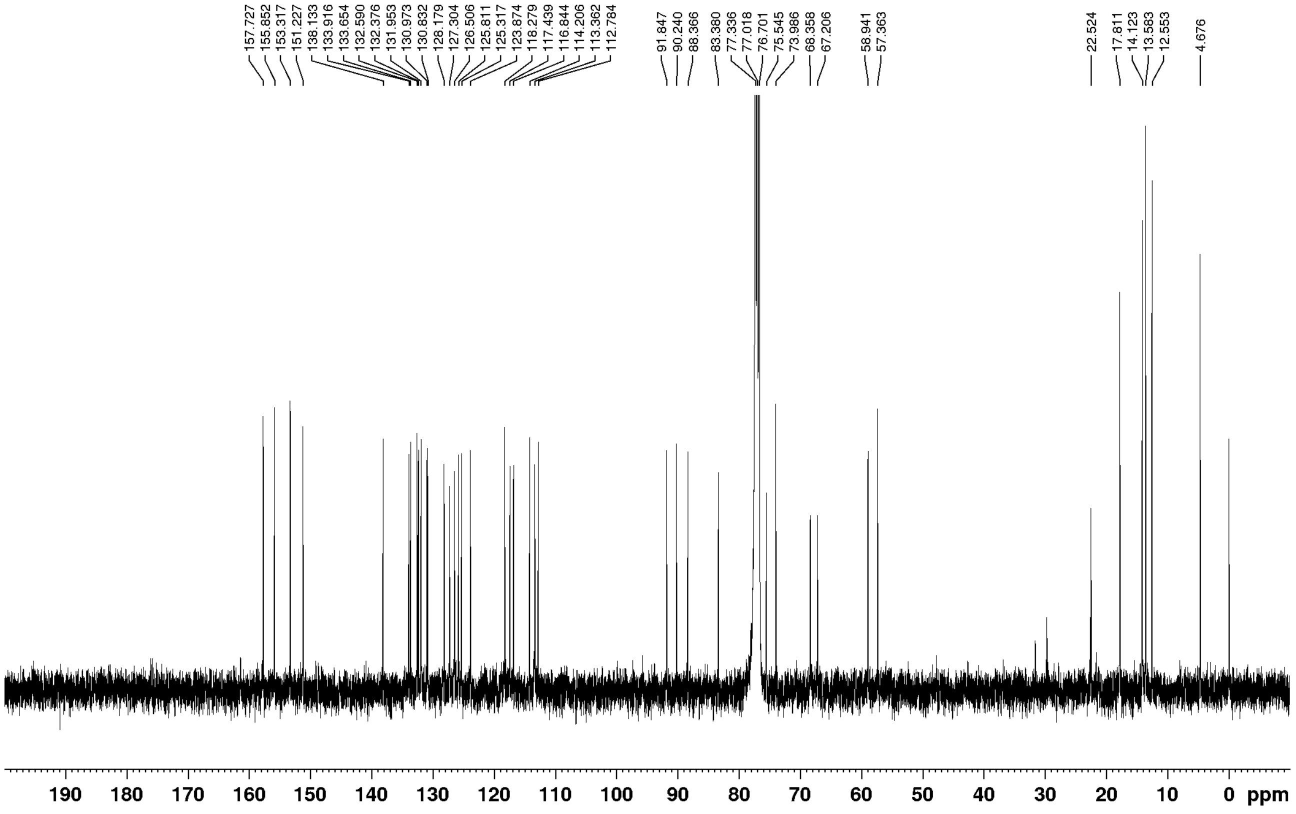

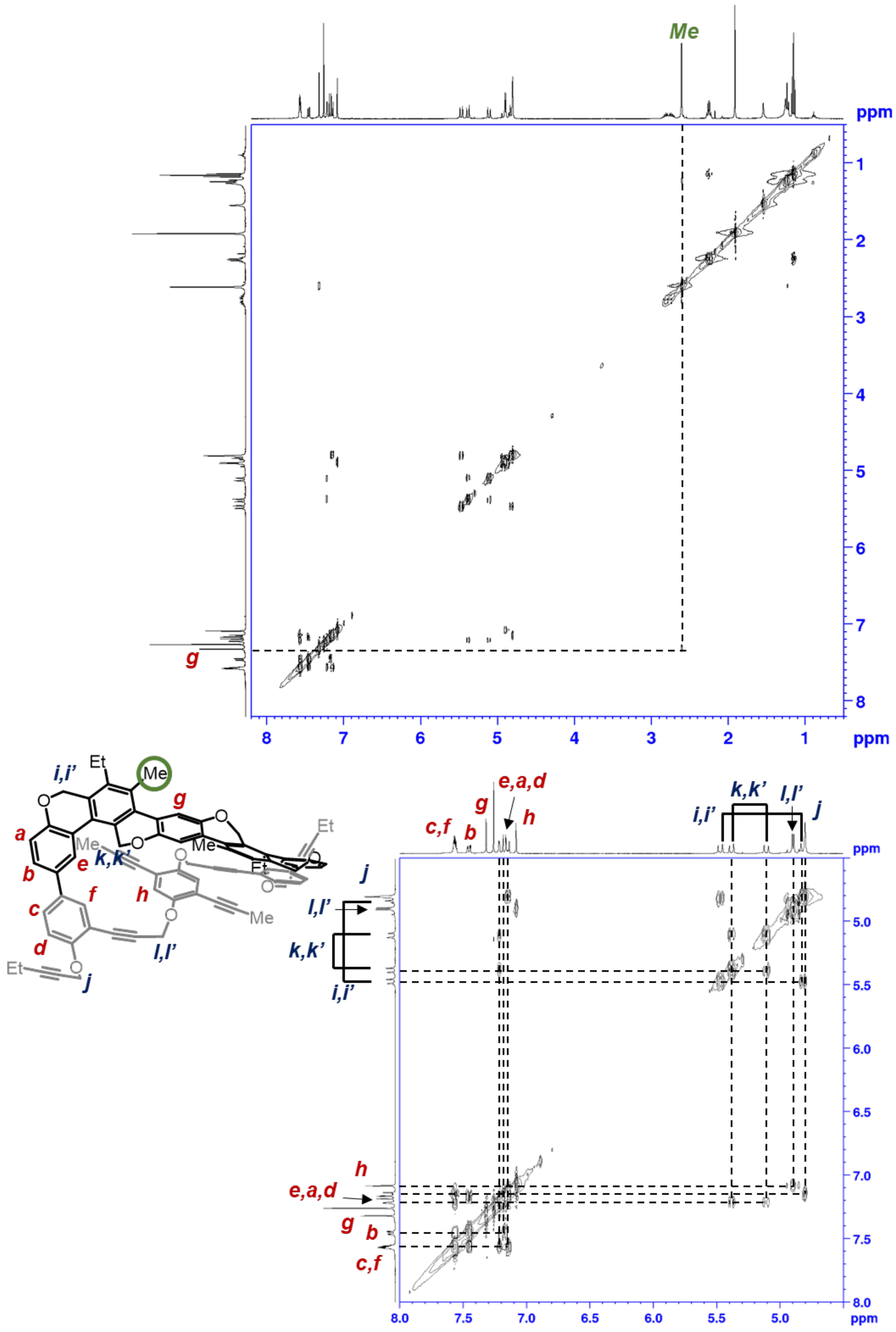

Figure S7. NOESY spectrum of **11** ($CDCl_3$, 400 MHz)

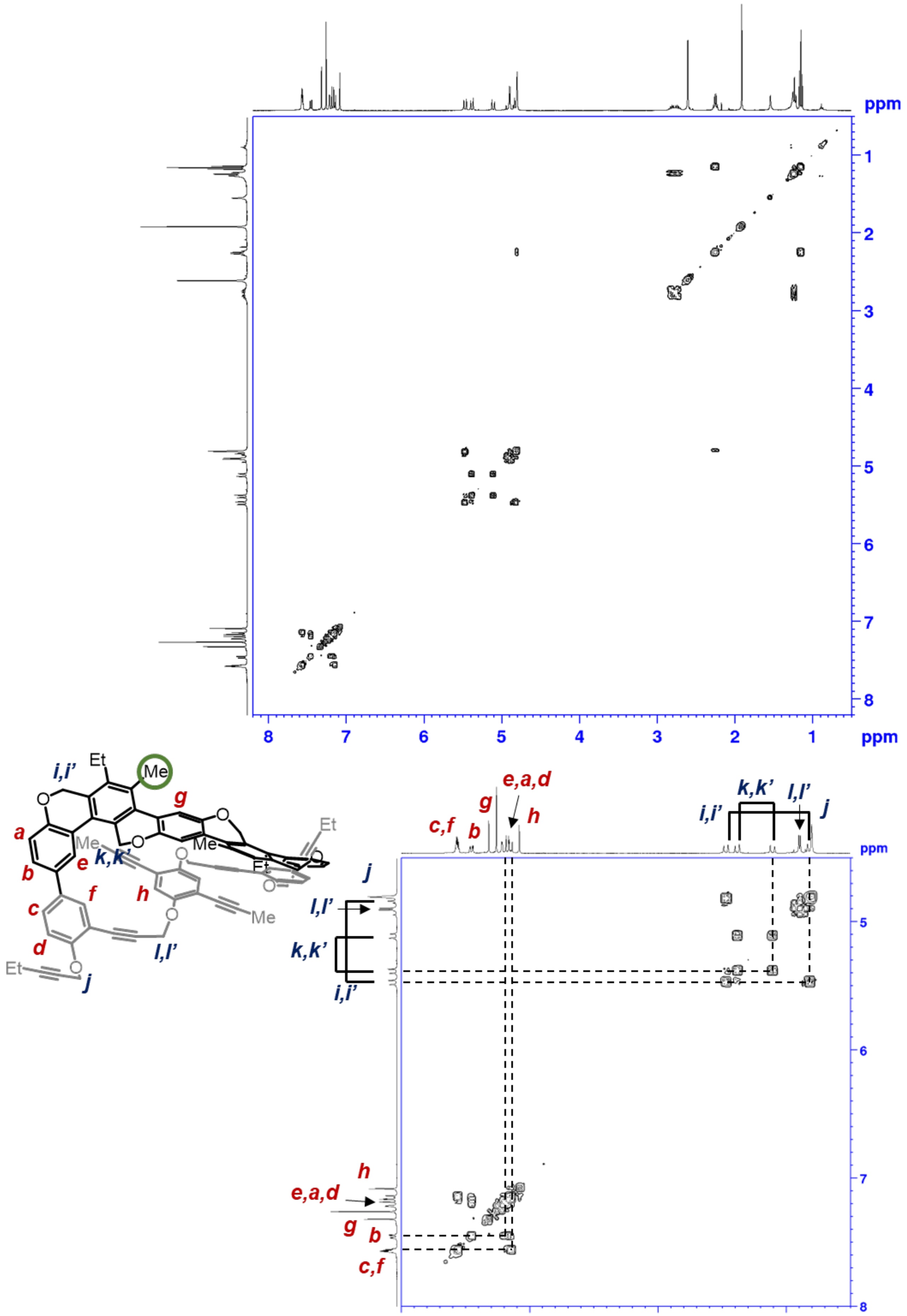

Figure S8. COSY spectrum of **11** ($CDCl_3$, 400 MHz)

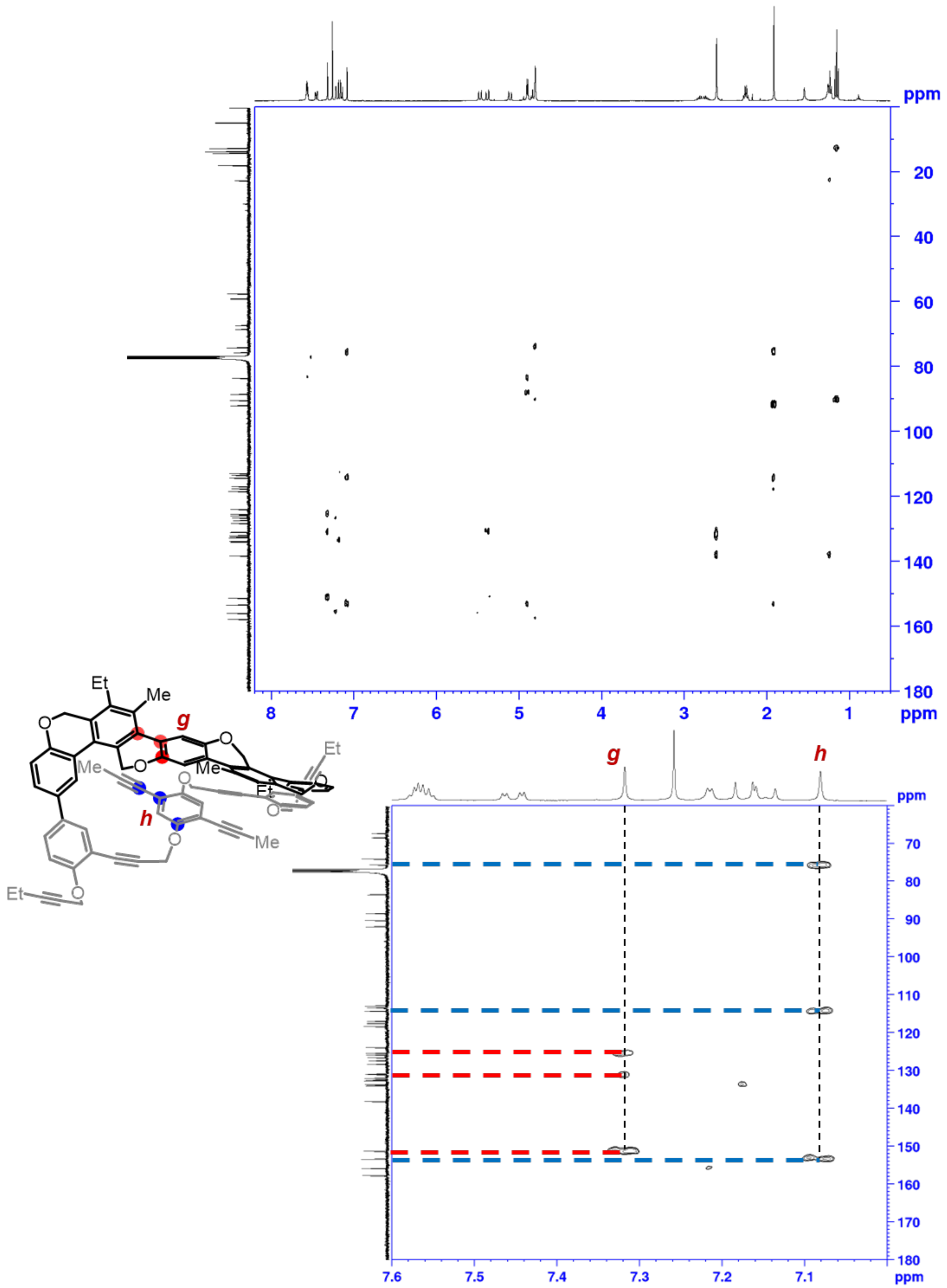

Figure S9. HMBC spectrum of **11** ($CDCl_3$, 400 MHz)

Mixture of intermediates 12 and 13 (mol-A/mol-B = 1:1.2, undetermined whether mol-A or mol-B is 12 or 13)

^{1}H NMR ($CDCl_3$, 400 MHz)

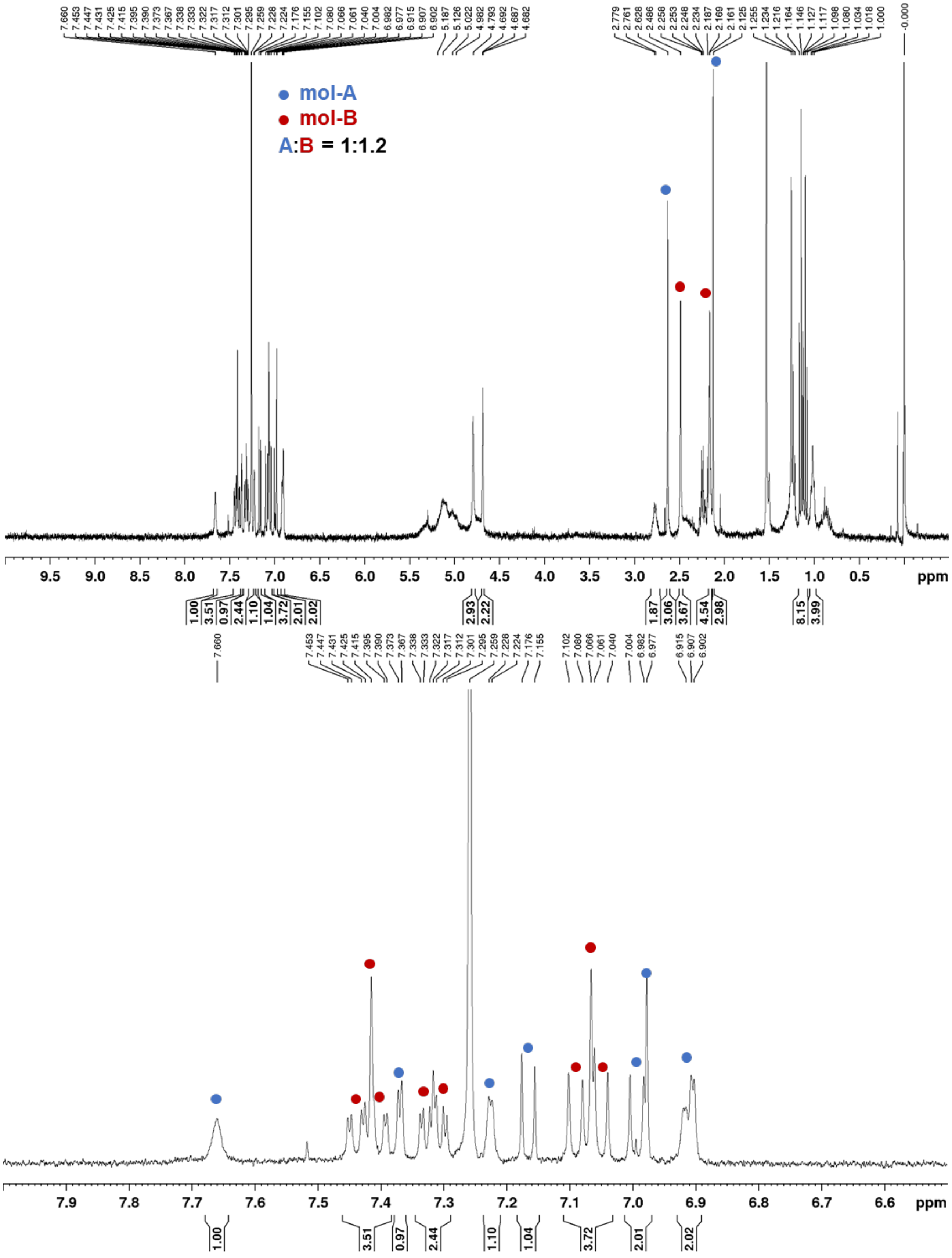

Intermediate 14

^{1}H NMR ($CDCl_3$, 600 MHz)

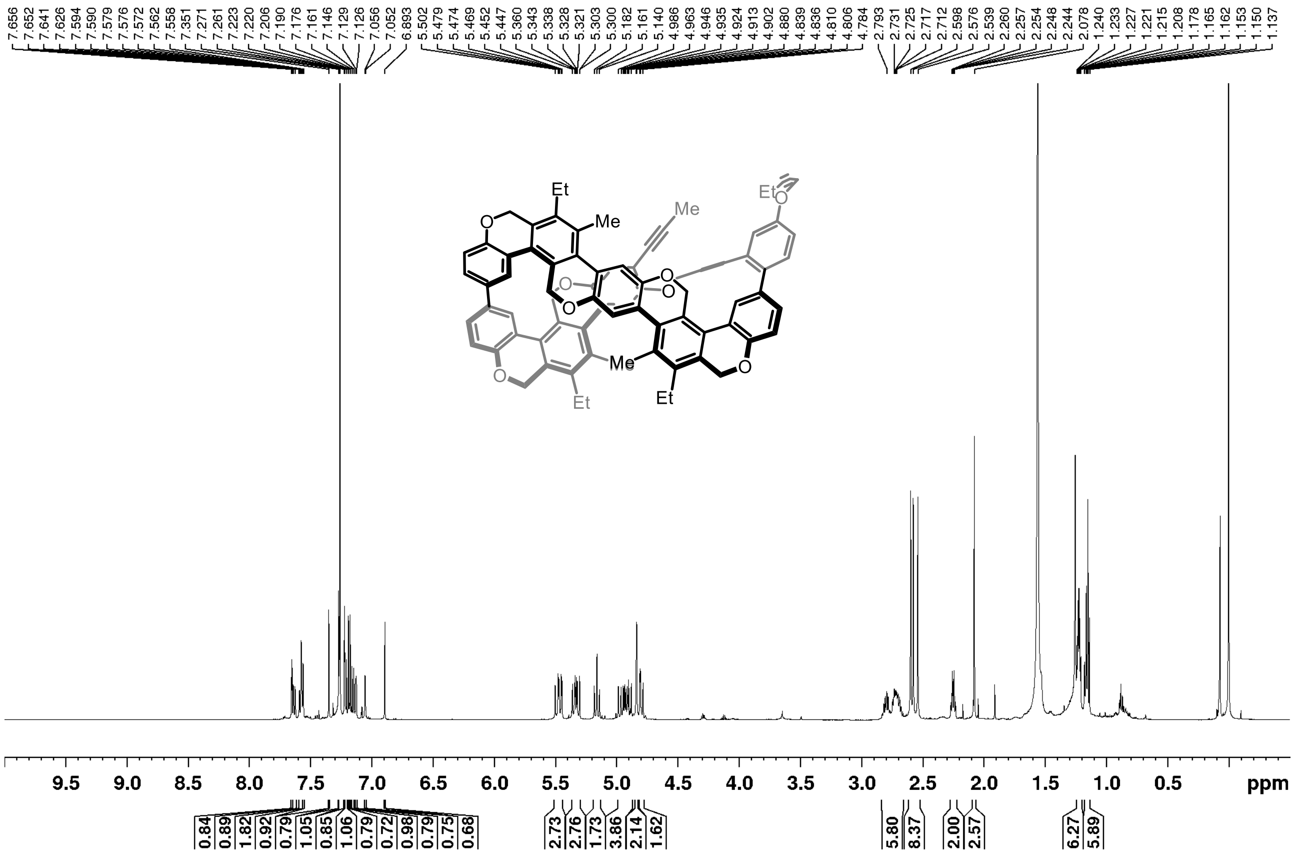

^{13}C NMR ($CDCl_3$, 150 MHz)

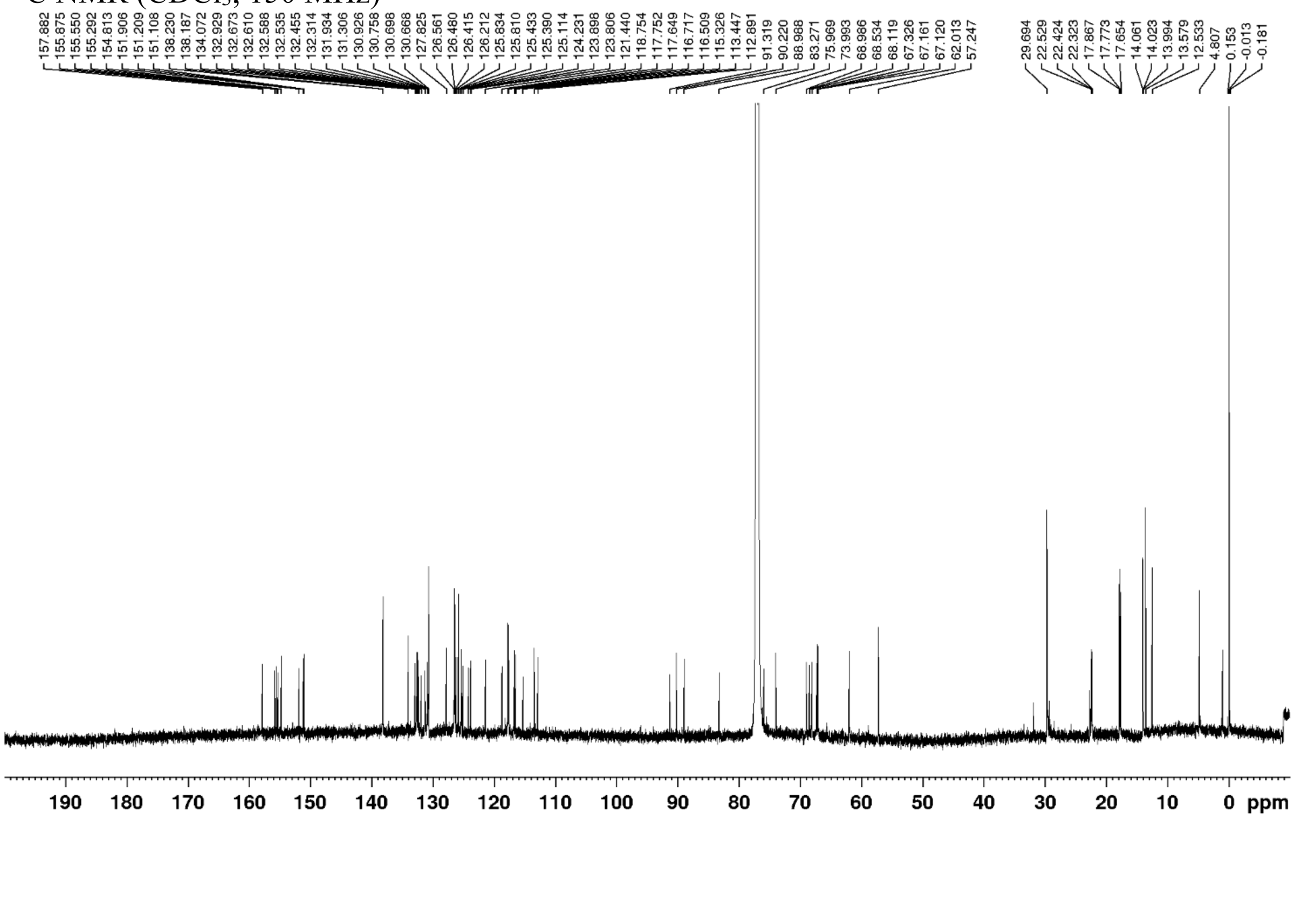

6. Chiral HPLC Charts

(±)-**2aa**
CHIRALPAK IE-3, CH_2Cl_2, 0.75 mL min^{-1}

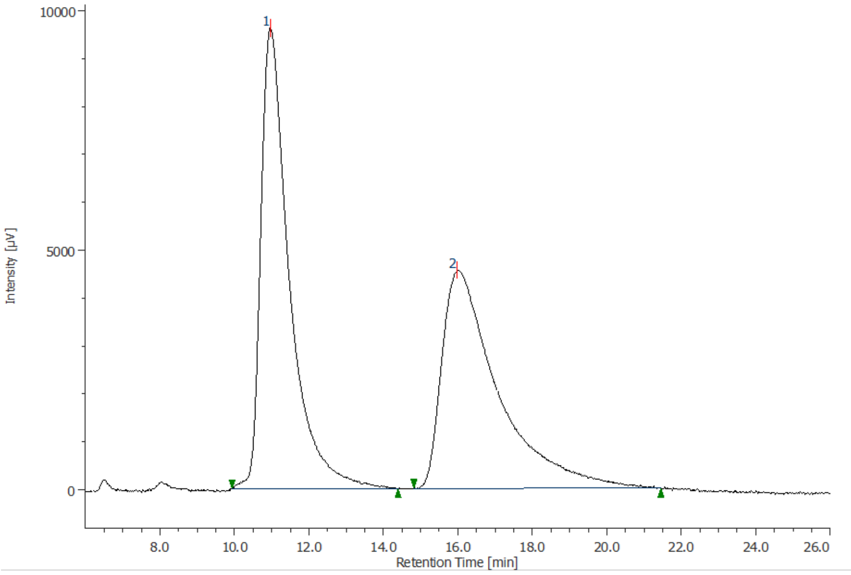

Peak No.	Retention time (min)	Area (%)
1	10.958	51.831
2	15.983	48.169

(*M,M*)-(–)-**2aa**
CHIRALPAK IE-3, CH_2Cl_2, 0.75 mL min^{-1}

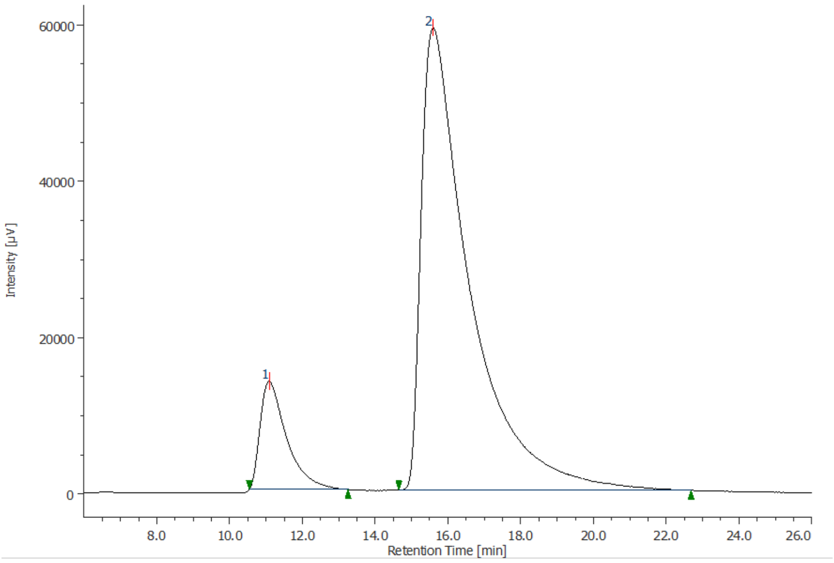

Peak No.	Retention time (min)	Area (%)
1	11.083	11.314
2	15.592	88.686

(±)-**2ab**

CHIRALPAK IE-3, *n*-hexane/CH_2Cl_2 = 30:70, 1.0 mL min^{-1}

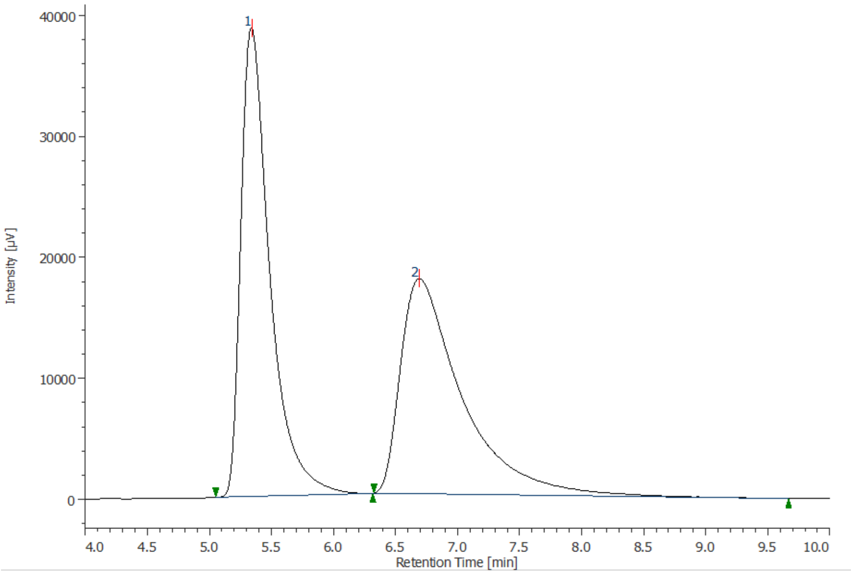

Peak No.	Retention time (min)	Area (%)
1	5.342	50.679
2	6.692	49.321

(*M,M*)-(–)-**2ab**

CHIRALPAK IE-3, n-hexane/CH_2Cl_2 = 30:70, 1.0 mL min^{-1}

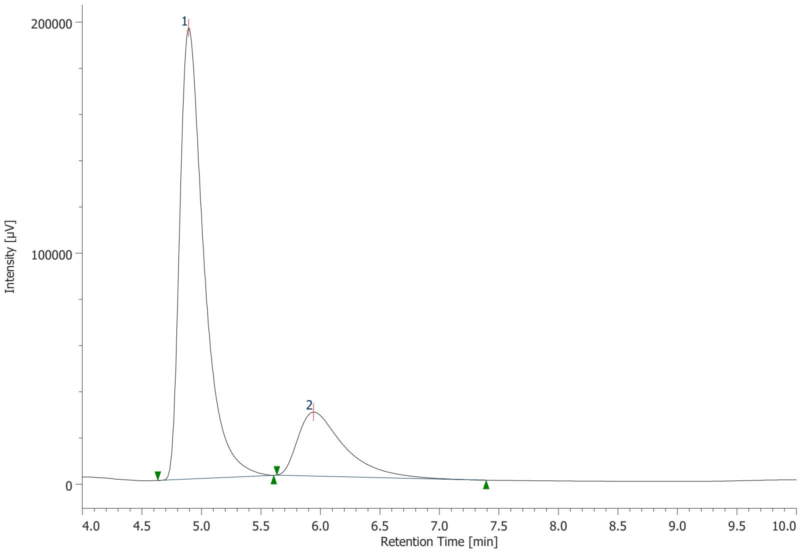

Peak No.	Retention time (min)	Area (%)
1	4.892	78.645
2	5.942	21.355

(±)-**2ba**
CHIRALPAK IE-3, CH_2Cl_2, 1.0 mL min^{-1}

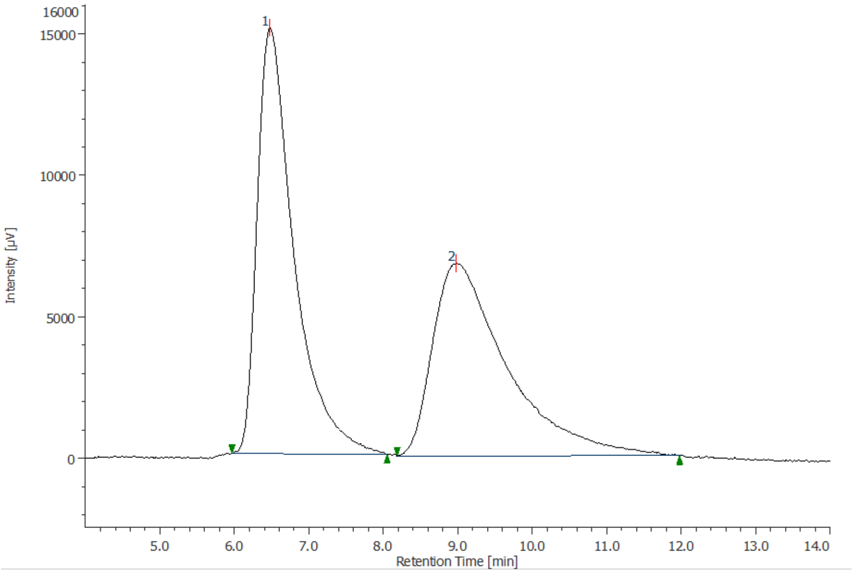

Peak No.	Retention time (min)	Area (%)
1	6.475	52.352
2	8.975	47.648

(*M*,*M*)-(–)-**2ba**
CHIRALPAK IE-3, CH_2Cl_2, 1.0 mL min^{-1}

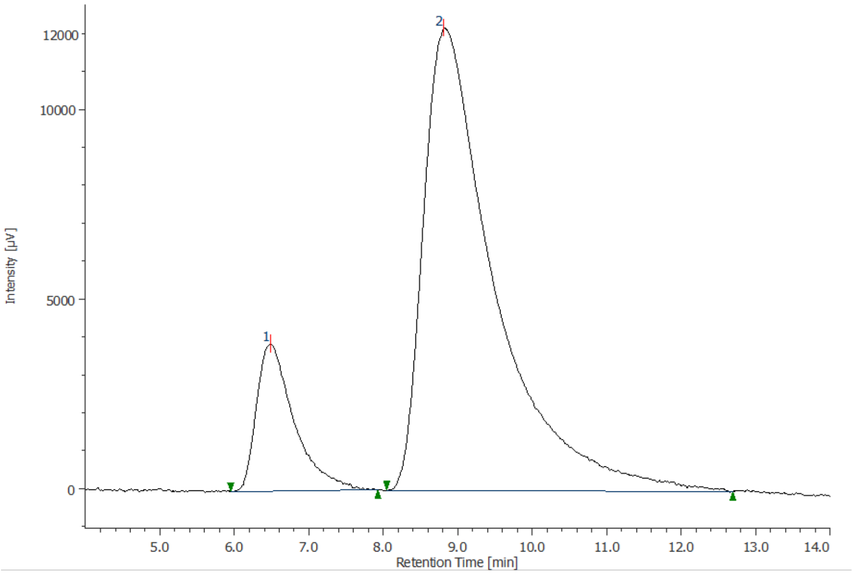

Peak No.	Retention time (min)	Area (%)
1	6.483	14.326
2	8.808	85.674

(±)-**11**
CHIRALPAK IE-3, n-hexane/CH_2Cl_2 = 30:70, 1.0 mL min^{-1}

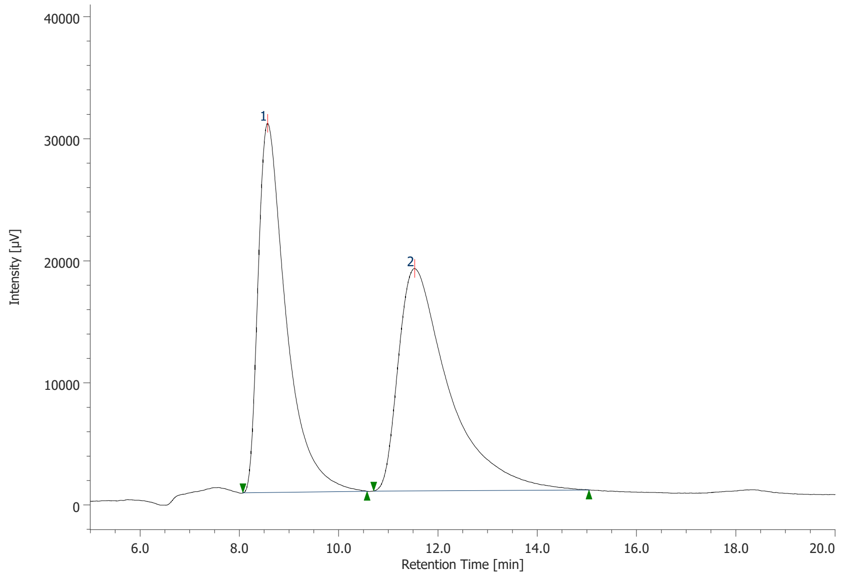

Peak No.	Retention time (min)	Area (%)
1	8.570	47.988
2	11.530	52.012

(–)-**11**
CHIRALPAK IE-3, n-hexane/CH_2Cl_2 = 30:70, 1.0 mL min^{-1}

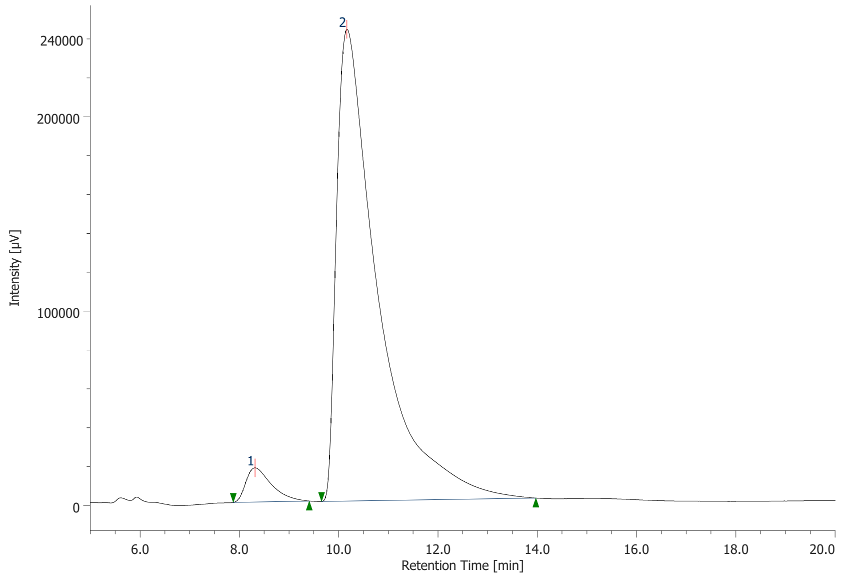

Peak No.	Retention time (min)	Area (%)
1	8.317	4.273
2	10.167	95.727

(±)-**14**

CHIRALPAK IE-3, CH_2Cl_2, 0.75 mL min^{-1}

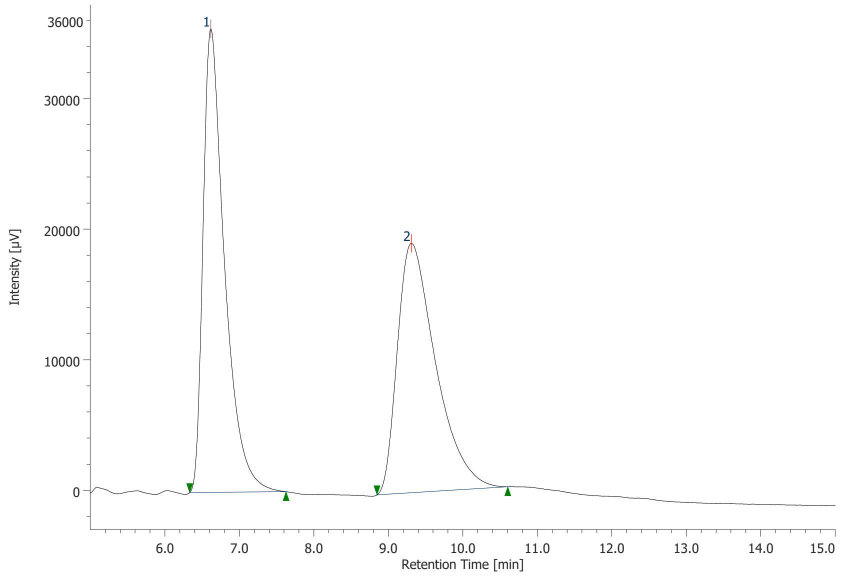

Peak No.	Retention time (min)	Area (%)
1	6.617	51.105
2	9.307	48.895

(–)-**14**

CHIRALPAK IE-3, CH_2Cl_2, 0.75 mL min^{-1}

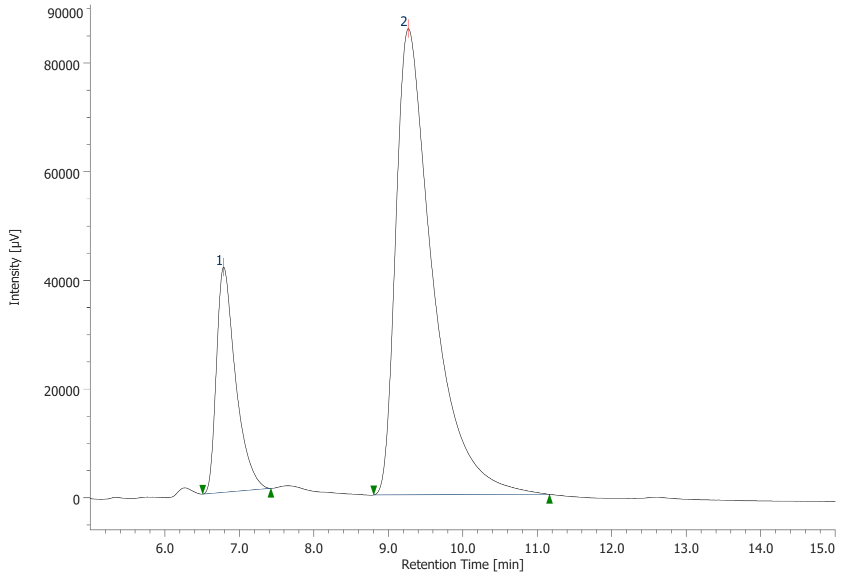

Peak No.	Retention time (min)	Area (%)
1	6.790	20.068
2	9.267	79.932

7. Photophysical and Chiroptical Properties

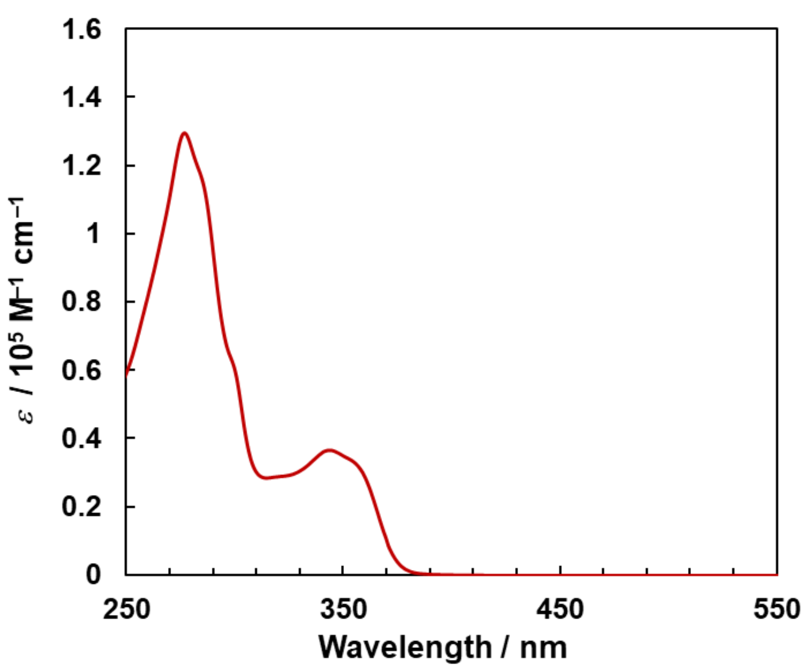

Figure S10. Absorption spectrum of **2aa** (1.0×10^{-5} M in CH_2Cl_2 at 25 °C).

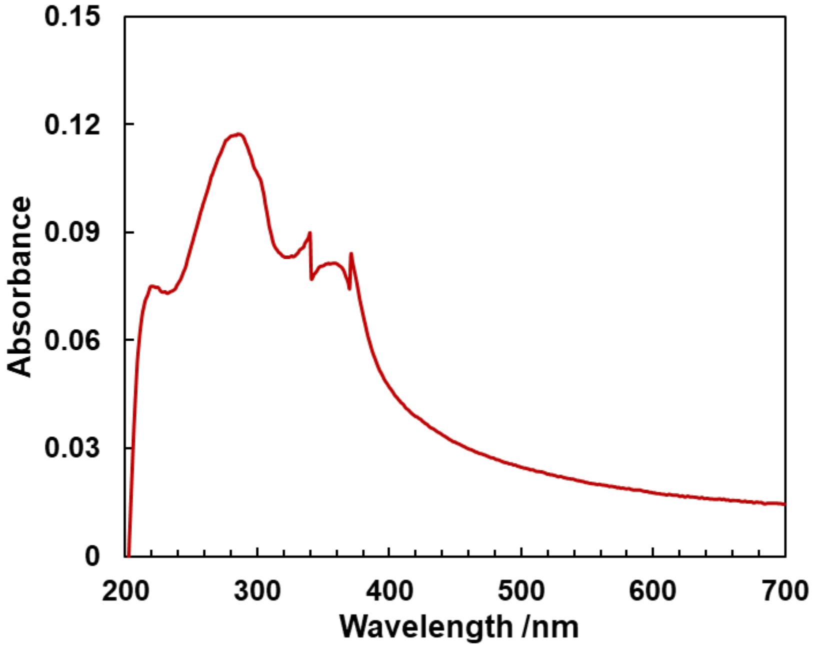

Figure S11. Absorption spectrum of **2aa** in KBr pellet.

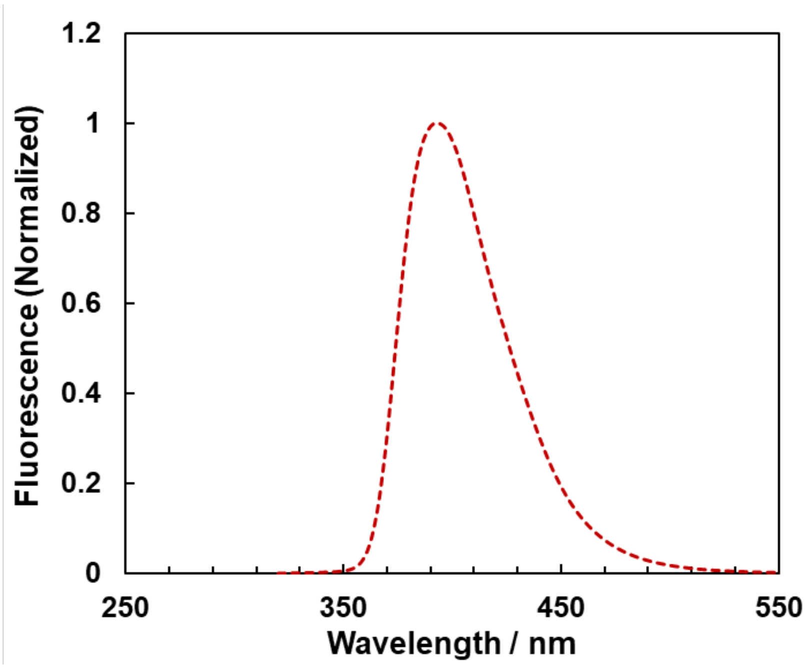

Figure S12. Emission spectrum of **2aa** (excitation wavelength: 370 nm, 1.0 × 10^{-5} M in CH_2Cl_2 at 25 °C).

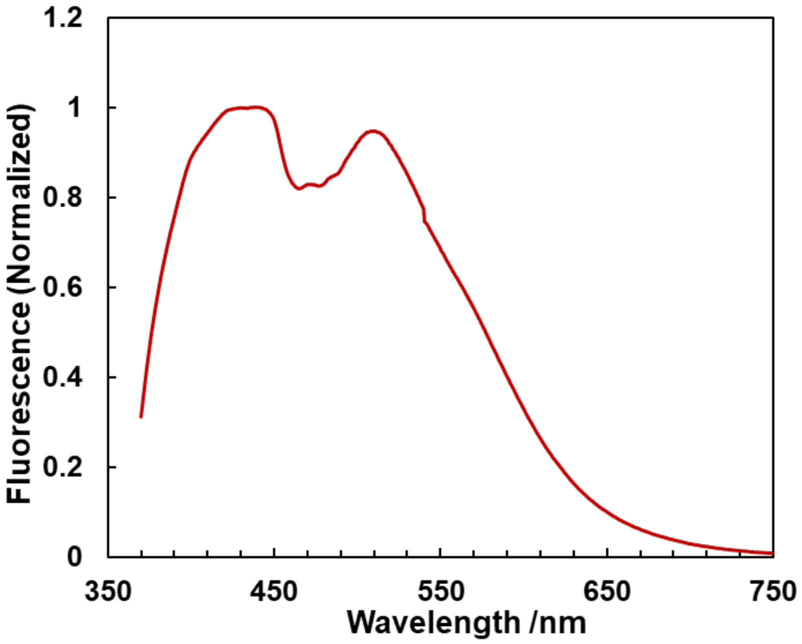

Figure S13. Emission spectrum of chiral crystal of (–)-**2aa** (excitation wavelength: 330 nm at 25 °C).

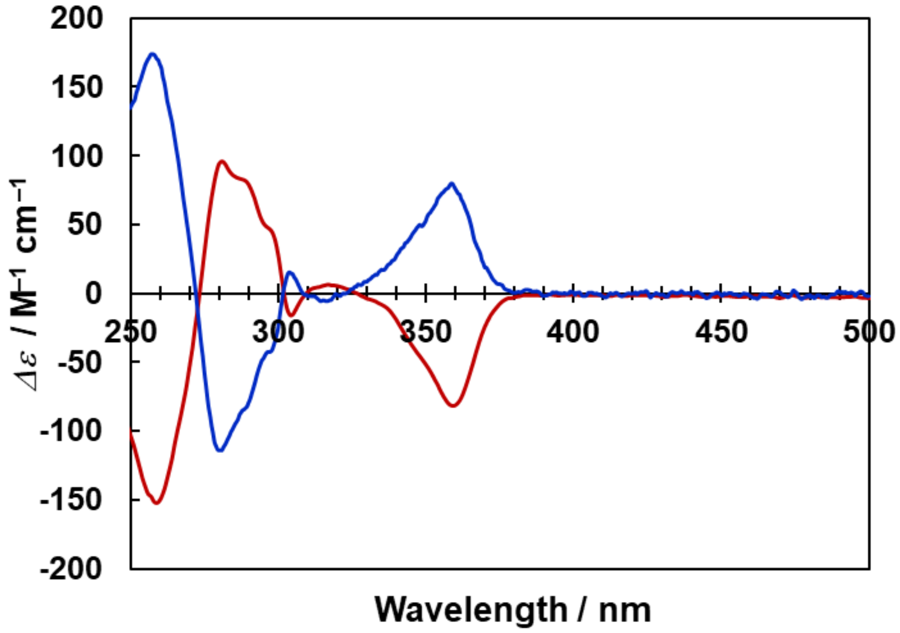

Figure S14. ECD spectrum of **2aa** (1.0×10^{-5} M in CH_2Cl_2 at 25 °C). blue line: (+)-**2aa**, red line: (−)-**2aa**.

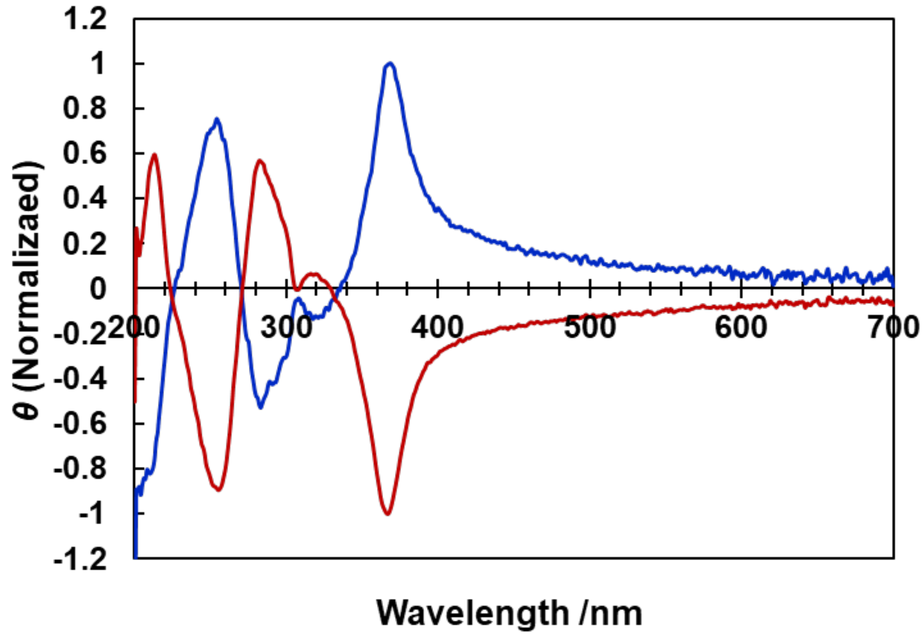

Figure S15. ECD spectrum of **2aa** in KBr pellets. Blue line: (+)-**2aa**, red line: (−)-**2aa**.

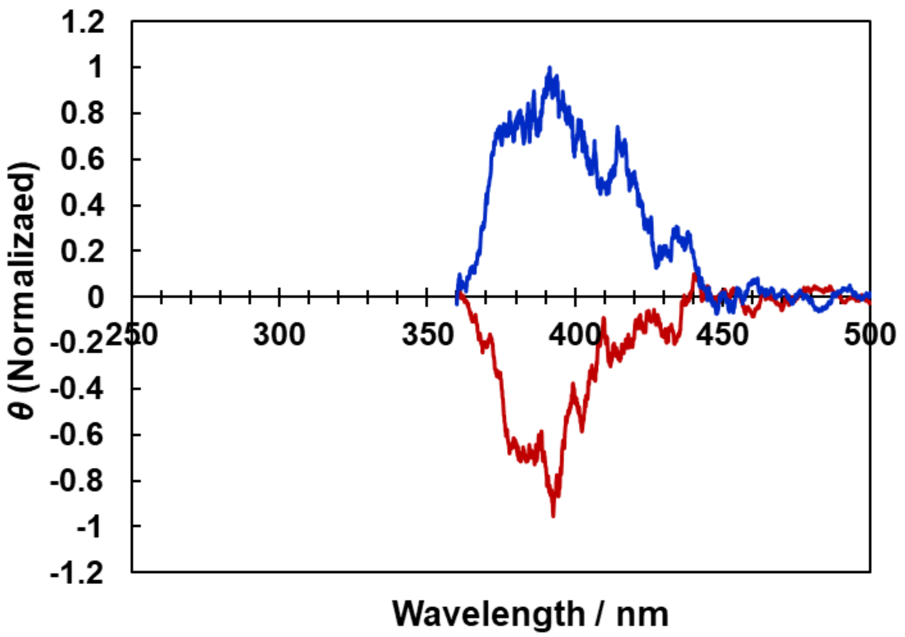

Figure S16. CPL spectrum of **2aa** (excitation wavelength: 330 nm, 1.0 × 10^{-5} M in CH_2Cl_2 at 25 °C). blue line: (+)-**2aa**, red line: (–)-**2aa**.

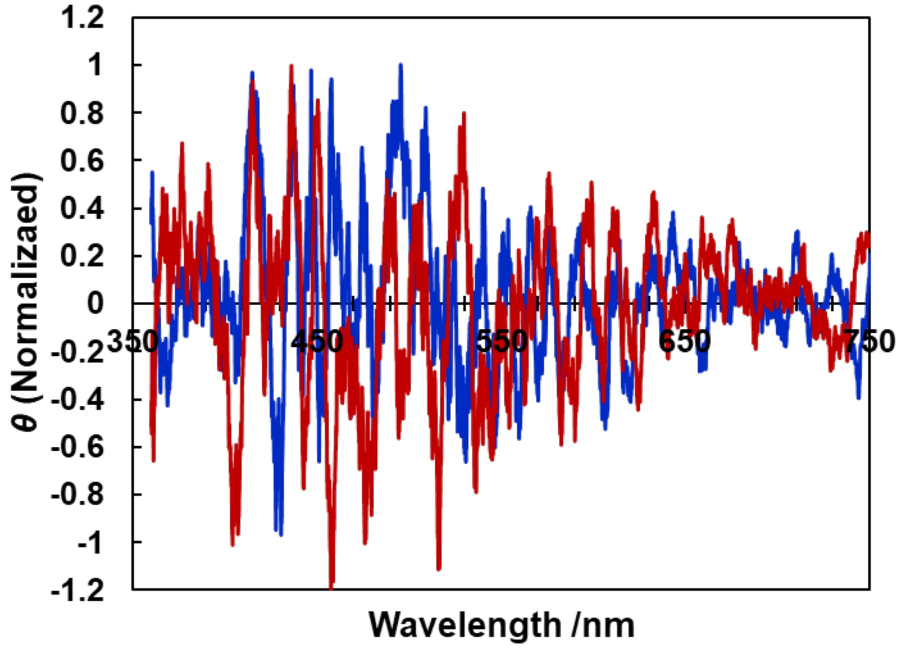

Figure S17. CPL spectrum of chiral crystal of **2aa** (excitation wavelength: 330 nm at 25 °C). blue line: (+)-**2aa**, red line: (−)-**2aa**.

8. X-Ray Crystallographic Analyses

Details of the crystal data and the summaries of the intensity data collection parameters for (±)-**2aa**, (–)-**2aa**, and (–)-**2ba** are listed in Tables S1–S3.

Single-crystal X-ray structure analysis of (±)-2aa.

Single crystals suitable for X-ray analyses were grown by diffusing hexane into a $(CH_2Cl)_2$ solution of (±)-**2aa**.

A colorless block-shaped single crystal of (±)-**2aa**, 0.41 × 0.20 × 0.17 mm^3, was selected for single-crystal diffraction measurements. The diffraction data were collected using an XtaLAB Mini II diffractometer equipped with a Hybrid Pixel Array detector with MoKα radiation by an oscillation method about the ω -axis at 268 K. X-rays were monochromated and enhanced brightness by a curved monochromator. Bragg spots were integrated, scaled, and averaged up to $\sin\theta / \lambda = 0.715$ Å^{-1} by the program CrysAlisPro.[6] Lorentz, polarization, and empirical (multi-scan) absorption corrections were applied during the scaling processes. The number of measured and independent reflections, completeness, and R_{int} was 79383, 22063, 0.948, and 0.0267, respectively.

The initial structure was solved by a dual-space method using the programs SHELXT-2018/2,[7] and refined by a full matrix least-squares method using the program SHELXL-2018/3.[8] All hydrogen atoms were put at geometrically calculated positions and refined as riding models. Some solvent molecules were heavily disordered, so we treated 369 electrons in a volume of 1140 Å^3 with the solvent mask. This is consistent with the presence of four $(CH_2Cl)_2$ per Asymmetric Unit. Fourteen reflections (-6 -2 5, -4 1 3, -3 0 3, -1 0 1, -1 1 0, -1 1 8, -1 8 6, 0 -1 1, 0 2 1, 1 -2 1, 1 0 17, 1 2 1, 4 -2 9, 5 -2 8) were omitted for least-squares due to their anomaly of larger diffraction intensities than calculated, which some unresolved overlapping of reflections could cause. The final R values against 15314 unique reflections ($\theta_{max} = 30.527°$) with $I > 2\sigma(I)$ are 0.0734, 0.1934, and 1.043 for the $R(F)$, $wR(F^2)$, and S, respectively. Crystallographic data have been deposited with the Cambridge Crystallographic Data Centre: Deposition code CCDC 2390406.

Table S1. Crystallographic data and structure refinement details for (±)-**2aa** (CCDC 2390406).

Empirical formula	$C_{80}H_{64}O_8 \cdot 0.5(C_2H_4Cl_2)$
Formula weight	1202.79
Temperature/K	268(40)
Crystal system	triclinic
Space group	P-1
a/Å	11.7994(2)
b/Å	13.9812(2)
c/Å	24.0549(3)
α/°	95.9010(10)
β/°	103.6590(10)
γ/°	95.1490(10)
V/Å^3	3808.88(10)
Z	2
ρ_X/Mg m^{-3}	1.049
μ/mm^{-1}	0.100
F(000)	1266.0
Crystal size/mm^3	0.43 × 0.203 × 0.179
Radiation	Mo Kα (λ = 0.71073 Å)
2*θ* range for data collection/°	3.578 to 61.054
Index ranges	$-16 \le h \le 16$, $-19 \le k \le 19$, $-33 \le l \le 33$
Reflections collected	79383
Independent reflections	22063 [R_{int} = 0.0267, R_{sigma} = 0.0401]
Data/restraints/parameters	22063/0/819
Goodness-of-fit on F^2	1.043
Final R indexes [$I > 2\sigma(I)$]	$R(F)$ = 0.0734, $wR(F^2)$ = 0.1936
Final R indexes [all data]	$R(F)$ = 0.0989, $wR(F^2)$ = 0.2072
Largest diff. peak/hole / e Å^{-3}	0.84/-0.41

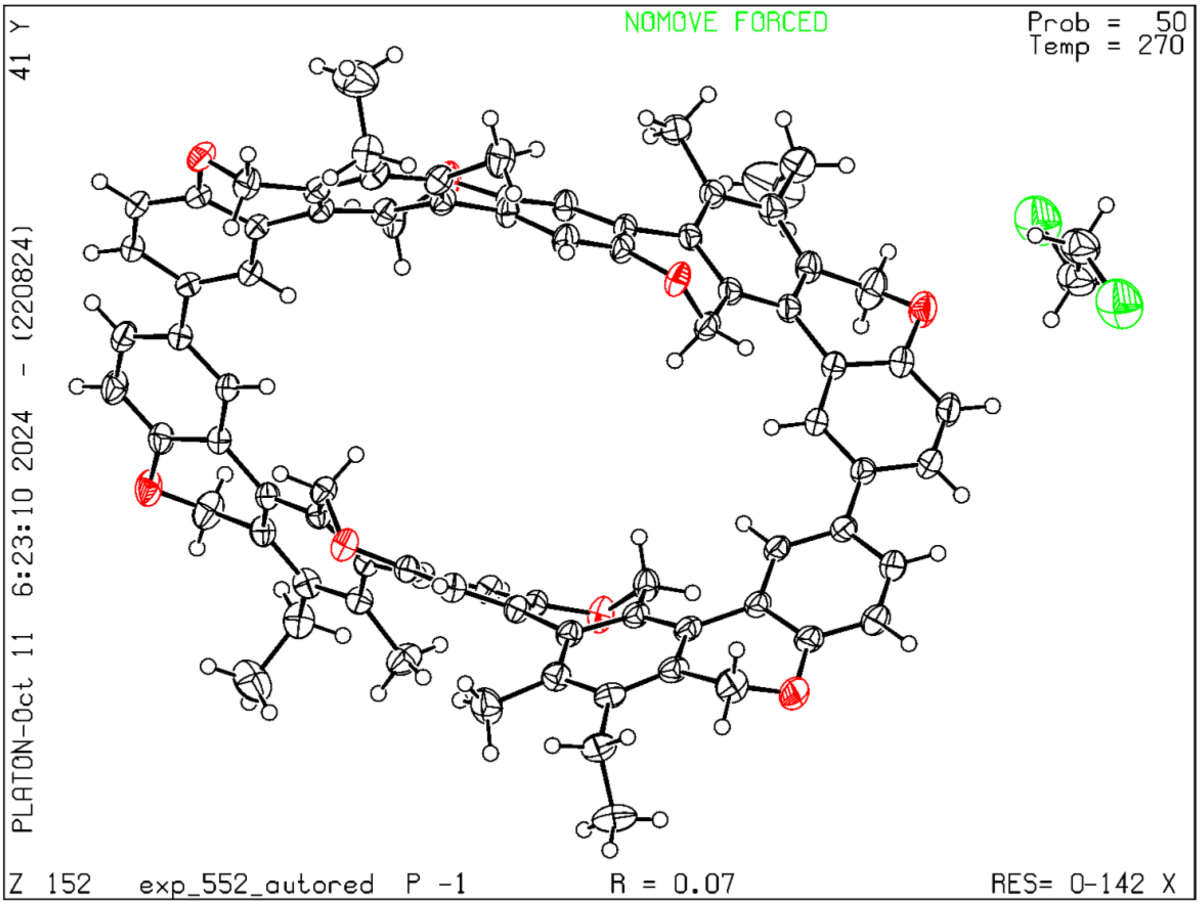

Figure S18. ORTEP diagram of (±)-**2aa** showing thermal ellipsoids at the 50% probability level.

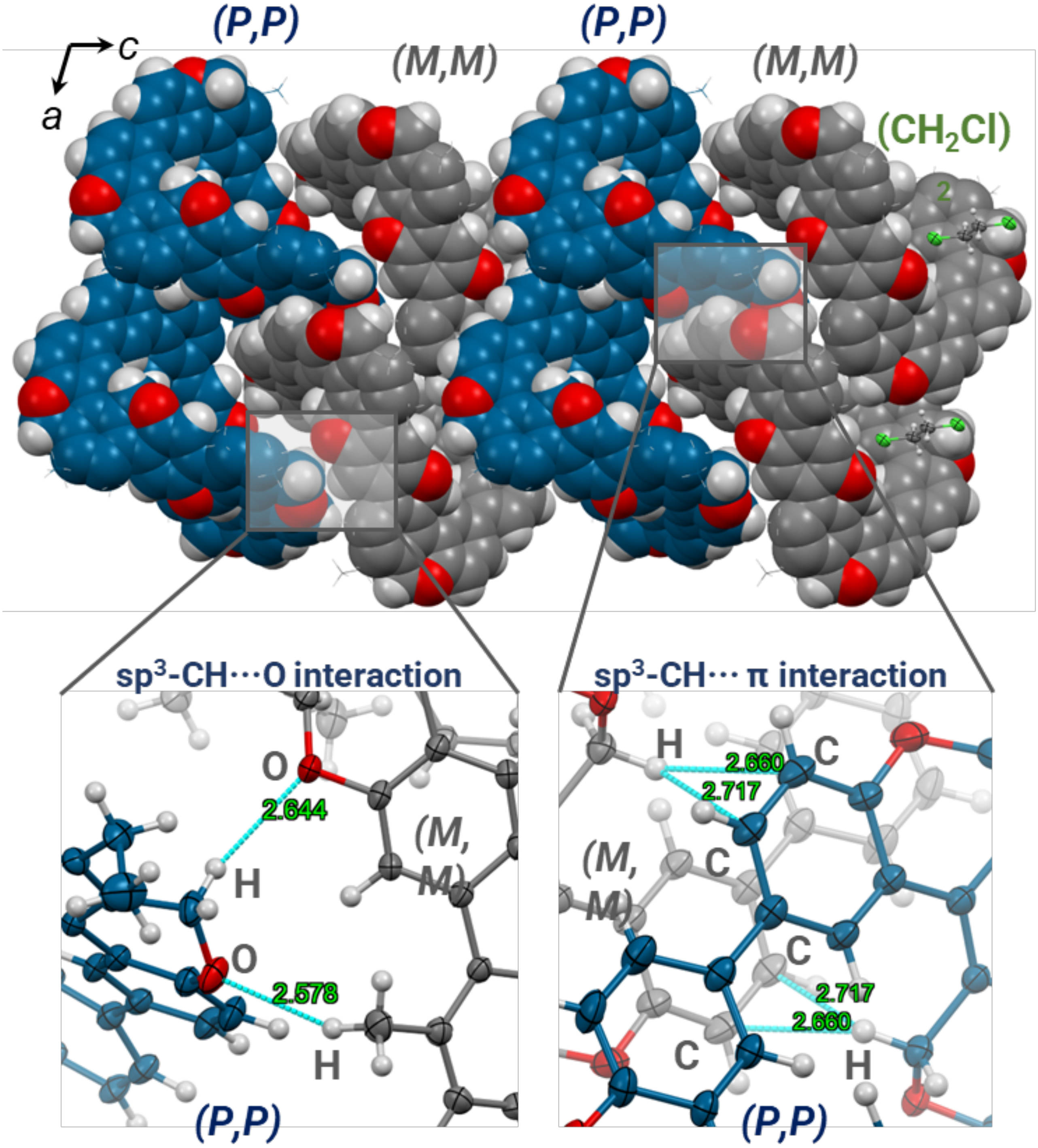

Figure S19. Packing structure of (±)-**2aa**.

Single-crystal X-ray structure analysis of (–)-2aa.

Single crystals suitable for X-ray analyses were grown by diffusing hexane into a $(CH_2Cl)_2$ solution of (−)-**2aa**.

A pale-yellow prism-shaped single crystal of (−)-**2aa**, 0.11 × 0.07 × 0.06 mm^3, was selected for single-crystal diffraction measurements. The diffraction data were collected using a RIGAKU AFC-11 diffractometer equipped with a HyPix6000HE hybrid pixel array detector with MoKα radiation by an oscillation method about the ω-axis at 89.8(4) K. X-rays were monochromated and focused by a confocal mirror. Bragg spots were integrated, scaled, and averaged up to $\sin\theta / \lambda$ = 0.909 Å^{-1} by the program CrysAlisPro.[6] Lorentz, polarization, and multi-scan absorption corrections were applied during the scaling processes. The number of measured and independent reflections, completeness, and R_{int} was 805880, 54048, 1.000, and 0.0686, respectively.

The initial structure was solved by a dual-space method using the programs SHELXT-2018/2,[7] and refined by a full matrix least-squares method using the program SHELXL-2019/2[8] Some hydrogen atoms were located on difference Fourier maps and the others were put at geometrically calculated positions, and refined as riding models. After the refinements, the contribution of non-periodically aligned solvent molecules to the intensities of reflections was removed with the SQUEEZE program.[9] The solvent-accessible voids were surveyed with a void probe radius of 1.20 Å. The contribution of 579 electrons/unit cell, which is estimated about 15 dichloroethane molecules was removed from the reflection data. The refinements were performed using the reflection data after treatment of the SQUEEZE program with the same procedure described above being the structure converged. The total number of restraints was 172. The Flack parameter (χ) was 0.008(5). The final R values against 47698 unique reflections (θ_{max} = 40.25°) with $I > 2\sigma(I)$ are 0.0587, 0.1388, and 1.055 for the $R(F)$, $wR(F^2)$, and S, respectively. Crystallographic data have been deposited with the Cambridge Crystallographic Data Centre: Deposition code CCDC 2388999.

Table S2. Crystallographic data and structure refinement details for (–)-**2aa** (CCDC 2388999).

Empirical formula	$C_{80}H_{64}O_8 \cdot 4(C_2H_4Cl_2) \cdot 2(C_2H_4Cl_2)$
Formula weight	1747.02
Temperature/K	89.8(4)
Crystal system	Hexagonal
Space group	$P6_5$
a/Å	24.69474(13)
c/Å	24.70094(16)
V/Å^3	13045.26(16)
Z	2
ρ_X/Mg m^{-3}	1.334
μ/mm^{-1}	0.437
F(000)	5448
Crystal size/mm^3	0.11 × 0.07 × 0.06
Radiation	Mo Kα (λ = 0.71073 Å)
2θ range for data collection/°	3.30 to 80.50
Index ranges	$-43 \le h \le 44$, $-44 \le k \le 35$, $-43 \le l \le 44$
Reflections collected	805880
Independent reflections	54048 [R_{int} = 0.0686]
Data/restraints/parameters	54048 / 172 / 1056
Goodness-of-fit on F^2	1.055
Final R indexes [$I > 2\sigma(I)$]	$R(F)$ = 0.0587, $wR(F^2)$ = 0.1388
Final R indexes [all data]	$R(F)$ = 0.0673, $wR(F^2)$ = 0.1435
Largest diff. peak/hole / e Å^{-3}	0.783 / −0.639
Absolute structure parameter	0.008(5)

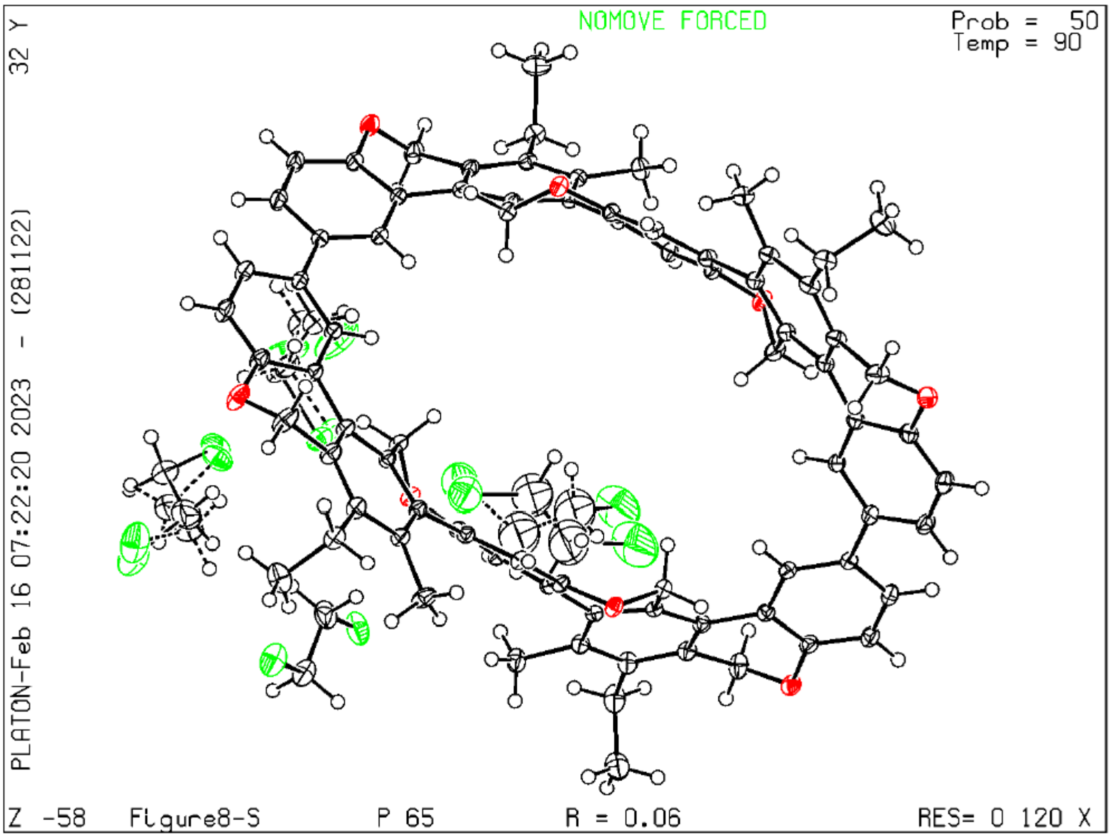

Figure S20. ORTEP diagram of (–)-**2aa** showing thermal ellipsoids at the 50% probability level.

Single-crystal X-ray structure analysis of (–)-2ba.

Single crystals suitable for X-ray analyses were grown by diffusing MeOH into a $(CH_2Cl)_2$ solution of (−)-**2ba**.

A colorless block-shaped single crystal of (−)-**2ba**, 0.33 × 0.22 × 0.16 mm^3, was selected for single-crystal diffraction measurements. The diffraction data were collected using a RIGAKU AFC-11 diffractometer equipped with a HyPix6000HE hybrid pixel array detector with MoKα radiation by an oscillation method about the ω-axis at 89.9(5) K. X-rays were monochromated and focused by a confocal mirror. Bragg spots were integrated, scaled, and averaged up to $\sin\theta$ / λ= 0.851 Å^{-1} by the program CrysAlisPro.[6] Lorentz, polarization, and multi-scan absorption corrections were applied during the scaling processes. The number of measured and independent reflections, completeness, and R_{int} was 1564432, 122162, 1.000, and 0.0629, respectively.

The initial structure was solved by a dual-space method using the programs SHELXT-2018/2,[7] and refined by a full matrix least-squares method using the program SHELXL-2019/3.[8] Some hydrogen atoms were located on difference Fourier maps and the others were put at geometrically calculated positions, and refined as riding models. After the refinements, the contribution of non-periodically aligned solvent molecules to the intensities of reflections was removed with the SQUEEZE program.[9] The solvent-accessible voids were surveyed with a void probe radius of 1.20 Å. The contribution of 2710 electrons/unit cell, which corresponds to about 55 dichloroethane molecules was removed from the reflection data. The refinements were performed using the reflection data after treatment of the SQUEEZE program with the same procedure described above being the structure converged. The total number of restraints was 158. The final R values against 105467 unique reflections (θ_{max} = 37.21°) with $I > 2\sigma(I)$ are 0.0916, 0.2321, and 1.026 for the $R(F)$, $wR(F^2)$, and S, respectively. Crystallographic data have been deposited with the Cambridge Crystallographic Data Centre: Deposition code CCDC 2389753.

Table S3. Crystallographic data and structure refinement details for (–)-**2ba** (CCDC 2389753).

Empirical formula	$5(C_{88}H_{80}O_8) \cdot 2(C_2H_4Cl_2)$
Formula weight	6525.48
Temperature/K	89.9(5)
Crystal system	Orthorhombic
Space group	$P2_12_12$
a/Å	21.8051(2)
b/Å	50.0306(6)
c/Å	22.40284(17)
V/Å^3	24439.8(4)
Z	2
ρ_X/Mg m^{-3}	0.887
μ/mm^{-1}	0.077
F(000)	6920
Crystal size/mm^3	0.33 × 0.22 × 0.16
Radiation	Mo Kα (λ = 0.71073 Å)
2θ range for data collection/°	3.07 to 74.42
Index ranges	$-36 \le h \le 36$, $-84 \le k \le 83$, $-38 \le l \le 37$
Reflections collected	1564432
Independent reflections	122162 [R_{int} = 0.0629]
Data/restraints/parameters	122162 / 158 / 2272
Goodness-of-fit on F^2	1.026
Final R indexes [$I > 2\sigma(I)$]	$R(F)$ = 0.0916, $wR(F^2)$ = 0.2321
Final R indexes [all data]	$R(F)$ = 0.1010, $wR(F^2)$ = 0.2388
Largest diff. peak/hole / e Å^{-3}	1.155 / −0.713
Absolute structure parameter	0.084(6)

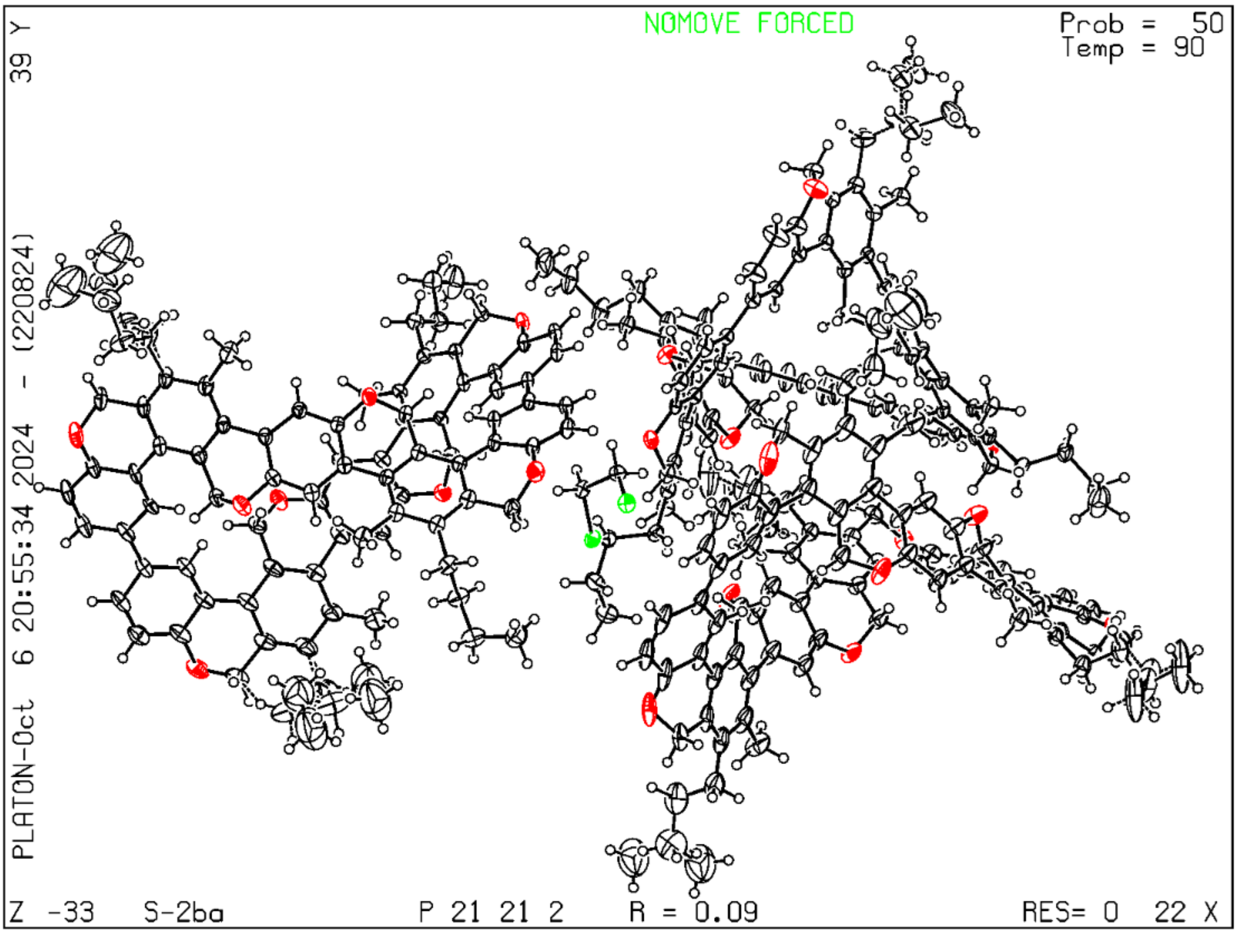

Figure S21. ORTEP diagram of (–)-**2ba** showing thermal ellipsoids at the 50% probability level.

The molecules form a tubular unit consisting of five molecules in the crystal, arranged in a body-centered orthorhombic. In the units, the molecules are stacked with a counterclockwise shift in direction, and the chirality of the units is inverted from the chirality of the molecules. The crystalline solvent, 1,2-dichloroethane, is incorporated between the units, interacting with four units per molecule. The presence of multiple CH···O interactions and CH···π interactions between neighboring molecules in the units was also suggested.

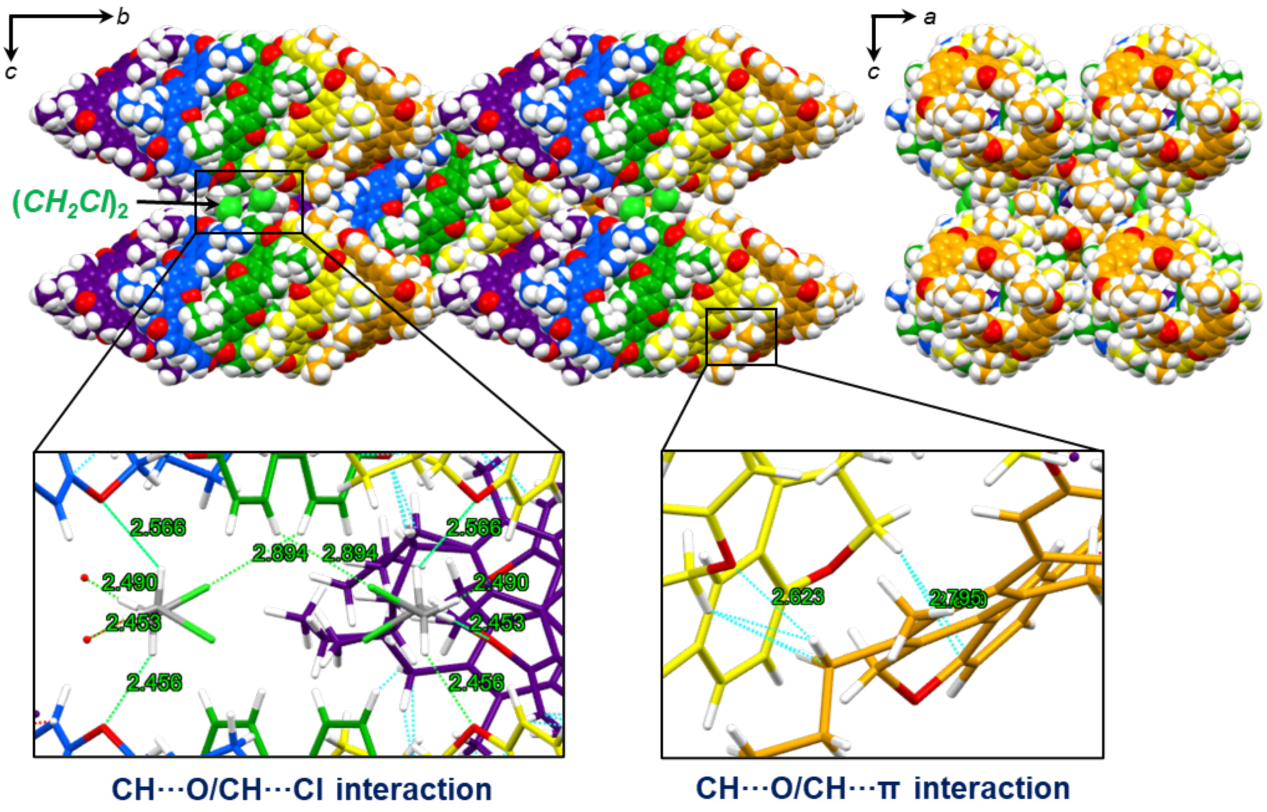

Figure S22. Packing structure of (–)**-2ba**

9. Theoretical Calculations

All calculations were carried out using the Gaussian 16 program.[10] The hybrid density functional method based on B3LYP[11,12] with 6-31g(d) basis set was used for geometry optimizations. To simplify the calculation, all alkyl groups were replaced by the methyl groups (**2-Me8**). **2-Me8** was optimized with D2 symmetry assumption. Harmonic vibration frequency calculations at the same level were performed to verify all stationary points as local minima (with no imaginary frequency). Optimized structure and frontier molecular orbital of **2-Me8** are shown in Figures S20 and Table S6. Cartesian coordinates of optimized **2-Me8** and **S1−S3** are listed in Tables S6–S9. Strain energies were calculated according to the reported procedure.[13] Zero-point energy, enthalpy, and Gibbs free energy at 298.15 K and 1 atm were calculated by B3LYP/6-31G(d) level of theory for estimation of strain energy and estimated from the gas-phase studies. The results are shown in Figure S19.

Time-dependent (TD) DFT calculations were carried out at the B3LYP/6-31G(d) level of theory based on the optimized structure. TD-DFT vertical one-electron excitations calculated are summarized in Tables S4 and S5.

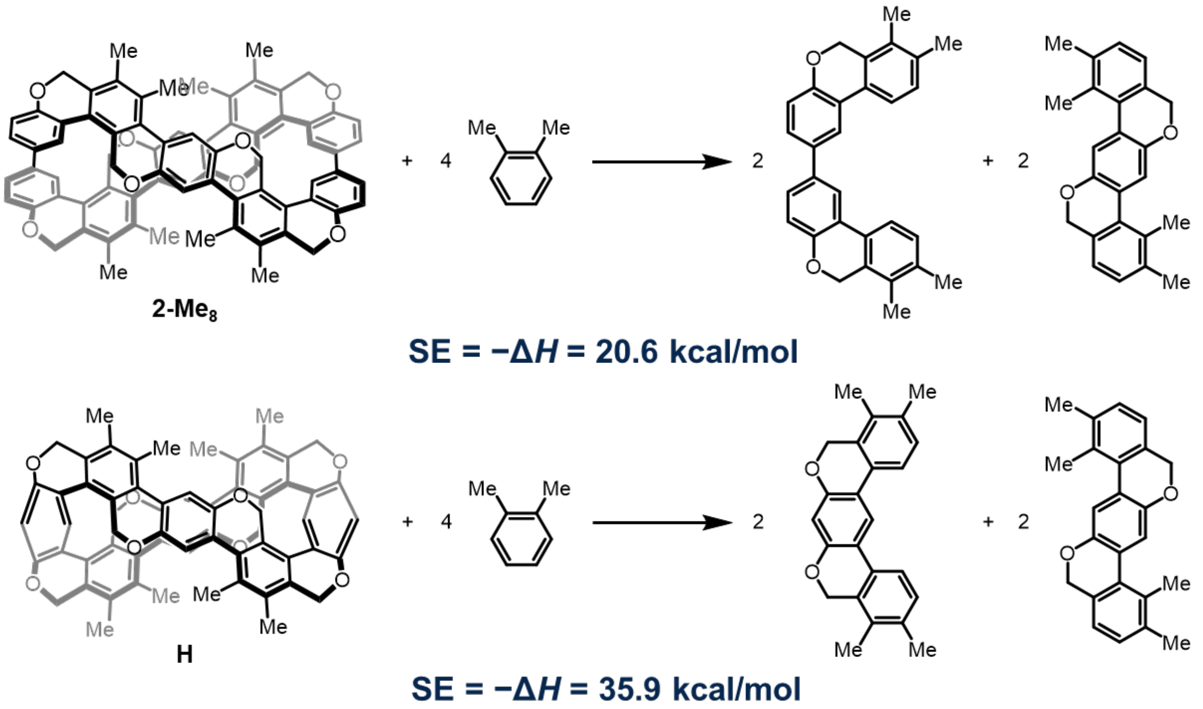

Figure S23. Homodesmotic reactions for calculating strain energies of **2-Me8** and **H**[14] at the B3LYP/6-31G(d) level.

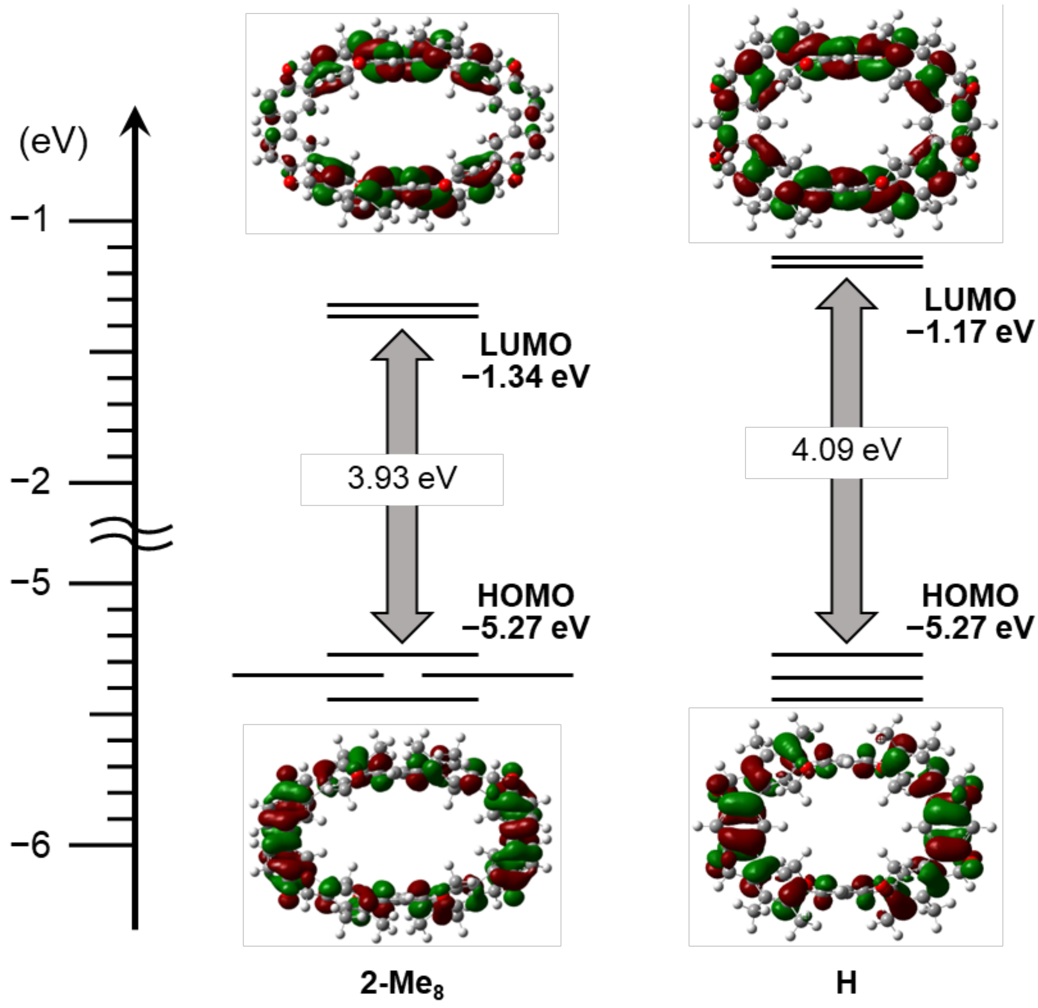

Figure S24. The energy diagram and the pictorial representation of frontier molecular orbitals of **2-Me**$_8$ and **H**[14] calculated by the DFT method at the B3LYP/6-31G(d) level of theory.

Table S4. TD-DFT vertical one-electron excitations calculated for (*M,M*)-**2**.

exited state	energy (eV)	wavelength (nm)	oscillator strength (*f*) [a]	description [b]
1	3.5021	354.02	0.2243	HOMO–4 → LUMO+1 (0.07635) (1.16%)<br>HOMO–3 → LUMO (0.28018) (15.7%)<br>HOMO–2 → LUMO+7 (0.08003) (1.28%)<br>HOMO–1 → LUMO+1 (0.43957) (38.7%)<br>HOMO → LUMO (0.42938) (37.0%)<br>HOMO → LUMO+3 (–0.07794) (1.21%)
2	3.5414	350.10	0.4401	HOMO–4 → LUMO (0.07704) (1.19%)<br>HOMO–3 → LUMO+1 (0.24135) (11.7%)<br>HOMO–1 → LUMO (0.47808) (45.9%)<br>HOMO → LUMO+1 (0.43502) (38.0%)
4	3.6061	343.81	0.0105	HOMO–3 → LUMO+2 (–0.13035) (3.41%)<br>HOMO–2 → LUMO (0.67446) (91.3%)<br>HOMO → LUMO+2 (0.0844) (1.43%)
5	3.6089	343.55	0.0276	HOMO–4 → LUMO (–0.07999) (1.28%)<br>HOMO–3 → LUMO+1 (–0.26989) (14.6%)<br>HOMO–1 → LUMO (–0.34227) (23.5)<br>HOMO → LUMO+1 (0.53521) (57.5%)
8	3.7337	332.07	0.0246	HOMO–3 → LUMO+1 (0.58206) (68.0%)<br>HOMO–1 → LUMO (–0.37794) (28.7%)<br>HOMO → LUMO+1 (0.0816) (1.34%)
9	3.7603	329.72	0.0264	HOMO–7 → LUMO (0.08035) (1.30%)<br>HOMO–5 → LUMO+1 (–0.09322) (1.74%)<br>HOMO–3 → LUMO+2 (0.08584) (1.48%)<br>HOMO–2 → LUMO+3 (0.24032) (11.6%)<br>HOMO–2 → LUMO+6 (–0.0724) (1.05%)<br>HOMO–1 → LUMO+4 (–0.1994) (7.98%)<br>HOMO → LUMO+2 (0.58736) (69.3%)<br>HOMO → LUMO+7 (–0.08851) (1.57%)
12	3.8744	320.01	0.2029	HOMO–9 → LUMO+1 (–0.10163) (2.07%)<br>HOMO–8 → LUMO (–0.08729) (1.53%)<br>HOMO–4 → LUMO (0.11004) (2.43%)<br>HOMO–3 → LUMO+5 (0.10503) (2.21%)<br>HOMO–2 → LUMO+4 (–0.15298) (4.70%)<br>HOMO–1 → LUMO+3 (0.52834) (55.1%)<br>HOMO → LUMO+1 (0.07915) (1.26%)<br>HOMO → LUMO+5 (0.07915) (26.0%)

16	4.0115	309.07	0.1247	HOMO–5 → LUMO+7 (0.07809) (1.23%)<br>HOMO–4 → LUMO (–0.07315) (1.08%)<br>HOMO–4 → LUMO+6 (–0.07261) (1.06%)<br>HOMO–3 → LUMO+5 (0.21179) (9.02%)<br>HOMO–2 → LUMO+4 (0.47024) (44.4%)<br>HOMO–1 → LUMO+3 (0.32321) (21.0%)<br>HOMO → LUMO+5 (–0.2936) (17.3%)

[a] Excitation energies with oscillator strength larger than 0.01 are listed. [b] Relative contribution larger than 1% is listed.

Table S5. Transition moments of (*M,M*)-**2**.

<table>
<tr><td colspan="7">Ground to excited state transition electric dipole moments (Au):</td></tr>
<tr><td>state</td><td>X</td><td>Y</td><td>Z</td><td>Dip. S.</td><td>Osc.</td><td></td></tr>
<tr><td>1</td><td>0</td><td>0</td><td>1.6168</td><td>2.614</td><td>0.2243</td><td></td></tr>
<tr><td colspan="7">Ground to excited state transition velocity dipole moments (Au):</td></tr>
<tr><td>state</td><td>X</td><td>Y</td><td>Z</td><td>Dip. S.</td><td>Osc.</td><td></td></tr>
<tr><td>1</td><td>0</td><td>0</td><td>-0.2081</td><td>0.0433</td><td>0.2244</td><td></td></tr>
<tr><td colspan="7">Ground to excited state transition magnetic dipole moments (Au):</td></tr>
<tr><td>state</td><td>X</td><td>Y</td><td>Z</td><td></td><td></td><td></td></tr>
<tr><td>1</td><td>0</td><td>0</td><td>4.7127</td><td></td><td></td><td></td></tr>
<tr><td colspan="7">Ground to excited state transition velocity quadrupole moments (Au):</td></tr>
<tr><td>state</td><td>XX</td><td>YY</td><td>ZZ</td><td>XY</td><td>XZ</td><td>YZ</td></tr>
<tr><td>1</td><td>0</td><td>0</td><td>0</td><td>-1.1713</td><td>0</td><td>0</td></tr>
<tr><td colspan="7"><0|del|b> * <b|rxdel|0> + <0|del|b> * <b|delr+rdel|0></td></tr>
<tr><td colspan="7">Rotatory Strengths (R) in cgs (10**-40 erg-esu-cm/Gauss)</td></tr>
<tr><td>state</td><td>XX</td><td>YY</td><td>ZZ</td><td>R(velocity)</td><td>E-M Angle</td><td></td></tr>
<tr><td>1</td><td>-2025.0273</td><td>-3364.5454</td><td>0</td><td>-1796.5242</td><td>90</td><td></td></tr>
<tr><td colspan="7">1/2[<0|r|b>*<b|rxdel|0> + (<0|rxdel|b>*<b|r|0>)*]</td></tr>
<tr><td colspan="7">Rotatory Strengths (R) in cgs (10**-40 erg-esu-cm/Gauss)</td></tr>
<tr><td>state</td><td>XX</td><td>YY</td><td>ZZ</td><td>R(length)</td><td></td><td></td></tr>
<tr><td>1</td><td>0</td><td>0</td><td>-5388.2276</td><td>-1796.0759</td><td></td><td></td></tr>
<tr><td colspan="7">1/2[<0|del|b>*<b|r|0> + (<0|r|b>*<b|del|0>)*] (Au)</td></tr>
<tr><td>state</td><td>X</td><td>Y</td><td>Z</td><td>Dip. S.</td><td>Osc.(frdel)</td><td></td></tr>
<tr><td>1</td><td>0</td><td>0</td><td>-0.3365</td><td>0.3365</td><td>0.2243</td><td></td></tr>
</table>

Table S6. Cartesian coordinates of optimized (*M,M*)-**2** [B3LYP/6-31g(d) level of theory].

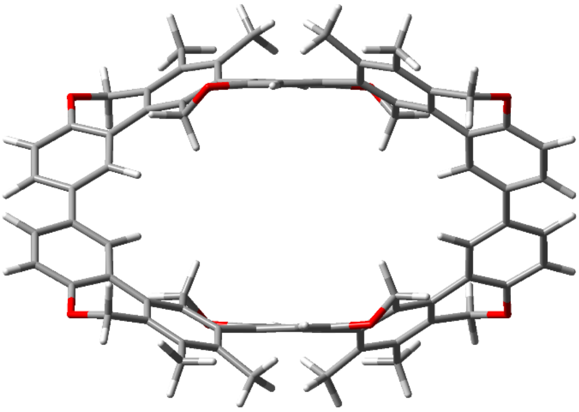

C	8.099258	1.866135	2.179382	C	-2.27865	4.53999	-3.11003
C	8.09753	0.880774	1.199622	C	-3.41567	4.374396	-3.92863
C	6.918127	0.537999	0.514312	C	-3.37221	2.875124	0.123425
C	5.749584	1.2455	0.842417	O	-2.5915	3.784832	0.906055
C	5.719491	2.26897	1.80338	C	-1.30202	3.875231	0.44592
C	6.920113	2.53708	2.49326	C	-1.0737	3.936071	-0.94179
C	4.522973	3.067381	2.169359	C	-0.26257	3.888646	1.369083
C	4.518724	3.65479	3.454082	C	1.073703	3.936071	0.941787
C	5.768466	3.484863	4.290227	C	1.302018	3.875231	-0.44592
O	6.955283	3.471037	3.489601	C	0.262571	3.888646	-1.36908
C	3.406101	3.274264	1.339556	C	-1.15891	5.441627	-3.58986
C	2.256122	3.931952	1.835278	C	-3.42972	4.968232	-5.32585
C	2.278646	4.53999	3.110032	C	8.099258	-1.86614	-2.17938
C	3.415672	4.374396	3.928633	C	8.09753	-0.88077	-1.19962
C	3.372205	2.875124	-0.12343	C	6.918127	-0.538	-0.51431
O	2.591496	3.784832	-0.90606	C	5.749584	-1.2455	-0.84242
C	1.158909	5.441627	3.589856	C	5.719491	-2.26897	-1.80338
C	3.429724	4.968232	5.325851	C	6.920113	-2.53708	-2.49326
C	-8.09926	1.866135	-2.17938	C	4.522973	-3.06738	-2.16936
C	-8.09753	0.880774	-1.19962	C	4.518724	-3.65479	-3.45408
C	-6.91813	0.537999	-0.51431	C	5.768466	-3.48486	-4.29023
C	-5.74958	1.2455	-0.84242	O	6.955283	-3.47104	-3.4896
C	-5.71949	2.26897	-1.80338	C	3.406101	-3.27426	-1.33956
C	-6.92011	2.53708	-2.49326	C	2.256122	-3.93195	-1.83528
C	-4.52297	3.067381	-2.16936	C	2.278646	-4.53999	-3.11003
C	-4.51872	3.65479	-3.45408	C	3.415672	-4.3744	-3.92863
C	-5.76847	3.484863	-4.29023	C	3.372205	-2.87512	0.123425
O	-6.95528	3.471037	-3.4896	O	2.591496	-3.78483	0.906055
C	-3.4061	3.274264	-1.33956	C	1.158909	-5.44163	-3.58986
C	-2.25612	3.931952	-1.83528	C	3.429724	-4.96823	-5.32585

C	-8.09926	-1.86614	2.179382
C	-8.09753	-0.88077	1.199622
C	-6.91813	-0.538	0.514312
C	-5.74958	-1.2455	0.842417
C	-5.71949	-2.26897	1.80338
C	-6.92011	-2.53708	2.49326
C	-4.52297	-3.06738	2.169359
C	-4.51872	-3.65479	3.454082
C	-5.76847	-3.48486	4.290227
O	-6.95528	-3.47104	3.489601
C	-3.4061	-3.27426	1.339556
C	-2.25612	-3.93195	1.835278
C	-2.27865	-4.53999	3.110032
C	-3.41567	-4.3744	3.928633
C	-3.37221	-2.87512	-0.12343
O	-2.5915	-3.78483	-0.90606
C	-1.30202	-3.87523	-0.44592
C	-1.0737	-3.93607	0.941787
C	-0.26257	-3.88865	-1.36908
C	1.073703	-3.93607	-0.94179
C	1.302018	-3.87523	0.44592
C	0.262571	-3.88865	1.369083
C	-1.15891	-5.44163	3.589856
C	-3.42972	-4.96823	5.325851
H	8.999158	2.107022	2.736411
H	9.016741	0.337713	1.005587
H	4.831424	0.979442	0.336628
H	5.913793	4.317937	4.977473
H	5.730882	2.55418	4.882209
H	4.369944	2.908715	-0.5621
H	2.963264	1.860422	-0.26624
H	0.562999	5.818359	2.757627
H	0.472022	4.937358	4.283928
H	1.564865	6.308103	4.121052
H	4.070072	4.403386	6.007572
H	3.786244	6.008109	5.327815
H	2.430333	4.971449	5.767943
H	-8.99916	2.107022	-2.73641
H	-9.01674	0.337713	-1.00559
H	-4.83142	0.979442	-0.33663
H	-5.91379	4.317937	-4.97747
H	-5.73088	2.55418	-4.88221
H	-4.36994	2.908715	0.562103
H	-2.96326	1.860422	0.266237
H	-0.51908	3.818651	2.41896
H	0.519078	3.818651	-2.41896
H	-0.563	5.818359	-2.75763
H	-0.47202	4.937358	-4.28393
H	-1.56487	6.308103	-4.12105
H	-4.07007	4.403386	-6.00757
H	-3.78624	6.008109	-5.32782
H	-2.43033	4.971449	-5.76794
H	8.999158	-2.10702	-2.73641
H	9.016741	-0.33771	-1.00559
H	4.831424	-0.97944	-0.33663
H	5.913793	-4.31794	-4.97747
H	5.730882	-2.55418	-4.88221
H	4.369944	-2.90872	0.562103
H	2.963264	-1.86042	0.266237
H	0.562999	-5.81836	-2.75763
H	0.472022	-4.93736	-4.28393
H	1.564865	-6.3081	-4.12105
H	4.070072	-4.40339	-6.00757
H	3.786244	-6.00811	-5.32782
H	2.430333	-4.97145	-5.76794
H	-8.99916	-2.10702	2.736411
H	-9.01674	-0.33771	1.005587
H	-4.83142	-0.97944	0.336628
H	-5.91379	-4.31794	4.977473
H	-5.73088	-2.55418	4.882209
H	-4.36994	-2.90872	-0.5621
H	-2.96326	-1.86042	-0.26624
H	-0.51908	-3.81865	-2.41896
H	0.519078	-3.81865	2.41896
H	-0.563	-5.81836	2.757627
H	-0.47202	-4.93736	4.283928
H	-1.56487	-6.3081	4.121052
H	-2.43033	-4.97145	5.767943
H	-4.07007	-4.40339	6.007572
H	-3.78624	-6.00811	5.327815

Table S7. Cartesian coordinates of optimized unit **S1** [B3LYP/6-31g(d) level of theory].

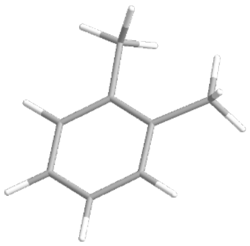

C	0	0.69675	1.960829	H	0	-1.24776	2.897616
C	0	-0.69675	1.960829	H	0	2.472229	0.745076
C	0	1.384328	0.746337	H	0	-2.47223	0.745076
C	0	0.705755	-0.47765	H	0	2.558642	-1.59057
C	0	-1.38433	0.746337	H	-0.88022	1.246944	-2.38872
C	0	-0.70576	-0.47765	H	0.880217	1.246944	-2.38872
C	0	1.480045	-1.7753	H	0	-2.55864	-1.59057
C	0	-1.48005	-1.7753	H	-0.88022	-1.24694	-2.38872
H	0	1.247758	2.897616	H	0.880217	-1.24694	-2.38872

Table S8. Cartesian coordinates of optimized unit **S2** [B3LYP/6-31g(d) level of theory].

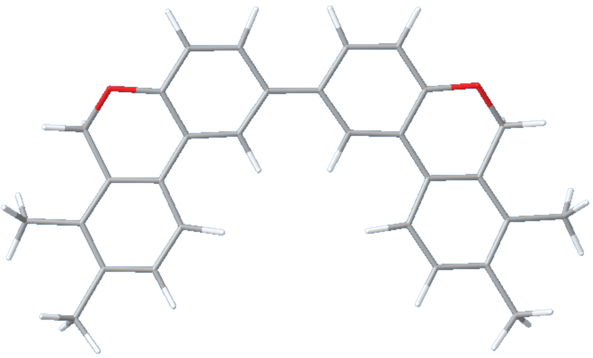

C	2.768232	-3.03115	-1.074947	C	6.311607	3.847177	0.771849
C	1.396282	-3.04587	-0.848533	C	-7.329173	1.33919	0.781421
C	0.745386	-1.946164	-0.259975	C	-6.504706	3.598307	-1.011474
C	1.518845	-0.823989	0.066757	H	3.276711	-3.878614	-1.524072
C	2.898573	-0.769357	-0.173994	H	0.818228	-3.918239	-1.139593
C	3.516889	-1.906724	-0.727256	H	1.0356	0.018826	0.551519
C	-0.715126	-1.974687	0.006511	H	-0.754874	-4.039028	0.642177
C	-1.346965	-3.146339	0.461943	H	-3.197946	-4.06117	1.111933
C	-2.709873	-3.166794	0.737393	H	-1.038158	0.067328	-0.566395
C	-3.471623	-2.012483	0.555774	H	2.134691	1.868244	0.203132
C	-2.886826	-0.830934	0.060672	H	3.627336	3.773928	0.615795
C	-1.508222	-0.835972	-0.189678	H	-4.014383	3.226427	-1.957045
C	3.734221	0.420527	0.076065	H	-2.448673	1.377458	-1.560108
C	3.206628	1.704587	0.254986	H	6.658314	-1.158176	-0.519348
C	4.053787	2.783048	0.478257	H	5.706288	-1.670928	0.885831
C	5.4452	2.631501	0.523597	H	-6.470548	-1.043731	1.289514
C	5.994269	1.345954	0.329082	H	-5.063063	-0.637209	2.288656
C	5.132899	0.260624	0.098214	H	8.035197	2.01988	0.663724
C	-3.775018	0.311758	-0.226819	H	7.882746	0.807267	-0.604944
C	-5.061291	0.314992	0.345783	H	7.763765	0.333956	1.089894
C	-5.96533	1.366034	0.119552	H	5.693885	4.744674	0.873028
C	-5.581974	2.435196	-0.718226	H	7.018355	4.025856	-0.048089
C	-4.308464	2.411134	-1.300202	H	6.903857	3.752427	1.69093
C	-3.416504	1.371209	-1.068407	H	-7.888657	2.258946	0.604161
C	5.658092	-1.145861	-0.084043	H	-7.943341	0.509421	0.406523
O	4.860297	-1.923916	-0.984777	H	-7.251298	1.213167	1.868215
O	-4.810858	-2.076793	0.824923	H	-7.443859	3.274088	-1.476858
C	-5.398752	-0.843361	1.257476	H	-6.026436	4.304345	-1.697095
C	7.492682	1.118889	0.373357	H	-6.77172	4.153895	-0.10346

Table S9. Cartesian coordinates of optimized unit **S3** [B3LYP/6-31g(d) level of theory].

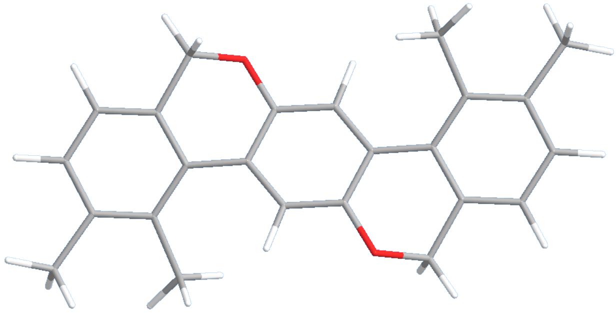

C	-4.73944	1.82118	0.204489	C	6.275549	1.64176	0.050441
C	-3.37964	1.545381	0.151979	C	3.441335	2.265926	0.364658
C	-2.35554	2.649554	0.185197	H	-5.08258	2.851193	0.272035
O	-1.25336	2.355335	-0.67994	H	-1.9698	2.810177	1.20624
C	-5.65756	0.775796	0.177822	H	-2.77628	3.591726	-0.17466
C	-5.23294	-0.54691	0.027677	H	-6.72167	0.988911	0.24446
C	-0.65529	1.163037	-0.3473	H	1.239395	1.972824	-0.81474
C	0.730644	1.094916	-0.44123	H	-1.2394	-1.97282	0.81474
C	1.43595	-0.06084	-0.06944	H	5.082577	-2.85119	-0.27204
C	0.655287	-1.16304	0.347301	H	1.969801	-2.81018	-1.20624
C	-0.73064	-1.09492	0.441229	H	2.776276	-3.59173	0.174659
C	-1.43595	0.060835	0.069443	H	6.721669	-0.98891	-0.24446
C	-2.91174	0.214283	0.065086	H	-7.26955	-1.24215	0.172351
C	-3.85246	-0.83894	-0.06645	H	-6.32339	-2.09653	-1.04886
C	2.911735	-0.21428	-0.06509	H	-6.07613	-2.4533	0.659384
C	3.852464	0.838939	0.066454	H	-2.50678	-2.30655	-0.92778
C	4.739435	-1.82118	-0.20449	H	-3.30326	-2.86534	0.546181
C	3.379636	-1.54538	-0.15198	H	-4.20563	-2.77272	-0.95985
C	2.355537	-2.64955	-0.1852	H	7.269547	1.242147	-0.17235
O	1.253358	-2.35534	0.679938	H	6.076133	2.453295	-0.65938
C	5.657559	-0.7758	-0.17782	H	6.323394	2.096533	1.048859
C	5.23294	0.546913	-0.02768	H	2.50678	2.306553	0.927775
C	-6.27555	-1.64176	-0.05044	H	4.205625	2.772717	0.95985
C	-3.44134	-2.26593	-0.36466	H	3.303263	2.86534	-0.54618

10. References

[1] A. Kar, N. Mangu, H. M. Kaiser, M. Beller, M. K. Tse, *Chem. Commun.* **2008**, *386*, 386–388.

[2] M. Zhang, A. A. Sayyad, A. Dhesi, A. Orellana, *J. Org. Chem.* **2020**, *85*, 13621–13629.

[3] T. Kippo, T. Fukuyama, I. Ryu, *Org. Lett.* **2011**, *13*, 3864–3867.

[4] C. Mitsui, H. Tanaka, H. Tsuji, E. Nakamura, *Chem. Asian J.* **2011**, *6*, 2296–2300.

[5] S. Nishigaki, Y. Shibata, A. Nakajima, H. Okajima, Y. Masumoto, T. Osawa, A. Muranaka, H. Sugiyama, A. Horikawa, H. Uekusa, H. Koshino, M. Uchiyama, A. Sakamoto, K. Tanaka, *J. Am. Chem. Soc.* **2019**, *141*, 14955–14960.

[6] Crystal data were analyzed using CrysAlisPro 1.171.43.113a (Rigaku Oxford Diffraction, 2024) for (±)-**2aa** and CrysAlisPro 1.171.42.72a (Rigaku Oxford Diffraction, 2022) for (–)-**2aa** and (–)-**2ba**.

[7] G. M. Sheldrick, *Acta Cryst.* **2015**, *A71*, 3–8.

[8] G. M. Sheldrick, *Acta Cryst.* **2015**, *C71*, 3–8.

[9] A. L. Spek, *Acta Cryst.* **2015**, *C71*, 9–18.

[10] M. J. Frisch, G. W. Trucks, H. B. Schlegel, G. E. Scuseria, M. A. Robb, J. R. Cheeseman, J. A. Montgomery Jr, T. Vreven, K. N. Kudin, J. C. Burant, Wallingford CT: Gaussian. *Gaussian 16, Revis. C.01* (2004).

[11] A. D. Becke, *J. Chem. Phys.* **1993**, *98*, 5648–5652.

[12] C. Lee, W. Yang, R. G. Parr, *Phys. Rev. B* **1988**, *37*, 785–789.

[13] Y. Segawa, A. Yagi, H. Ito, K. Itami, *Org. Lett.* **2016**, *18*, 1430–1433.

[14] J. Nogami, D. Hashizume, Y. Nagashima, K. Miyamoto, M. Uchiyama, K. Tanaka, *Nat. Synth*. **2023**, *2*, 888–897.
